# Smurfness‐based two‐phase model of ageing helps deconvolve the ageing transcriptional signature

**DOI:** 10.1111/acel.13946

**Published:** 2023-10-12

**Authors:** Flaminia Zane, Hayet Bouzid, Sofia Sosa Marmol, Mira Brazane, Savandara Besse, Julia Lisa Molina, Céline Cansell, Fanny Aprahamian, Sylvère Durand, Jessica Ayache, Christophe Antoniewski, Nicolas Todd, Clément Carré, Michael Rera

**Affiliations:** ^1^ Université Paris Cité, INSERM UMR U1284 Paris France; ^2^ Institut de Biologie Paris Seine, Sorbonne Université Paris France; ^3^ Université Paris‐Saclay, AgroParisTech, INRAE, UMR PNCA Palaiseau France; ^4^ Metabolomics and Cell Biology Platforms, UMS AMMICa Institut Gustave Roussy Villejuif France; ^5^ Centre de Recherche des Cordeliers, Equipe Labellisée par la Ligue Contre le Cancer Université de Paris, Sorbonne Université, INSERM U1138, Institut Universitaire de France Paris France; ^6^ Institut Jacques Monod, CNRS UMR 7592, Université Paris Cité Paris France; ^7^ Eco‐Anthropologie (EA), Muséum National d'Histoire Naturelle, CNRS Université de Paris, Musée de l'Homme Paris France

**Keywords:** ageing, Drosophila, end of life, lifespan increasing genetic intervention, Smurfs, transcriptome

## Abstract

Ageing is characterised at the molecular level by six transcriptional ‘hallmarks of ageing’, that are commonly described as progressively affected as time passes. By contrast, the ‘Smurf’ assay separates high‐and‐constant‐mortality risk individuals from healthy, zero‐mortality risk individuals, based on increased intestinal permeability. Performing whole body total RNA sequencing, we found that Smurfness distinguishes transcriptional changes associated with chronological age from those associated with biological age. We show that transcriptional heterogeneity increases with chronological age in non‐Smurf individuals preceding the other five hallmarks of ageing that are specifically associated with the Smurf state. Using this approach, we also devise targeted pro‐longevity genetic interventions delaying entry in the Smurf state. We anticipate that increased attention to the evolutionary conserved Smurf phenotype will bring about significant advances in our understanding of the mechanisms of ageing.

## Graphical abstract

The two‐phase model of ageing allows to study separately the effect of chronological and physiological age. (a) Classic approaches for studying ageing tend to consider it as a black box affecting all individuals progressively from birth to death. Instead, the Smurf phenotype shows that life can be divided into two consecutive phases separated by an abrupt transition. (b) All individuals undergo this transition at a different moment in their life, prior to death. This allows us to switch from population based approaches, comparing bulks of age‐matched individuals through time, to individuals‐centred approaches relying on direct access to their transition status. (c) Such paradigm shift shows that hallmarks of ageing long thought to progressively change with age are actually mostly affected in a growing proportion of Smurfs, allowing for the identification of the chain of events accompanying ageing and death from natural causes. (d) By studying the behaviour of the ageing transcriptome as a function of chronological age and Smurfness separately, we demonstrate that the progressively changing transcriptional ageing signature, as described in Frenk and Houseley (2018), is in fact the convolution changes accompanying chronological age signature (increased transcriptional noise) and changes associated with Smurfness (or biological age) signature (increased stress response and inflammation, decreased expression of ribosomal and mitochondrial genes). We also identified a hallmark partially associated with only old Smurfs (ATH5), suggesting that chronological age can affect, late in life, the Smurf response.

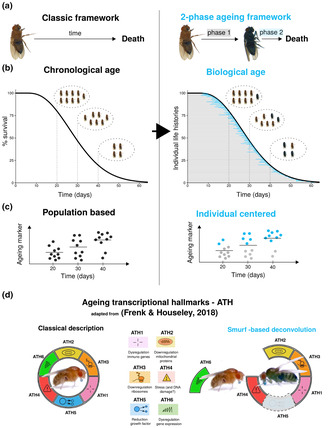

**Abbreviations**
ATH: ageing transcriptional hallmark
BP: biological processes
DEGs: differentially expressed genes
DGRP: Drosophila Genetic Reference Panel
ETC: electron transport chain
FC: fold change
FD&C: Food, Drugs, and Cosmetics
GFP: green fluorescent protein
GO: gene ontology
GSEA: gene set enrichment analysis
KD: knock down
KEGG: Kyoto Encyclopedia of Genes and Genomes
ML: mean lifespan
OX: over expression
PCA: principal component analysis
RNA: ribonucleic acid
RNA‐Seq: RNA sequencing
RSD: relative standard deviation
RU486: mifepristone
T50: median lifespan
TF: transcription factor
UPR: unfolded protein response

## 1 INTRODUCTION

### 1.1 Chronological age and physiological ageing

Ageing is commonly defined as a progressive decrease in functional efficiency associated with an age‐related increasing vulnerability to death (Lemoine, 2020; Lopez‐Otin et al., 2013), although different modalities can be found across the livings (Jones et al., 2014). In a given population, individuals of the same chronological age can yet experience different risks of mortality, showing that physiological ageing is not fully captured by chronological age. In humans, the notion of frailty—an unobserved individual modulator of the force of mortality—was introduced to explain this heterogeneity (Vaupel et al., 1979). It was followed by the definition of specific frailty indexes, fixed sets of characteristics that can be used to predict an individual's risk of death independently of its chronological age (de Vries et al., 2011; Dent et al., 2016; Fulop et al., 2010). On the one hand, the use of such frailty indexes has now been extended to several model organisms (Baumann et al., 2018; Heinze‐Milne et al., 2019; Whitehead et al., 2014). On the other hand, efforts to define ageing at the cellular and molecular levels have led to the definition ‘hallmarks of ageing’ (Lemoine, 2021; López‐Otín et al., 2013, 2023), evolutionary conserved molecular markers progressively affected in ageing individuals—and to the development of ageing clocks predicting biological age based on molecular markers. Ageing clocks based on 5‐cytosine methylation of CpG sites (Bocklandt et al., 2011; Hannum et al., 2013; Horvath, 2013; Horvath & Raj, 2018) work well in mammals but do not apply to model organisms such as *Caenorabditis elegans* or *Drosophilia melanogaster*. Nevertheless, recent work has identified a ‘universal’ transcriptomic clock using *C. elegans* (Tarkhov et al., 2019), with the recent publication of the BiT age clock (Meyer & Schumacher, 2021), suggesting a possible conservation of critical biological age markers.

### 1.2 The Smurf approach to ageing

The Smurf assay is an in vivo non‐invasive assessment of increased intestinal permeability (IP) based on co‐ingestion of the non‐toxic blue food dye FD&C #1 (approx. 800 Da). The dye, normally not absorbed by the digestive tract, spreads throughout the body in flies with altered IP, turning them blue (Rera et al., 2012), hence their name Smurfs. The Smurf assay was previously shown to be a powerful marker of biological age in *D. melanogaster* (Rera et al., 2012; Tricoire & Rera, 2015) as well as other model organisms (Dambroise et al., 2016). Maintaining a population on standard food containing the dye reveals that the proportion of Smurfs increases as a function of time (Rera et al., 2012) and that all flies undergo the Smurf transition prior to death (Rera et al., 2012; Tricoire & Rera, 2015). Furthermore, Smurf flies present a low remaining life expectancy (T_50_ estimated at ~2.04 days across different genetic backgrounds from the DGRP set Mackay et al., 2012) that appears independent of their chronological age at Smurf transition (Rera et al., 2012; Tricoire & Rera, 2015). In a given population at any given age, the Smurfs are the only individuals showing high mortality risk, low energy stores, low motility, high inflammation and reduced fertility, making this subpopulation a characteristic frail subpopulation. We demonstrated, thanks to a simple two‐phase mathematical model, that we are able to describe longevity curves using the age‐dependent linear increase (approximation) of the Smurf proportion and the constant force of mortality in Smurfs (Tricoire & Rera, 2015).

The above‐mentioned studies led us to hypothesise that markers classically considered as progressively and continuously changing during ageing (the hallmarks of ageing) might actually accompany the Smurf transition and exhibit a biphasic behaviour (two‐phase model of ageing (Clark et al., 2015; Tricoire & Rera, 2015). The age‐dependent increase in mortality at the population‐level should then be re‐interpreted as the increasing proportion of Smurfs in the population of individuals still alive (Tricoire & Rera, 2015). To test this hypothesis, we assessed the transcriptional changes occurring in flies as a function of both their Smurf status and chronological age. RNA‐Sequencing (RNA‐Seq) was performed on Smurf and non‐Smurf individuals of different chronological ages after total RNA extraction from the whole body of mated female flies. Samples were collected at 20, 30 and 40 days after eclosion, corresponding to approximately 90%, 50% and 10% survival in the used line (*Drs*‐GFP; Figures S1 and S2).

## 2 RESULTS

### 2.1 Smurfs have a stereotypical transcriptome

**FIGURE 1:**
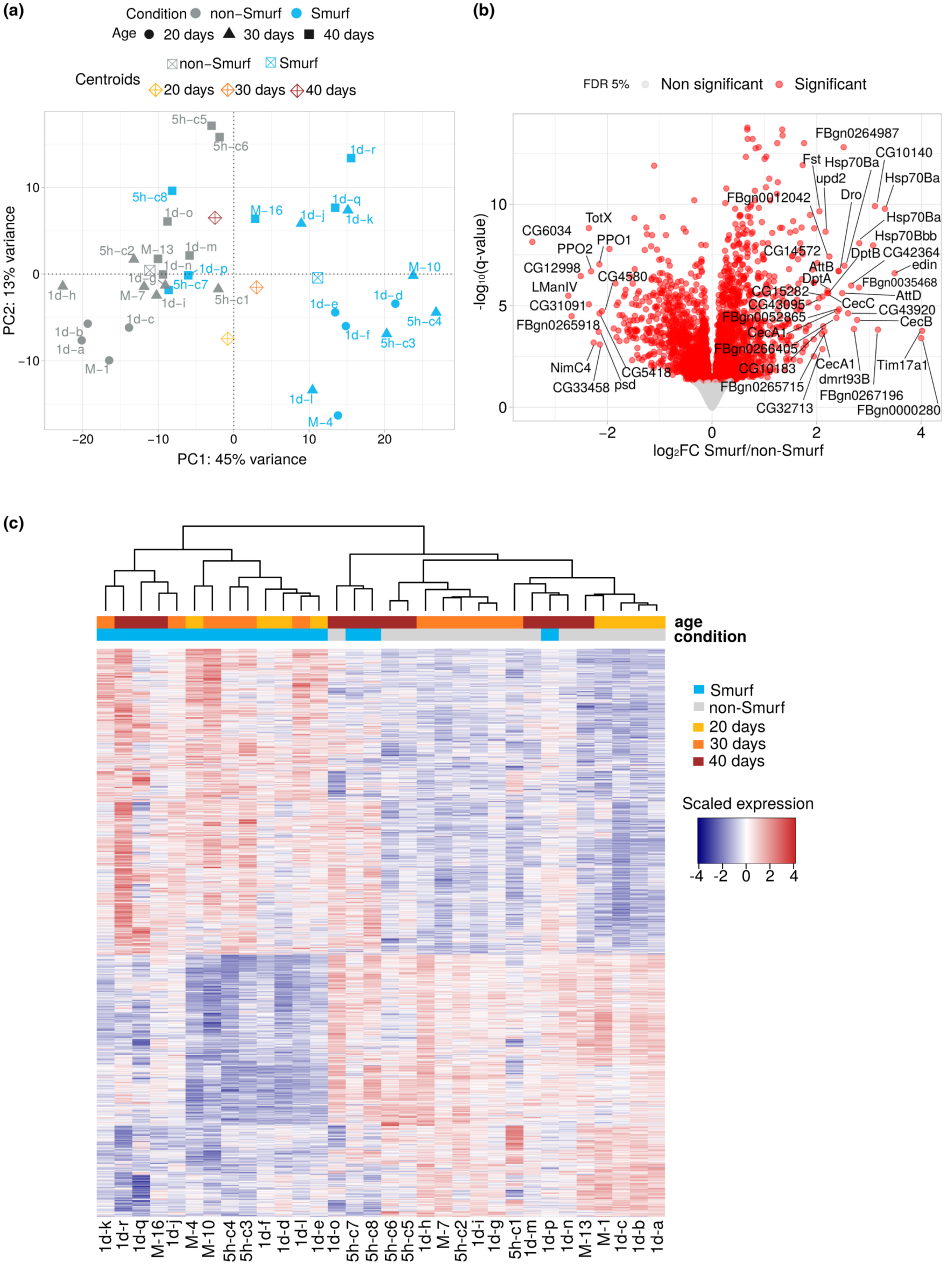
Smurfness is associated with a characteristic transcriptome. (a) Samples plotted in the space of the first two principal component analysis (PCA) components. PCA performed on the 1000 top‐variance genes results in a clear separation of Smurf (blue) and non‐Smurf (grey) samples on PC1 while samples are distributed according to age on PC2. This shows that Smurfness explains most of the transcriptome variance in our dataset (45% for PC1), followed by age (13% for PC2). Shapes indicate the age as illustrated in the legend. Centroids coordinates for a specific group are the mean of the group coordinates. Each sample is associated with an acronym specifying the collection time after the transition (5 h = 5 hours, 1d = 1 day and M = mixed—unknown time) and a unique letter or number identifying the sample itself. (b) Volcano plot of the differentially expressed gene (DEG) analysis results. The negative logarithm with base 10 of the False Discovery Rate (FDR) adjusted *p*‐value (*q*‐value) is plotted as a function of the shrinked (DESeq2 apeglm method; Zhu et al., 2019) fold change (base 2 logarithm) of the Smurf/non‐Smurf expression ratio for each gene. The significant 3009 DEGs are represented in red. Upregulated Smurf genes (1618) plot on the right side of the graph, while downregulated genes (1391) on the left. Genes with a log_2_FC > |2| are labelled. Amongst the genes annotated as upregulated we can notice the presence of immune response genes (*Dro*, *AttB*, *AttC*, *DptA*, *DptB*, *CecA1*, *CecB*, *CecC*), confirming what already described in Smurfs (Rera et al., 2012). (c) Smurf DEGs represent a Smurf‐specific signature. Unsupervised hierarchical clustering on the samples by Smurf DEGs only divides Smurfs from non‐Smurfs independently of their age, demonstrating that those genes are a Smurf‐specific signature. Non‐Smurf samples tend to cluster by age, suggesting an age trend in the expression of Smurf DEGs in non‐Smurf. The same three outliers of (a) are identified, indicating that those three samples indeed present a weaker expression pattern compared to the other Smurfs. Expression of genes in the heatmap is re‐centred on the mean across samples, for easy visualisation of upregulated and downregulated genes.

We first performed a principal component analysis (PCA) to explore how our multiple samples did relate to each other. The first component (45% of variance) separates Smurfs and non‐Smurfs samples (Figure 1a). This component is significantly associated with Smurfness (*R* ^2^) ANOVA = 0.604, *p* = 1.67e^−07^), but not with age (*p* > 0.05). The second component (13%) segregates samples as a function of chronological age (Pearson *ϱ* = 0.717, *p* = 3.92e^−06^), with no significant association with Smurfness (*p* > 0.05). The fact that three 40 days Smurfs samples out of six clusters with same age non‐Smurfs, a pattern confirmed using independent tSNE (t‐distributed stochastic neighbour embedding) and hierarchical clustering on sample‐to‐sample distance (Figures S3 and S4), indicates fewer differences between the transcriptomes of old Smurfs and non‐Smurfs than between young ones.

We proceeded to quantify the differences between Smurfs and non‐Smurfs through differential gene expression analysis (DESeq2; Love et al., 2014). Comparing the 16 Smurf and the 16 non‐Smurfs samples, we identified 3009 differentially expressed genes (DEGs) (Figure 1b, DESeq2 results in File S1). Confirming the PCA results, these genes represent a Smurf‐specific signature that clusters the Smurfs samples (Figure 1c). Again, the effect of chronological age is less marked in Smurf samples than in non‐Smurf ones. DESeq2 results were validated using the edgeR (Robinson et al., 2010) pipeline, which identified 2609 DEGs, 90% of which are overlapping with the DESeq2 output and present a strong correlation (Pearson *ϱ* = 0.99) for log_2_FC estimation (Figure S5).

### 2.2 Smurfness recapitulates the transcriptional signature of ageing

**FIGURE 2:**
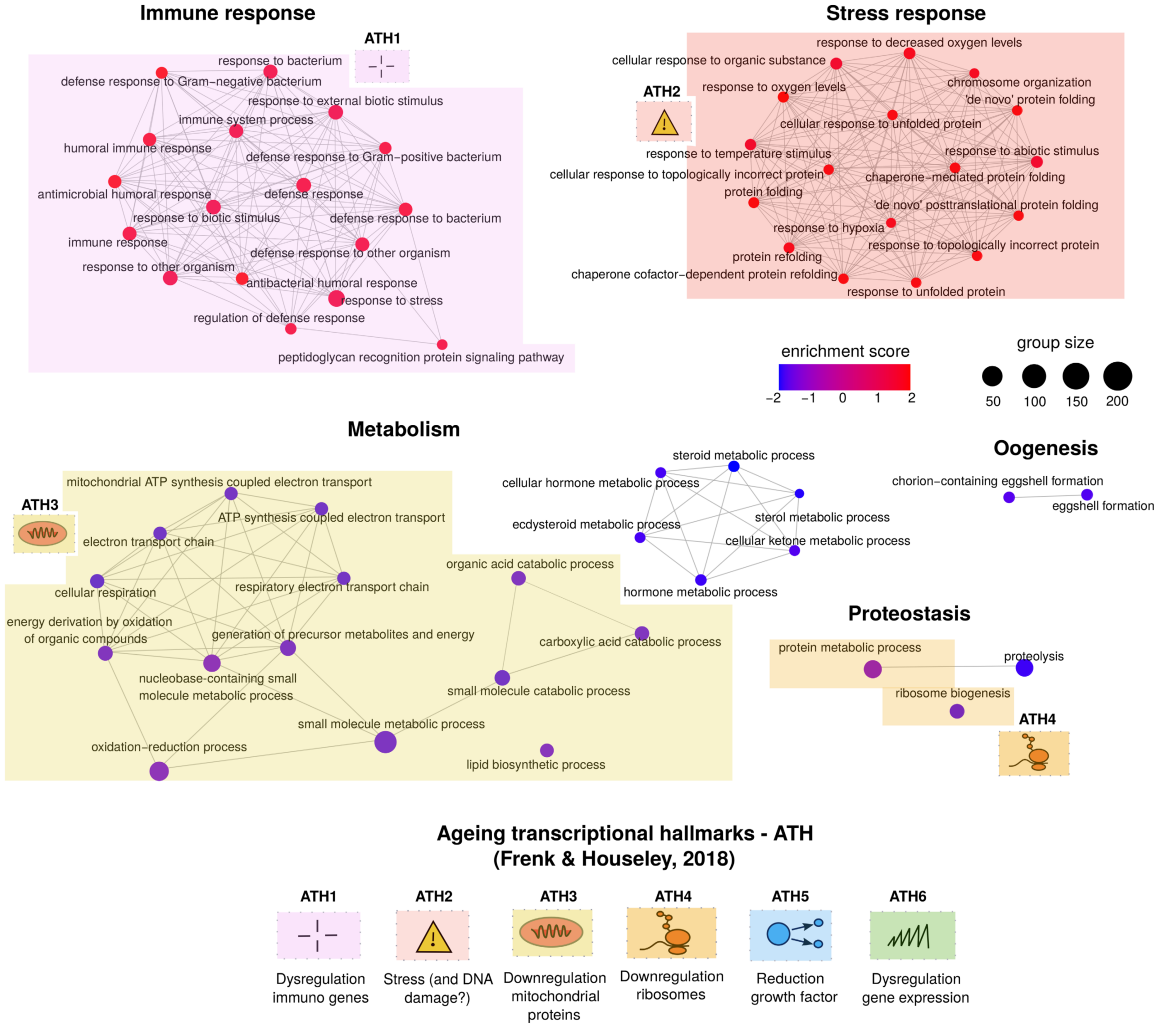
Gene Set Enrichment Analysis (GSEA) analysis (Gene ontology biological process categories) of Smurf‐specific genes. GSEA results are represented as a network, where nodes are significantly enriched categories (deregulation colour code as in legend) and edges are connected categories with overlapping genes. From the 59 significant categories, we identified and manually annotated five hubs: immune response, stress response, metabolism, proteostasis and oogenesis. Hallmarks of transcriptional ageing, as enunciated in (Frenk & Houseley, 2018) (bottom of figure). The hallmarks present in the Smurf‐specific signature (ATH1‐4) are mapped close to the related categories. Overall, in the Smurfs specific genes we detect four hallmarks of transcriptional ageing. Note that the DNA damage response (ATH4) is indicated with a question mark in the figure following the conflicting data presented by Frenk & Houseley. No category maps to ATH5 (reduction in growth factor, downregulation of cell cycle genes) and ATH6 (increased transcriptional hetereogeneity, DNA and RNA dysregulation).

We used biological processes (BP) Gene ontology (GO) (Ashburner et al., 2000) as gene sets in Gene Set Enrichment Analysis (GSEA) (Subramanian et al., 2005) to characterise the Smurf signature. In order to fully examine the observed signal, we chose not to apply any filtering on the log_2_FC (FC: fold change). We mapped our results on the hallmarks of transcriptional ageing (ATH 1–6) described in Frenk and Houseley (2018) on the GSEA network (Figure 2 and Table S1). Genes upregulated in Smurfs are enriched in immune and stress response (ATH1), as previously reported in Smurfs (Rera et al., 2012) as well as numerous ageing transcriptomic studies in Drosophila (Bordet et al., 2021; Girardot et al., 2006; Landis et al., 2004; Moskalev et al., 2019; Pletcher et al., 2002; Zhan et al., 2007) and other organisms (Benayoun et al., 2019; de Magalhães et al., 2009; Kazakevych et al., 2019; Lee et al., 2000; Palmer et al., 2021; Wang et al., 2022) including humans (Furman et al., 2017). Here, the immune response is widely upregulated, with activation of both Toll (fungi and Gram‐positive response) (Lemaitre et al., 1996) and Immune deficiency (Imd, Gram‐negative response) (Dushay et al., 1996; Lemaitre et al., 1995) pathways. Antimicrobial peptides (AMPs), which are surrogates of inflammation in flies, are strongly upregulated (*CecA1*, *CecA2*, *CecB*, *CecC*, *DptA*, *Def*, *Dpt*, *Drs*, average log_2_FC = 2.33) with their upstream regulators *Rel* (Imd pathway, log_2_FC = 0.61) and *dl* (Imd pathway, log_2_FC = 0.27) also upregulated.

Stress responses (ATH2) such as protein folding and unfolded protein response (UPR, with upregulation of *Xbp1* and *Ire1*) are over represented in our dataset. Smurfs present a significant induction of 22% of Drosophila chaperons and co‐chaperons (Flybase; Larkin et al., 2021 annotation, version FB2022_04), with a broad upregulation of the Hsp70 family (6 out of 7 genes detected are upregulated, average log_2_FC = 2.60), as previously described in ageing (Landis et al., 2004; Yang & Tower, 2009). We detect a significant upregulation of 51% of the annotated cytosolic Glutathione S‐transferases (Gst), a family of genes involved in detoxification and oxidative stress response.

Downregulated genes show a broad enrichment in metabolism‐related categories (ATH3). The decreased expression of genes involved in fatty acid biosynthesis, such as *FASN1* (log_2_FC = −0.61), *ACC* (log_2_FC = −0.31) and *eloF* (log_2_FC = −0.41) corroborates the decreased triglycerides content previously described in Smurfs (Rera et al., 2012). The mitochondrial electron transport chain (ETC) also shows a broad downregulation (ATH3). In order to provide a quantification of the ETC downregulation, we mapped the Smurf DEGs on ETC complexes Flybase annotation, and computed the percentage of downregulated genes. Through all the complexes, all the genes detected as DEGs are downregulated (no upregulation observed) (Complex I: 17 genes, 38% of the Complex I, average log2FC = −0.18; Complex II: 2 genes, 33% percent of Complex II, average log2FC = −0.17; Complex III: 4 genes, 29% of the Complex III, average log2FC = −0.21; Complex IV: 4 genes, 19% of Complex IV, average log2FC = −0.18; Complex V: 7 genes, 41% of Complex V, average log2FC = −0.19. Percentage refers to the number of genes detected in our dataset for the specific complex). Despite the minor fold changes, the ETC components' persistent downregulation may indicate that the aerobic metabolism they mediate is also downregulated. In addition, the upregulation of lactate dehydrogenase gene (*Ldh*) (log_2_FC = 0.95) could suggest a compensatory anaerobic metabolism replacing a probable dysfunction of the aerobic ETC path, or an altered pyruvate intake into the mitochondria. Consistently, *Idh3A*, *Idh3B*, *Mdh1*, *Mdh2* and *Fum1*, involved in the tricarboxylic acid (TCA) cycle are downregulated, with fold changes similar to the ones reported above.

Genes involved in ecdysone biosynthesis (*sad*, *spo* and *phm*) and egg formation (*Vm26Aa*, *Vm26Ab*, *Vml* and *psd* are downregulated (log_2_FC is, respectively, −2.67, −2.63, −2.51, −2.49), giving a molecular hint for explaining the previously reported decrease in fertility in Smurf females and males (Rera et al., 2018). A few categories related to proteostasis are also present amongst the ones deregulated in Smurfs. The ribosome biogenesis category (GO:0042254), mapping to ATH4, contains 190 genes out of which 46 are significantly deregulated, most of them, 96%, being downregulated. Regarding the proteolysis category, we detected the downregulation of 10 trypsin‐like endopeptidases and 14 Jonah genes (serine endopeptidases family).

The Smurf signal overlaps with numerous changes that were described so far as ageing‐related, mapping to four out of six ATH (ATH 1–4).

We compared our results with proteomic and metabolomic data obtained from Smurf and non‐Smurf mated females from the same genetic background. Enrichment analysis on significantly differentially represented proteins (ANOVA *p* < 0.05, for complete results see File S2) confirms our results of a decreased fatty acid catabolism, mitochondrial respiration and ribosomal proteins (Figure S6). Response to stress (including genes such as *cact*, *Hsp70* and *Cat*) is upregulated, in line with what described in our transcriptome study.

Quantitative enrichment analysis on metabolites concentrations in Smurfs and non‐Smurfs (File S3) confirms the molecular separation between the two phases (Figure S7) and the metabolic transcriptional signature observed. We detected deregulation of fatty acid biosynthesis and degradation pathways (Kyoto Encyclopedia of Genes and Genomes [KEGG]; Kanehisa & Goto, 2000), with palmitic acid [log_2_FC = −1.37] and myristic acid [log_2_FC = −1.69], Figure S8) and pyruvate metabolism (which includes metabolites from the TCA cycle) (Table S2). Regarding glucose metabolism, the overexpression (OX) of *Ldh* is confirmed by a significant (Wilcoxon test, *p* < 0.05) lactic acid increase in Smurfs (log_2_FC = 0.90) (Figure S9). The TCA cycle displays a significant general decrease at a transcriptomic level, and a general impairment at a metabolomic level, though the only metabolite significant to Wilcoxon test is succinate, (log_2_FC = 1.28) (Figure S10).

These results indicate that the transcriptional dysregulation observed in Smurfs has a functional impact.

### 2.3 Old Smurfs carry additional age‐related changes

**FIGURE 3:**
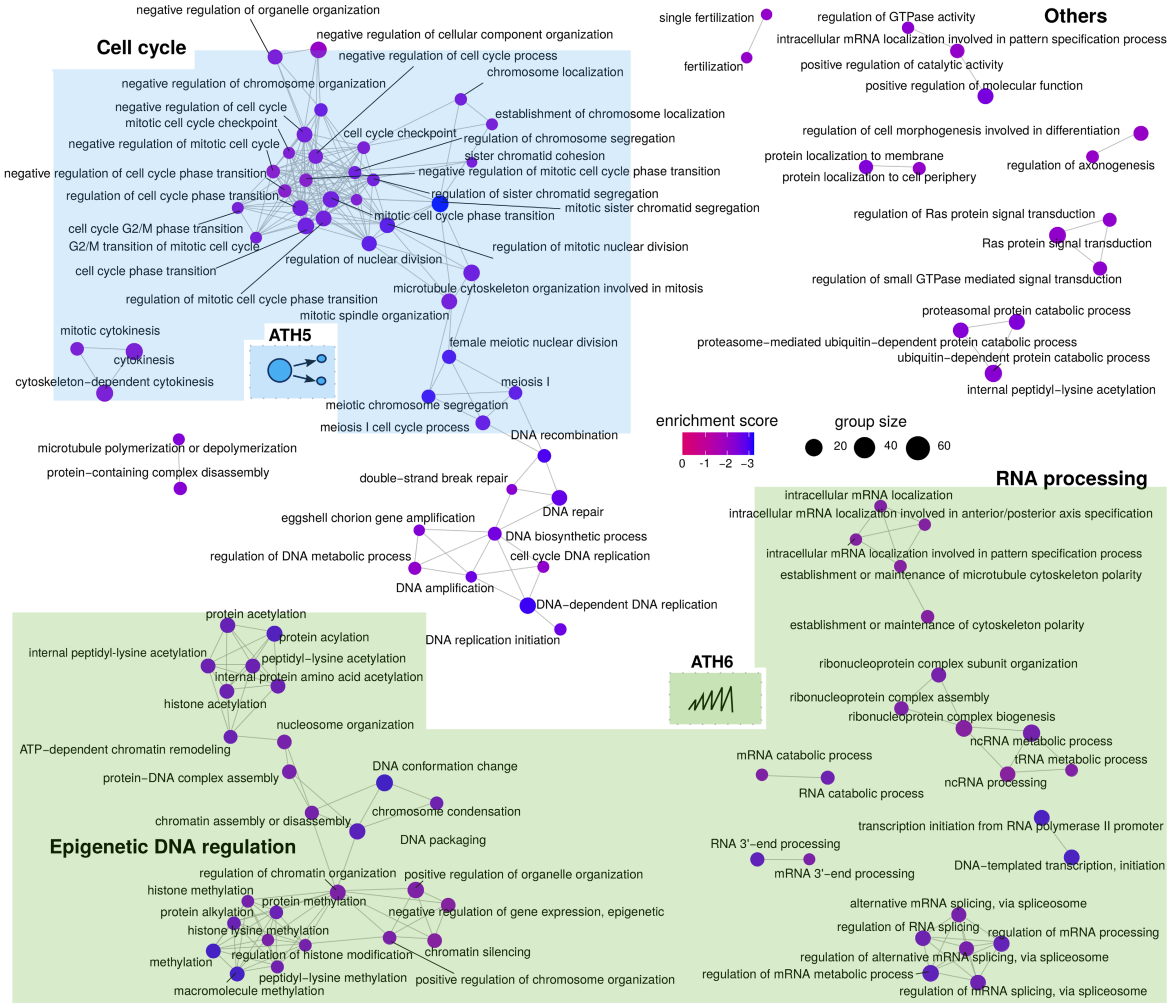
Old Smurfs carry an ageing‐related signal amongst downregulated genes. Results of the Gene Set Enrichment Analysis (GSEA) analysis are represented as in figure. Only downregulated nodes presenting at least one interconnection are represented here. Complete list of deregulated categories can be found in Table S8. GSEA analysis identifies 115 downregulated Gene ontology biological processes categories, which are mostly related to DNA regulation, RNA processing and cell cycle regulation. A few nodes are associated with DNA repair. Interestingly, the signal carried by the old Smurfs maps (at least partially) to the ‘dysregulation in gene expression’ (in green, ATH6) and the ‘reduction in growth factors’ (ATH5) transcriptional ageing markers that were not detected in the Smurf‐specific signature. In addition, the DNA damage nodes show downregulation of genes involved in DNA repair, which has also been discussed as an ageing marker. Interestingly, there are no hubs in the network overlapping with the Smurf‐specific signature of Figure 2, showing that the core Smurf signal is not affected by chronological age. However, the old Smurfs do carry an additional signature compared to their younger counterparts, suggesting the existence of a ‘chronological‐age burden’ that might increase the probability of entering the Smurf pre‐death phase, without however being necessary or sufficient for it.

Our analysis (Figure 1a, Figures S3 and S4) suggested transcriptional differences between the old and young Smurfs. We therefore applied a DEG analysis restricted to Smurfs. Only 4 DEGs were identified when comparing 20 and 30‐day Smurfs (FDR cut‐off at 5%) while the 40 days Smurfs present 2320 DEGs compared to 20‐day Smurfs (1385 upregulated and 935 downregulated) (DESeq2 results in File S4). GSEA identified 125 deregulated GO BP categories (Figure 3 and Table S3). The majority of the detected categories are associated with RNA processing, transcription, chromatin organisation, DNA replication and repair (ATH6). In the case of old Smurfs, we find downregulation of genes involved in histone methylation (*trr*, *Cfp1*, *Dpy‐30L1*, *Smyd5*, *NSD*, *CoRest*, *Lpt*, average log_2_FC ~ −0.26), amongst which genes of the Polycomb Repressive Complex 2 (*esc*, *E*(*z*), *Su*(*z*)*12I*, average log_2_FC ~ −0.24). We also detect the downregulation of the histone deacetylase *HDAC1* (log_2_FC = −0.18) and genes involved in histone acetylation (as *CG12316*, *Ing3*, *Ing5*, *Taf1*, *Atac3*, *Brd8*, *Spt20*, *mof*, average log_2_FC ~−0.30). Chromatin‐related genes are thus modestly (0 < |log_2_FC| < 1) but broadly decreased in old Smurfs. Interestingly, our proteome analysis shows a significant decrease of H3.3B (log_2_FC = −0.43) and H4 (log_2_FC = −0.54) in Smurfs suggesting a ‘loss of heterochromatin’ (Villeponteau, 1997). Another interesting signal is the DNA repair nodes (‘GO:0006302 double‐strand break repair’, ‘GO:0006281 DNA repair’), where we retrieve 12% of the detected genes as significantly downregulated (average log_2_FC = −0.24). We also retrieved nodes associated with downregulation of genes involved in cell cycle (as cyclins), or their regulators (as *E2f2*, log_2_FC ~ −0.17), which map to the ATH5 (growth factor and regulation of cell cycle). Genes involved in spindle organisation during mitosis are also found downregulated (as *Mtor*—log_2_FC ~ −0.28‐ and *Chro*—log_2_FC ~ −0.19‐) suggesting a broad dysregulation of cell proliferation processes.

The old Smurf signature therefore partially carries ATH5 and ATH6, the two hallmarks of transcriptional ageing that we did not detect in the Smurf‐specific signature. It is important to highlight that we do not find Smurf‐related categories in the GSEA output, confirming that young Smurf and old Smurfs indeed do carry the same Smurf signature illustrated in Figure 2. However, our analysis shows that the old Smurfs carry additional transcriptional changes, which mostly relate to transcription and DNA regulation. To investigate whether those are time‐dependent changes, which are weakly carried by old individuals and then enhanced in the Smurf stage of their life, we fitted a per‐gene regression model on all samples, including as explanatory variables Smurfness, time and an interaction term amongst the two. We then performed GSEA on the list of genes presenting significant coefficients (*F* statistic, list of significant coefficients in File S5). The RNA processing categories (as well as the ‘chromosome organization’) were detected as significantly affected by time, suggesting that the deregulation trends for such processes may already be present in the non‐Smurfs.

### 2.4 Removing the Smurf‐specific signature unveils the transcriptional effects of chronological age

**FIGURE 4:**
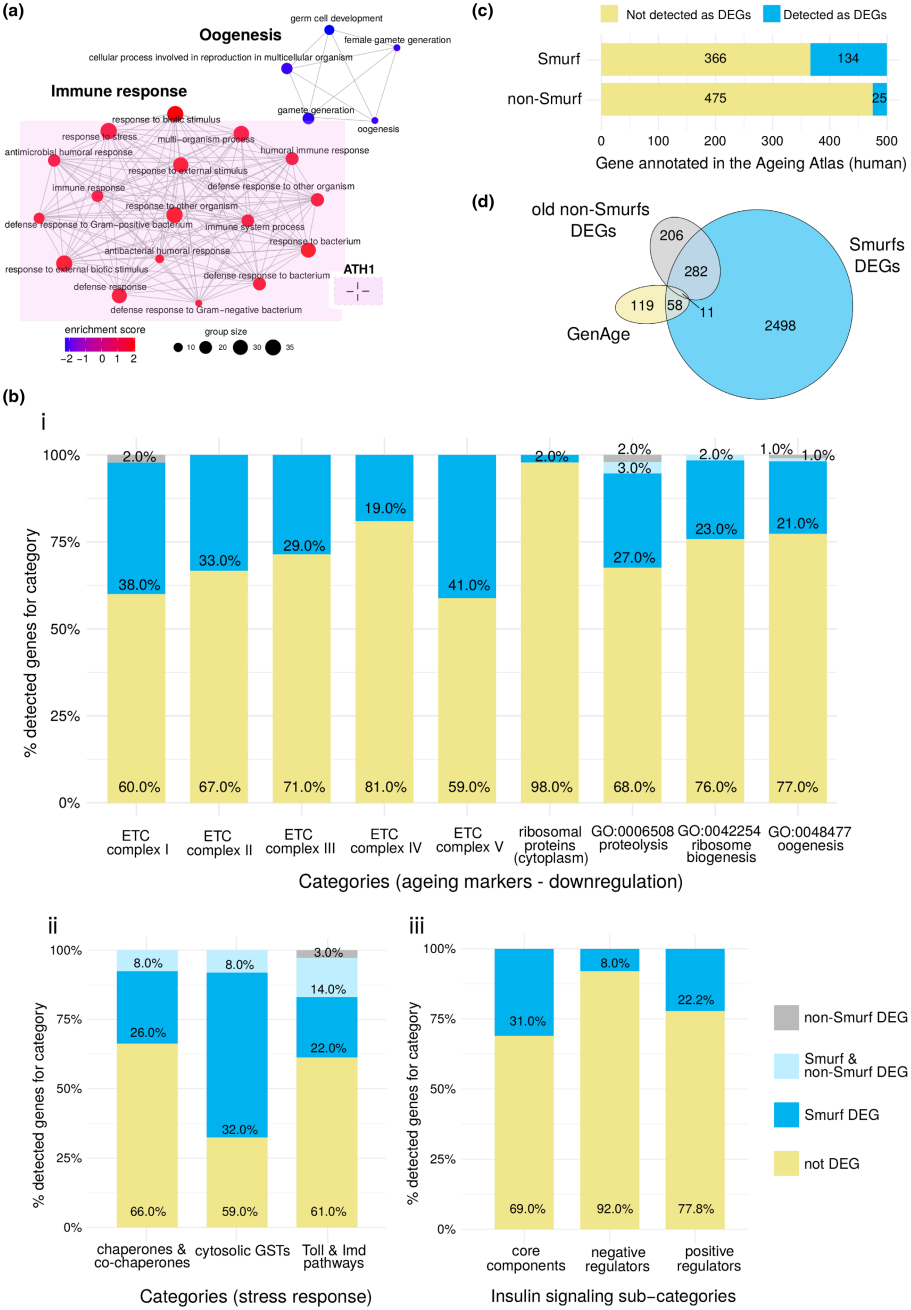
Smurfness is a better predictor of transcriptional ageing markers than chronological age. (a) Gene Set Enrichment Analysis (GSEA) analysis (Gene ontology (GO) biological processes (BP) categories) on old non‐Smurf‐specific genes. Results are represented as in Figure 2. GSEA analysis identifies 22 deregulated GO BP categories, related to immune response (upregulation, in red) and oogenesis (downregulation in blue). The analysis carried on chronological age can therefore detect only one hallmark of transcriptional ageing (Frenk & Houseley, 2018) (ATH1, for representation of transcriptional hallmarks, see Figure 2). (b) Manual mapping of Smurf and old non‐Smurf differentially expressed genes (DEGs) on ageing processes. For each process, the histograms represent the percentage of genes mapping to it but not detected as DEGs in our analysis (yellow), detected as Smurf DEGs (blue), detected as both Smurf and non‐Smurf DEGs (light blue) or only detected in the old non‐Smurf DEGs (grey). When not stated otherwise, the gene lists are retrieved from Flybase. Genes described as downregulated with ageing (i) are mostly detected only in Smurfs, with the exception of structural ribosomal proteins, whose downregulation is not significant in Smurfs. For the processes described as upregulated with ageing (ii), the Smurf samples do retrieve more information than the non‐Smurfs, with the last however carrying more signal than in the case of the downregulated genes, especially for the immune response; as already showed in (a). Similarly, the IIS pathway displays deregulation in the Smurfs, while no gene is detected as deregulated when looking only at chronological age (iii). (c) Mapping of Smurf and non‐Smurf DEGs to human ageing‐related genes (annotated in the Ageing Atlas). The Ageing Atlas annotates 500 human ageing‐related genes. All of those have orthologs in Drosophila, which are all present in our dataset. By studying the Smurf phenotype, we can detect 134 genes out of the annotated 500. The number of detected genes drops to 25 when using chronological age only as an ageing marker. (d) Longevity genes and Smurfness. The Venn diagram shows the overlap between the annotated longevity genes in Drosophila (GenAge), the Smurf DEGs and the non‐Smurf DEGs. While Smurf‐centred analysis retrieves ~37% of the longevity genes, the non‐Smurf centred analysis only retrieves ~6%, not adding information to what was already detected by the Smurf analysis.

**FIGURE 5:**
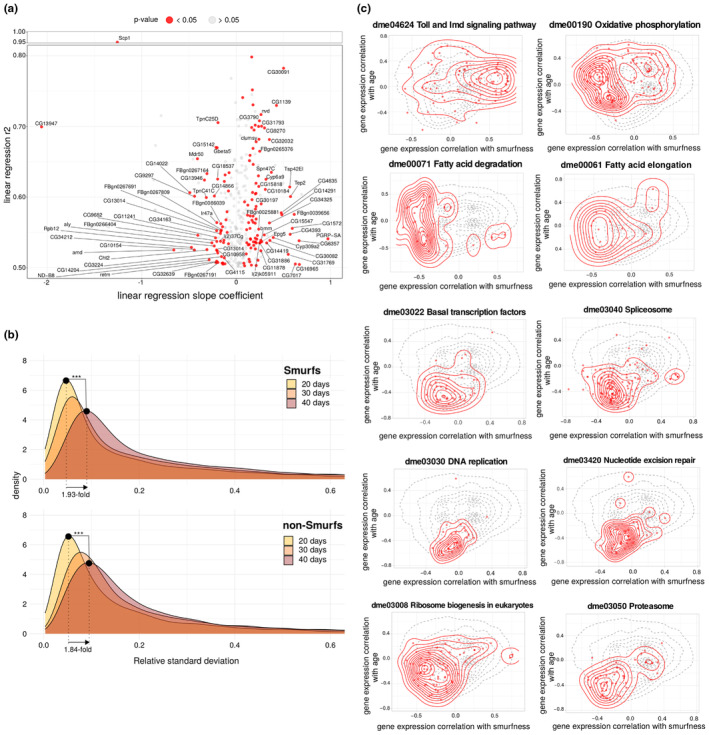
Chronological age and Smurfness respective effects on the transcriptome. (a) Linear regression of gene expression in non‐Smurfs over time. The *r* ^2^ of the applied linear model is plotted as a function of the slope coefficient. Only genes non differentially expressed in Smurfs are plotted, in order to focus on a possible weak age‐related non‐Smurf signal. Genes presenting a significant slope are plotted in red. (b) Chronological age effect on transcriptional heterogeneity. The relative standard deviation (RSD) densities are plotted for the different ages group (Smurf and non‐Smurf). The tail of the distribution is cut at RSD = 0.6 for illustration purposes. Smurfs and non‐Smurfs present a similar behaviour, with the peak of the distribution showing an almost 2‐fold increase from 20 to 40 days (peak_S20_ = 0.046, peak_S40_ = 0.089, peak_NS20_ = 0.051, peak_NS40_ = 0.094), showing the effect of chronological age on transcriptional noise. ***p < 10^−16^ (KS statistic). (c) Effect of Smurfness and chronological age on biological pathways. Smurfness and chronological age both affect the biology of the individual. Here, we show how some pathways are affected by age and Smurfness, respectively. Dotted line in the background corresponds to the density of all the genes analysed. Red points and density correspond to the genes mapping to the pathway (Kyoto Encyclopedia of Genes and Genomes database) of interest. The statistics was assessed using the Fasano‐Franceschini test (FDR adjusted *p*‐value). Toll and Imd pathways (*r*_smurf_ = 0.248, *r*_age_ = 0.080, *p* = 5.2e^−06^), oxidative phosphorylation (ETC genes, *r*_smurf_ = −0.217, *r*_age_ = 0.088, *p* = 4.5e^−15^), fatty acid degradation (*r*_smurf_ = −0.388, *r*_age_ = −0.063, *p* = 4.3e^−09^) and fatty acid elongation (*r*_smurf_ = −0.255, *r*_age_ = −0.031, *p* = 3.8e^−03^) are mostly correlating with smurfness; spliceosome (*r*_smurf_ = − 0.124, *r*_age_ = −0.288, *p* = 1.5e^−17^), basal TFs (*r*_smurf_ = −0.096, *r*_age_ = −0.318, *p* = 3.1e^−08^), DNA replication (*r*_smurf_ = −0.070, *r*_age_ = −0.393, *p* = 2.2e^−09^) and repair (Nucleotide excision repair, *r*_smurf_ = −0.073, *r*_age_ = −0.338, *p* = 1.2e^−10^) are mostly correlating with age; ribosome biogenesis (*r*_smurf_ = −0.203, *r*_age_ = −0.159, *p* = 4.0e^−10^) and proteasome (*r*_smurf_ = −0.166, *r*_age_ = −0.276, *p* = 3.5e^−09^) appear to occupy a zone of similar correlation with both Smurfness and age (with the peak of the density for the ribosomal pathway occupying a zone of high correlation with Smurfness, as expected given the results obtain in our analysis ‐Figures 2 and 3).

In order to confirm the Smurf‐specificity of the signature described above, we removed Smurf samples from the study and compared the non‐Smurfs over time. Only 526 DEGs were found when comparing 20‐ and 40‐days‐old non‐Smurfs (and 57 when comparing 20 and 30 days old non‐Smurfs) (DESeq2 results in File S6). 59% of these genes are overlapping with Smurf‐specific DEGs. Twenty‐two GO BP deregulated categories were found by GSEA (Figure 4a and Table S4). Overall, the genes that are known as being downregulated with ageing are actually downregulated mostly in Smurfs (Figure 5b, point i), with little to no effect associated with chronological age (Figure 4b, point ii). The largest overlap is observed for the immune response pathways (ATH1, increased inflammation). Out of the overlapping genes (20), 50% are AMPs, produced downstream the pathway. We do not find significant deregulation of the *dl* transcription factor (TF) (Smurf significant log_2_FC = 0.27), while *rel* is upregulated (log_2_FC = 0.42, while for the Smurfs we detected a log_2_FC of 0.61). These results suggest that the immune response is active in the old non‐Smurf but to a lower extent than in Smurfs.

Regarding the genes mapping to the insulin‐like receptor signalling (IIS) pathway (Figure 5b, point iii), we do not detect any deregulation in the non‐Smurfs, with IIS core components being affected only in Smurfs. While no significant change is detected for the *Ilp* genes (insulin‐like peptides activating the pathway), we find low but significant upregulation of *Inr* (receptor, log_2_FC = 0.42), *chico* (first kinase of the cascade, log_2_FC 0.23) and the kinase *Akt1* (log_2_FC = 0.18). *Inr* and *chico* are well‐described longevity genes in Drosophila, positively affecting ageing when negatively modulated (Clancy et al., 2001; Tatar et al., 2001). No significant changes are detected for the Drosophila mTOR genes *Tor* and *raptor*, nor *foxo*. However, we find significant upregulation of *Thor*, coding for the homologous mammalian translation initiation factor 4E‐BP, a *foxo* target of which the upregulation was already described at the protein level in Smurfs (Rera et al., 2012).

Our dataset contains all the orthologs of the 500 human genes associated with ageing present in the Ageing Atlas (Aging Atlas Consortium, 2021) (Tables S5 and S6). We find that 26.8% of these genes are present in the Smurf list (121 Drosophila genes corresponding to 134 human genes), while only 4% are present in the old non‐Smurfs (24 Drosophila genes corresponding to 25 human genes) (Figure 4c).

Over the past 40 years, numerous genes have been shown to modulate ageing when artificially deregulated. We explored whether our list of DEGs is overlapping these ‘longevity genes’. Out of the 201 Drosophila longevity genes annotated in GenAge (Tacutu et al., 2018), 188 are present in our dataset. Smurfs DEGs allow the detection of 37% of them, while the old non‐Smurf DEGs detect only 6% (Figure 4d and Tables S7 and S8). Furthermore, all the longevity genes present in the non‐Smurf DEGs are also present in the Smurf DEGs.

Taken together, the results show that Smurfness predicts ageing‐associated changes described in the literature better than chronological age.

### 2.5 Identifying weak chronological age‐dependent signature

In light of the evidence that most of the transcriptional alterations described as age‐related are Smurf‐specific, with only a small part of the signal retrieved in old non‐Smurfs (Figure 4), we wondered whether weaker but relevant age‐related changes might be present in non‐Smurfs but missed by the DESeq2 approach. We therefore regressed gene expression data on chronological age (20, 30, 40 days) in the non‐Smurfs using a linear model. After filtering for significance to *F*‐test (*p* < 0.05) and *R* ^2^ (>0.5), we identified 301 genes (207 showing an increasing expression with time, 94 decreasing) (Table S9). 51.6% of these genes also belong to the Smurf DEGs. We focused on the 146 remaining genes (93 with positive slope and 53 negative). Results are presented in Figure 5a. No enrichment in GO categories was found (GOrilla enrichment; Eden et al., 2009, using the whole set of detected genes as background), suggesting that once the Smurf signal is removed, no strong coherent deregulation can be detected in the non‐Smurfs in our dataset. Nevertheless, Figure 2a shows the old non‐Smurf samples to cluster with old Smurf samples. This is supported by the decreasing number of detected DEGs between age‐matched Smurf and non‐Smurfs with chronological age (Figure S11).

Ageing has been reported as increasing the gene expression heterogeneity in a variety of organisms, tissues and cell types (Bahar et al., 2006; Brinkmeyer‐Langford et al., 2016; Enge et al., 2017; Işıldak et al., 2020; Kedlian et al., 2019; Martinez‐Jimenez et al., 2017; Perez‐Gomez et al., 2020; Somel et al., 2006) (ATH6). We computed the relative standard deviation (RSD) of each gene for each group (Smurfness and age), plotted the distributions of the RSD across groups and compared them using the Kolmogrov–Smirnov (KS) statistic (Figure 5b). All genes are affected, independently of their expression levels (Figure S12). In both Smurfs and non‐Smurfs, the peak of the RSD distribution shifts towards the right with age (1.93‐fold increase for the Smurfs, and 1.84‐fold for the non‐Smurfs) suggesting that gene expression increases in heterogeneity as a function of chronological age with milder changes at the Smurf transition.

In brief, our results show that four out of six transcriptional ageing markers (ATH1‐4) are specific to the Smurf phenotype, independently of their chronological age (Figure 2). On the other hand, the alteration in chromatin‐related genes and mRNA processing, as well as cell cycle genes (together with a weaker DNA repair signal) appear to be exclusively carried by the old Smurfs (ATH 5–6) (Figure 3). We could not identify BP strictly related to the old non‐Smurfs compared to their young counterparts (Figure 4). However, the increased heterogeneity in gene expression (ATH6) appears to be primarily affected by chronological age (Figure 5b). In order to visually represent the relative effect of both the chronological and biological age, we computed the correlation of individual gene expression with each. We identified 113 annotated KEGG pathways where at least 10 genes present in our dataset are mapped. We finally obtained 48 correlating (Fasano‐Franceschini test (Fasano & Franceschini, 1987), FDR for *p*‐value correction) with Smurfness (Table S10) and 38 correlating with chronological age (Table S11). Figure 5c shows the Toll and Imd pathways mostly displaying positive correlation with Smurfness; the ETC (oxidative phosphorylation pathway) and fatty acid degradation/elongation mostly negatively correlates with Smurfness, while showing a lower correlation with age. Interestingly, transcription‐related pathways (spliceosome and basal TF) as well as DNA amplification and repair pathways show a higher negative correlation to chronological age compared to Smurfness. Finally, the proteasome and ribosome biogenesis seem equally affected by chronological age and Smurfness.

### 2.6 Using Smurfness to identify new ‘longevity genes’

We decided to investigate whether altered expression of TFs could explain the transcriptional signature of Smurfs. We identified 102 TFs showing altered expression in Smurfs (77 upregulated, 25 downregulated, Table S12) out of the 629 annotated in Flybase. In order to reduce the potential functional redundancy in this list, we used i‐cisTartget (Herrmann et al., 2012; Imrichová et al., 2015) to predict putative upstream regulators of the Smurf‐deregulated TFs. We selected the hits presenting a score above 4 (3 being the recommended minimum threshold). Second, to avoid limiting our selection criteria only to TFs, we applied the same i‐cisTarget algorithm to genes showing at least a 4‐fold difference (|log_2_FC| > 2). Results are shown in Table S13. We selected 17 TFs of interest for functional validation amongst the best i‐cisTarget scores or high deregulation (Table 1).

**TABLE 1:** List of transcription factors (TFs) selected for experimental validation. 17 TFs were selected for functional validation: 8 were found in the i‐cisTarget analysis, 3 in both DESeq2 and i‐cisTarget analysis and 6 in the DESeq2 analysis alone, chosen for their strong deregulation.

Gene symbol	Selection method	Deregulation
*Adf1*	i‐cisTarget	Putative regulator TFs up in Smurf
*Aef1*	i‐cisTarget	Putative regulator TFs up in Smurf
*CG4360*	i‐cisTarget	Putative regulator TFs up in Smurf
*FoxP*	DESeq2 & i‐cisTarget	Up in Smurf & putative regulator TFs up in Smurf
*Hsf*	i‐cisTarget	Putative regulator genes up in Smurf
*Trl*	i‐cisTarget	Putative regulator TFs up in Smurf
*dmrt93B*	DESeq2	Up in Smurf
*Ets21C*	DESeq2	Up in Smurf
*Hey*	DESeq2	Up in Smurf
*kay*	DESeq2	Up in Smurf
*Mef2*	DESeq2 & i‐cisTarget	Up in Smurf & putative regulator TFs up in Smurf
*rib*	DESeq2	Up in Smurf
*Ets96B*	DESeq2	Down in Smurf
*GATAd*	i‐cisTarget	Putative regulator TFs down in Smurf
*GATAe*	i‐cisTarget	Putative regulator TFs down in Smurf
*NF‐YB*	DESeq2 & i‐cisTarget	Up in Smurf & putative regulator TFs up in Smurf
*srp*	i‐cisTarget	Putative regulator TFs down in Smurf

**FIGURE 6:**
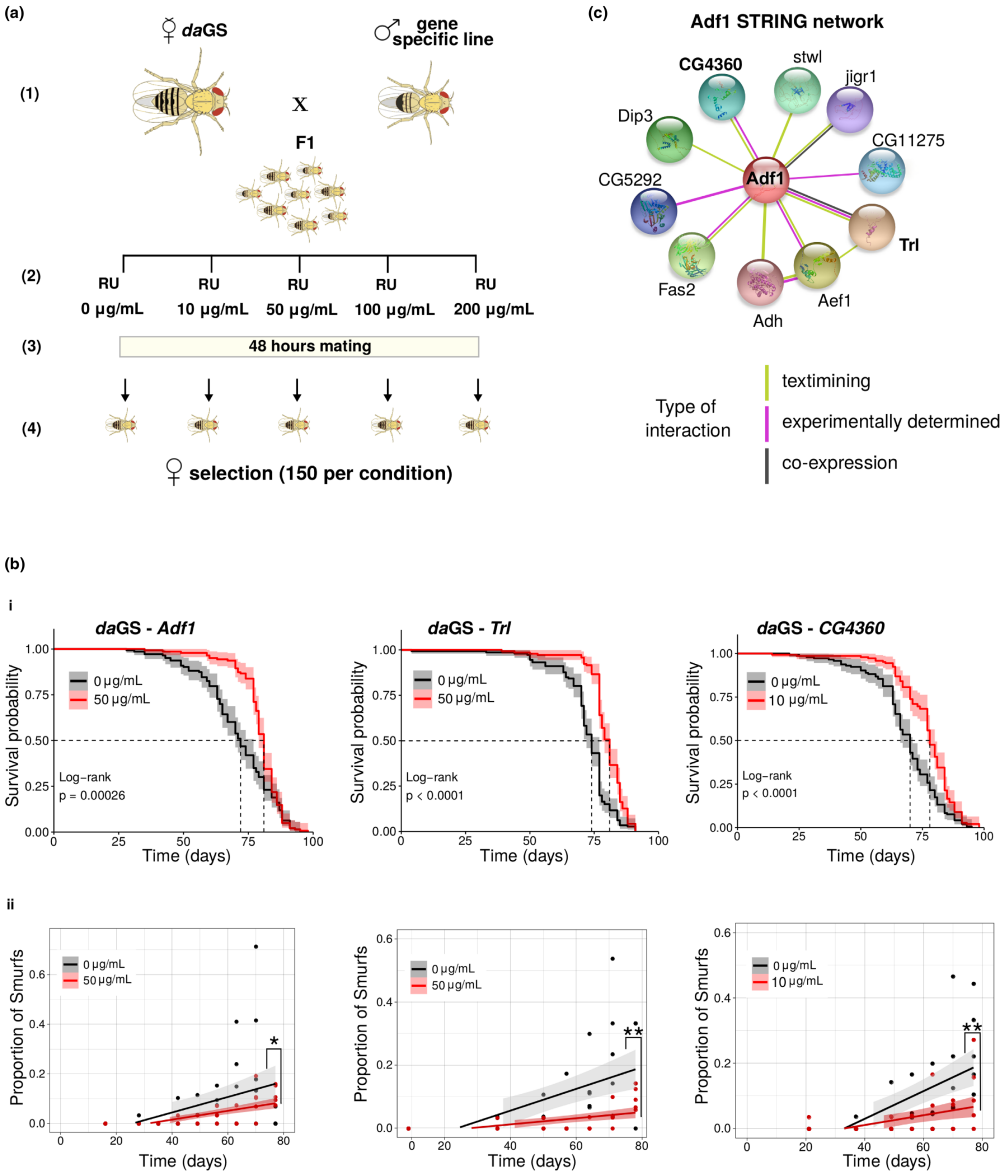
Identification of new longevity genes using the Smurf phenotype. (a) Gene expression alteration through GeneSwitch (GS). KD and/or overexpression of the target gene in the whole body of Drosophila were performed by crossing virgins females of the ubiquitous *daughterless*‐GS (*da*GS) driver with males carrying the UAS transgene (Step 1). The F1 was reared either on food without the inducer RU486 (adult only induction), either with food presenting the following RU486 gradient: 0 μg/mL ‐control‐, 1, 5, 10, 20 μg/mL (whole life induction). At the moment of eclosion, flies are transferred onto food with the following RU486 concentrations: 0 μg/mL ‐control‐, 10, 50, 100, 200 μg/mL. Flies are randomly distributed if not developed on drug, otherwise they are distributed according to the developmental drug condition (Step 2). Flies are left mating for 48 h (Step 3), and subsequently, 150 females per concentration (divided on 5 vials/30 females each) are randomly selected for the longevity experiment (Step 4). (b) Effect of *Adf1*, *Trl* and *CG4360* KD on longevity and on the Smurf dynamics in the population. (i) The KD of *Adf1* (+11.8%, ML_RU0_ = 71.0, ML_RU50_ = 79.5) and *Trl* (+10.5%, ML_RU0_ = 72.2, ML_RU50_ = 79.8) in the whole body during adulthood significantly extend lifespan, as well as for the KD during the whole life of *CG4360* (+12.4%, ML_RU0_ = 68.5, ML_RU10_ = 77.0). (ii) The proportion of Smurfs for the corresponding control and treated populations are plotted as a function of time. The proportion of Smurfs is computed as the number of Smurfs over the total number of flies alive (Smurfs + non‐Smurfs). Data are fitted using a linear approximation (Rera et al., 2012; Tricoire & Rera, 2015). In all cases, the populations show a significant increase with time of the Smurf proportion (*F* statistic) (*Adf1*: slope_RU0_ = 0.0055, *p*_RU0_ = 4.72e^−03^, slope_RU50_ = 0.0018, *p*_RU50_ = 4.17e^−07^; *Trl*: slope_RU0_ = 0.0044, *p*_RU0_ = 6.53e^−04^, slope_RU50_ = 0.0009, *p*_RU50_ = 5.39e^−04^; *CG4360*: slope_RU0_ = 0.0042, *p*_RU0_ = 6.58e^−05^, slope_RU10_ = 0.0015, *p*_RU10_ = 6.51e^−03^). Furthermore, the slope of the control population is significantly different from the one of the treated (*F* statistic), which displays a slower increase in the Smurf proportion with time. *p* values indicated in figure: *<0.05; **<0.01. (c) Adf1 interaction network from STRING database. The three TFs identified as new longevity genes have been retrieved from i‐cisTarget as putative regulators of upregulated Smurf TFs. The annotated interactions in the STRING database show how those genes have been already described together. Adf1 and Trl displayed stronger evidence (text mining, co‐expression and proved interaction in Drosophila in vitro), while the evidence for CG4360 and Adf1 interaction comes from text mining and interaction shown between homologous in *Caenorabditis elegans*). We decided to assign to CG4360 the gene name of *Sag1* (Smurfness‐associated gene 1) given its potential involvement in the Smurf phase. Regarding the remaining nodes of the network, they show weaker evidence (see Figure S19). CG11275 and CG5292 have been shown to interact with Adf1 on two‐yeast hybrid assay on the FlyBI project (https://flybi.hms.harvard.edu/).

To assess their effect on mean lifespan (ML), we proceeded with their knock‐down (KD) and/or OX using GeneSwitch (Osterwalder et al., 2001; Roman et al., 2001) (GS). This technique, widely used in Drosophila, allows spatially and temporally tuned KD or OX in individuals of the same genetic background. Since our candidate genes were selected from whole body data, we used the ubiquitous daughterless‐GS (*da*GS) driver. When transgenic lines were available we performed both KD and OX during the adulthood of the fly (i.e. after eclosion) or during its whole life (development and adulthood) (Figure 6a). Five different concentrations of RU486 (0 μg/mL ‐control, 10, 50, 100, 200 μg/mL) were used to explore a broad range of inducing conditions, as in ref (Tricoire et al., 2009). During development, we lowered the concentrations by a factor 10 in order to avoid potential toxic effects, as suggested by Osterwalder et al. (2001) and performed in Rera et al. (2010).

The longevity experiments are summarised in Figure S13 and Table S14. Four TFs presented a positive effect on ML when knocked‐down in at least one RU486 condition during adulthood *Trl* + 9.5%, *Adf1* + 7.6%, *CG4360* + 7.3%, *Ets96B* + 6.6%) and one when overexpressed during adulthood and development (*Hsf* + 10.3%). A second independent experiment confirmed the effect of *Trl*, *Adf1*, *CG4360* (Figure 6b, point i). A third experiment validated the ML extension of CG4360 when downregulated during adulthood only (Figure S14), as the first two experiments showed contrasting results for the longevity effect when downregulation was performed during the whole life. We confirmed the knockdowns through reverse transcriptase– polymerase chain reaction (RT‐qPCR) for each line (Figure S15), and validated that RU486 alone has no effect on ML (Figure S16).

We then tested whether the identified ML extension was due to delayed entry in Smurf state (Figure 6b, point ii) by fitting two linear regression models. First, in order to test the effect of chronological age, we regressed the proportion of Smurfs on chronological age separately in both the controls and KD individuals. Secondly, in order to investigate the difference between the two populations, we regressed the proportion of Smurfs on chronological age and RU486 concentration (as a categorical variable), allowing for an interaction between chronological age and RU486 concentration.

The results show that the conditions leading to ML extension also lead to a slower increase in Smurf's prevalence (Figure 6b, point ii). This was not the case for conditions not leading to different ML (Figure S17). These results suggest that the KD of the studied genes increases the ML by extending the non‐Smurf period of life, possibly because these genes modulate early steps of ageing. Interestingly, the three genes we validated for their role in longevity are reported to possibly interact based on the STRING database (Szklarczyk et al., 2021) (Figure 6c). We failed to identify any significant increase of ML on males (Figure S18).

## 3 DISCUSSION

This study describes how the long defined transcriptional signature of ageing and associated ‘ageing transcriptional hallmarks’, instead of accompanying chronological age with continuous and progressive changes, actually behave in a biphasic manner. We identified this previously hidden behaviour thanks to the Smurf, two‐phase, model of ageing.

The detection of living individuals showing an increased IP to a very small (800 Da), non‐toxic, blue food dye previously allowed us to propose a model of ageing with two consecutive and necessary phases. Although a recent article by Bitner and colleagues (2020) suggests that only a low proportion of flies undergo the Smurf transition, our extensive characterisation of the phenotype using female flies from lines of different genetic backgrounds characterised by significantly different life expectancies ranging from 20 to 80 days (DGRP, *Drs*GFP, w^1118^, Canton‐S, Oregon‐R and w^Dahomey^) as well as in F1 individuals, monitored individually or in groups, unequivocally show that every female Drosophila dies as a Smurf. In addition, the Smurf phenotype also accompanies ageing in Drosophila males as shown by us and others. Failure by Bitner et al. to reproduce our results is likely due to their non‐standard protocol.

The mathematical model we developed (Tricoire & Rera, 2015) fitted reasonably well survival curves while allowing for a direct interpretation of the parameters. In addition, the hypothesis that every individual turns Smurf prior to death allowed us to predict the relatively constant remaining lifespan of Smurfs irrespective of their chronological age, which we then validated using multiple DGRP lines. Given our previous description of physiological hallmarks of ageing—loss of fertility, mobility, energy stores—segregating with Smurf individuals (and defining Smurfness as an objective indicator of frailty), we decided here to explore the behaviour of the transcriptional hallmarks of ageing in light of the Smurf state of individuals and their chronological age.

Distinguishing these two subpopulations allowed us to observe the gene expression noise doubling between young and old individuals, making the transcriptional noise (ATH6) the only transcriptional hallmark of ageing to display a time‐dependent behaviour in our study. This increase of noise in the gene expression level, often associated with transcriptional drift (Perez‐Gomez et al., 2020), is concomitant with the time‐dependent increasing risk for an individual to enter the second and last phase of life, the Smurf phase. Interestingly, interventions decreasing it were already shown to extend lifespan in nematodes (Rangaraju et al., 2015). Although recent single‐cell RNAseq questions the existence of such an increase in transcriptional noise with age (Gems & de Magalhães, 2021) others showed that it is sufficient to predict both chronological and biological age. Overall, our data corroborate that gene expression noise occurs especially with chronological age and questions its role in the Smurf transition (Schumacher & Meyer, 2023). Old non‐Smurfs also show some of ATH1 suggesting that inflammation could precede the Smurf transition at least in old individuals although we cannot exclude that at an advanced age, the likelihood of sampling pre‐Smurf or early Smurfs is high and this signal could be due to such individuals contaminating the non‐Smurf samples. Then, individuals in the Smurf phase undergo a dramatic shift in gene expression with over 3000 genes differentially expressed compared to age‐matched non‐Smurf individuals. More importantly, these genes span across the six ageing transcriptional hallmarks, systemic inflammation (ATH1), active stress response (ATH2), decreased mitochondrial/energy metabolism (ATH3) and altered protein translation (ATH4). Old Smurf individuals also show a worsening of their DNA repair pathways, cell cycle regulation pathways (ATH5), chromatin regulation and RNA processing (ATH6). Recently, David Gems and João Pedro de Magalhães questioned the position of the hallmarks of ageing as a paradigm. Our results here seem to support this questioning (Ibañez‐Solé et al., 2022).

Indeed, the hallmarks are defined as (1) manifesting in an age‐related fashion, (2) their accentuation accelerating ageing and (3) intervention on them leading to delay, reverse or stop ageing (López‐Otín et al., 2013). However, rather than causative of the process they appear to be markers of a terminal and, so far, non‐reversible phase of life except for the dysregulation of gene expression. Further characterisation of the chain of events might allow to discriminate between major theories of ageing such as ‘inflammageing’, ‘genome maintenance’ or ‘oxidative damage’. Can an evolutionary conserved hallmark of ageing characteristic of the Smurf phase of life be a driver of ageing? On the other hand, if ageing is not programmed, how can such a late‐life phase be so much evolutionarily conserved and molecularly stereotyped?

In addition, here we show how most of the pro‐longevity genetic interventions identified so far involve genes affected by the Smurf transition. Our longevity experiments in Drosophila demonstrate that it is possible to significantly increase lifespan by tuning the expression of TFs likely to explain the Smurf‐associated transcriptional signature (*Trl*, *Adf‐1* and CG4360/*Sag1*) and delay the time of entrance in the Smurf phase. Although moderate, these increases of health and lifespan were consistent across inducing conditions and independent experiment, while of a similar extent to longevity studies properly controlling for genetic background using the gene switch system. The fact that we do not detect an increase in lifespan in males does not invalidate the results obtained on females. Those results are in line with the physiological sexual dimorphism of Drosophila longevity, an issue which has been recently more investigated (Belmonte et al., 2020; Garratt, 2020; Regan et al., 2016) but they could also be due to a sex‐specificity of the transcriptional signature presented in this article or due to the weaker inducibility of the *da*GS driver (Tricoire et al., 2009).

Even though the aim of the paper was not to characterise the events occurring at the intestinal level—the IP is merely a marker of the last phase of life in our model—we detected alterations of cell junction components RNA as well as the JAK/STAT pathway and ECM remodelling proteins, suggesting a broad restructuring of tissues at the scale of the whole organism. This is reminiscent of the overall alteration of controlled epithelial permeability broadly affecting living organisms during ageing (Parrish, 2017). The recent demonstration that the Smurf phenotype is due to increased IP but also to decreased Malpighian tubules activity (Livingston et al., 2020) is supportive of organismal functional failure occurring in the Smurf phase. Whether it is what is called multivisceral failure in humans is under investigation, but it might highlight the use of the Smurf model of ageing for the study of other barriers, especially the blood‐brain barrier.

By questioning the place of the hallmarks of ageing within the ageing process, our study highlights the high relevance of using the Smurf phenotype in ageing studies across multiple model organisms thanks to its strong evolutionary conservation (Dambroise et al., 2016; Salazar et al., 2023). The absence of Smurf classification in the experimental design indeed results in a non‐negligible confounding factor altering the interpretability of the results. Taking into consideration the Smurf phenotype in ageing studies is key to taking into account the interindividual heterogeneity. As schematised in our graphical abstract, looking at age‐related phenotypes without the Smurf phenotype can lead to misinterpretation, attributing to advancing age what is actually due to an increased proportion of Smurf individuals. Based on our results, we anticipate that the Smurf phenotype will become a standard parameter in ageing research, not as a measurement of IP but rather as a marker for frail individuals in the last phase of their life. Its broad evolutionary conservation as well as the distinct molecular changes occurring in the two phases of ageing will certainly allow a deep reexamination of the evolutionary mechanisms at stake in the wide presence of ageing through living organisms.

## 4 MATERIALS AND METHODS

### 4.1 RNA‐seq: Experimental design

A synchronous isogenic population of *drosomycin*‐GFP (*Drs*‐GFP) Drosophila line was used for the RNA‐sequencing experiment (40 vials of 30 mated female flies). For the longevity recording, flies were transferred on fresh food and deaths scored on alternative days. Flies were sampled for the sequencing experiment at day 20 (80% survival), day 30 (50% survival) and day 40 (10% survival). Each sample is a mixture of eight flies. The sampling protocol for Smurfs and age‐matched non‐Smurfs is the following: all flies—the ones used for longevity and the ones used for sampling—are transferred on blue food overnight; at 9 a.m. 1 Smurf sample and age‐matched non‐Smurf are collected (Mixed samples), and all the remaining Smurfs are discharged; 5 h later, 2 Smurf and non‐Smurf samples are collected (5 h Smurfs), and all the remaining Smurfs are discharged; 24 h later, 3 Smurf and non‐Smurf samples are collected. Note that at 90% no 5 h Smurfs could be collected due to the low probability of flies turning Smurf at this age. After sampling, flies were immediately frozen in liquid N_2_ and stored at −80°C up to RNA extraction. Each time‐point has a minimum of three biological replicates.

### 4.2 RNA‐seq: Preprocessing

Sequencing was externalised to Intragen. Library preparation was done using ‘TruSeq Stranded mRNA Sample Prep Illumina’ kit and conducted on HiSeq4000 Illumina sequencer (paired‐end sequencing). Data preprocessing was performed on Galaxy (Afgan et al., 2018) server. Quality control was performed using FastQC (Babraham Bioinformatics, 2023) and resulted in no reads filtering. Reads were aligned with Hisat2 (Kim et al., 2019) on the reference *D. melanogaster* genome BDGP6.95. Reads count was performed with featureCounts (Liao et al., 2014), resulting in a raw counts matrix of 15,364 genes.

### 4.3 RNA‐seq: Analysis

Unless stated otherwise, all analysis were performed on R 3.5.3 and plots generated with ggplot2 3.3.5. PCA was performed using package DESeq2 1.22.2. Association of components with Smurfness and age was computed using the functions PCA and dimdesc from FactoMineR 2.4. tSNE was performed on package Rtsne 0.15. Sample‐to‐sample distance heatmap was computed using function dist from stats 3.5.3, and plotted using heatmap 1.0.12. PCA, tSNE and clustering analyses were performed using normalised counts additionally transformed with the *vst* DESeq2 function to stabilise the variance. For the tSNE analysis, the perplexity parameter was set to 10. Additional details on the analyses can be found in the Github repository. The main DEGs analysis was performed on DESeq2 1.22.2, while validation analysis on edgeR 3.24.3. Enrichmend analysis was performed with the Bioconductor package clusterProfiler 3.10.1, which calls fgsea 1.8.0; analysis was ran with the following parameters: nPerm = 15,000, minGSSize = 10, maxGSSize = 600. Enrichment plot was generated with the function emmaplot from the same package. Venn diagram (Figure 4d) was generated using eulerr Rshiny app. Pearson correlation for analysis in Figure 5c was computed with the cor() R function.

### 4.4 Proteomic data collection and analysis

*Drs*GFP Smurfs (8 h) and non‐Smurfs were sampled at 80 and 10% survival in quadruplicates of 10 females. Flies were quickly homogenised in 96 μL NU‐PAGE 1X sample buffer containing antiproteases and quickly spun to precipitate debris. Forty μL of samples was then loaded on a NU‐PAGE 10% Bis‐Tris gel prior to being sent for label free proteomics quantification.

### 4.5 Metabolomic data collection and analysis

*Drs*GFP Smurfs and non‐Smurfs were sampled at 50% survival. Each sample corresponds to a mixture of 20/30 individuals, for a total of 7 Smurf and 7 non‐Smurf samples. Drosophila were weighted to reach around 30 mg in a 2 mL‐homogenizer tube with ceramic beads (Hard Tissue Homogenizing CK28, 2.8 mm zirconium oxide beads; Precellys, Bertin Technologies, France). Then, 1 mL of ice‐cold CH_3_OH/water (9/1, −20°C, with internal standards) was added to the homogenizer tube. Samples were homogenised (3 cycles of 20 s/ 5000 rpm; Precellys 24, Bertin Technologies), and homogenates were then centrifuged (10 min at 15000*g*, 4°C). Supernatants were collected, and several fractions were split to be analysed by different Liquid and Gaz chromatography coupled with mass spectrometers (LC/MS and GC/MS) (Grajeda‐Iglesias et al., 2021). Widely targeted analysis by GC–MS/MS was performed on a coupling 7890A gas chromatography (Agilent Technologies) Triple Quadrupole 7000C (Agilent Technologies) and was previously described in (Durand et al., 2021). Polyamines, nucleotides, cofactors, bile acids and short chain fatty acids analyses were performed by LC–MS/MS with a 1260 UHPLC (Ultra‐High Performance Liquid Chromatography) (Agilent Technologies) coupled to a QQQ 6410 (Agilent Technologies) and were previously described in (Durand et al., 2021). Pseudo‐targeted analysis by UHPLC‐HRAM (Ultra‐High Performance Liquid Chromatography—High Resolution Accurate Mass) was performed on a U3000 (Dionex)/Orbitrap q‐Exactive (Thermo) coupling, previously described in (Abdellatif et al., 2021; Durand et al., 2021). All targeted treated data were merged and cleaned with a dedicated R (version 4.0) package (@Github/Kroemerlab/GRMeta). A total of 202 metabolites were detected. All the analysis presented (fold change estimation, Wilcoxon test and quantitative enrichment analysis) were done using MetaboAnlyst (Xia & Wishart, 2011). One Smurf sample was removed from the analysis as generated starting from eight individuals only, resulting in a total N of seven non‐Smurfs and six Smurfs. Samples were normalised by weight. Gene expression and metabolites representation KEGG maps were generated using pathview 1.2 (Luo & Brouwer, 2013) (R package).

### 4.6 Longevity experiments

All the flies are kept in closed vials in incubators at controlled temperature, humidity and 12 h light cycle. Experiments are carried at 26°C. Longevity experiments (included the one from where flies were sampled for the RNAseq) were run on the following food composition: 5.14% (w/v) yeast, 2.91\% (w/v) corn, 4.28% (w/v) sugar, 0.57% (w/v) agar and Methyl 4‐hydroxybenzoate (Moldex) at a final concentration of 5.3 g/L to prevent fungi contamination. Just after eclosion, flies are collected in tubes with food and RU486 (Figure 5a). Males and females are left together to mate for 48 h. After that time, males or females (depending on the experiment) are sorted in a number of 30 per vial, with 5 vials for each RU concentration (total *N* per concentration is 150). Flies are transferred to new vials with fresh food and scored three times per week (Monday, Wednesday, Friday). An exception are the first 2 weeks of the experiment, when females undergo an additional transfer on Saturday or Sunday due to the fertilised eggs altering the food composition. The food is prepared the day before the scoring (1.25 mL per vial) and stored at room temperature.

### 4.7 Lines used

*daGS* driver (provided by Tricoire laboratory, Université de Paris). *Bloomington stock* (*with associated targeted gene if GS*): *Drs‐*GFP 55707, *dmrt93B*, 27657; *Ets21C*, 39069; *Hey*, 41650; *kay*, 27722; *Mef2*, 28699; *rib*, 50682; *Ets96B*, 31935; *GATAd*, 34625; *GATAe*, 33748; *srp*, 28606; *NF‐yB*, 57254; *Aef1*, 80390; *CG4360*, 51813; *FoxP*, 26774; *Hsf*: 41581; *Trl* 41582. *FlyORF stock* (*with associated targeted genes*): *NF‐yB*, F001895; *CG4360*, F000063; *dmrt93B*, F000445; *Ets96B*, F000142; *Ets21C*, F000624; *srp*, F000720; *GATAd*, F000714; *Hsf*, F000699. VRDC stock (with associated gene): *Adf1*, 4278.

### 4.8 Smurf assay recording

Flies were transferred to food containing the blue dye FD&C #1 at 2.5\% (w/v) 24 h prior to Smurfs counting. The dye is added as the last component in the food preparation and dissolved in it. At the moment of the counting, flies were transferred back on normal food. All the flies are therefore spending the same amount of time on blue food, in order not to introduce bias in the counts. Note that with the following method we are not having information about the time at which the Smurfs are becoming such. However, as the Smurfs spend on average the same amount of time in this phase (Tricoire & Rera, 2015), recording the presence of a ‘mixed’ Smurf population provides a good estimation of their appearance in the population. Smurf counting was performed every 2 weeks while the population was in the survival plateau, and every week once it exited it.

### 4.9 RNA extraction and qPCR quantification

Extraction of RNA was performed using the Trizol protocol as in (Rio et al., 2010), adapted to the amount of tissue used. Each sample corresponds to a mixture of 3 flies for the RT‐qPCR experiments and eight flies for the RNA‐Seq. For the RT‐qPCRs, RNA was retro‐transcribed using the Applied Biosystems cDNA Reverse Transcription Kit. RT‐qPCR was subsequently performed using the Applied Biosystem PowerTrack SYBR Master Mix on Biorad CFX 96. Primers were designed on Benchling. *Adf1* Fw: ACAGCCCTTCAACGGCA, *Adf1* Rw: CGGCTCGTAGAAGTATGGCT; *CG4360* Fw: CAGCAGAGCACCCTTACCAA, *CG4360* Rw: GGAGCGGGCATTGAGTGAT; *Trl* Fw: TCCTATCCACGCCAAAGGCAAA, Trl Rw: TAGCAAATGGGGCAAGTAGCAGG; *Act* Fw: CCATCAGCCAGCAGTCGTCTA, *Act* Rw: ACCAGAGCAGCAACTTCTTCG.

## AUTHOR CONTRIBUTIONS

Michael Rera conceived the presented idea and model. Flaminia Zane and Michael Rera conceived, planned and performed the analysis and experiments as well as wrote the manuscript. Hayet Bouzid and Mira Brazane performed the RT‐qPCR experiments and analysed the results. Sofia Sosa Marmol and Julia Lisa Molina participated in the longevity experiments. Savandara Besse provided technical support for the analysis. Céline Cansell, Fanny Aprahamian and Sylvère Durand performed and analysed the metabolomics experiments. Jessica Ayache and Michael Rera performed and analysed the proteomics experiments. Christophe Antoniewski helped with the RNAseq analysis. Clément Carré helped with the RTqPCR design and the manuscript writing. All authors discussed the results and contributed to the final manuscript.

## FUNDING INFORMATION

Michael Rera is funded by the CNRS, Flaminia Zane is funded by Sorbonne Université Interdisciplinary research PhD grant. This project was funded by the ANR ADAGIO (ANR‐20‐CE44‐0010) and the ATIP/Avenir young group leader program for MR. Thanks to the Bettencourt Schueller Foundation long term partnership, this work was partly supported by the CRI Core Research Fellowship to Michael Rera. Michael Rera and Clément Carré received support from the IBPS‐2020 Action Incitative.

## CONFLICT OF INTEREST STATEMENT

The authors declare no conflicts of interest.

## Supporting information


Data S1.

## Data Availability

All codes and associated processed data are available at https://github.com/MichaelRera/SmurfsTrsc Raw RNAseq data are available at NCBI Geo https://www.ncbi.nlm.nih.gov/geo/query/acc.cgi?acc=GSE219063.
