# Supplementary material for: Smurfness‐based two‐phase model of ageing helps deconvolve the ageing transcriptional signature
Source: Aging Cell. 2023 Oct 12;22(11):e13946. doi: 10.1111/acel.13946 (PMC10652310; doi:10.1111/acel.13946)
Supplement: Supplementary file 1 — Data S1. [file ACEL-22-e13946-s001.pdf]

Supplementary Figures

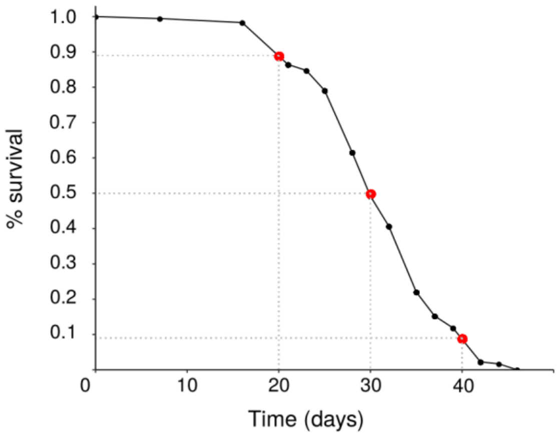

S1. Survival curve of the *Drs*GFP population used for the RNA-seq experiment. Red dots (and axis intersecting dotted lines) highlight the sampling time points (20 days - 90% survival, 30 days - 50% survival, 40 days - 10% survival).

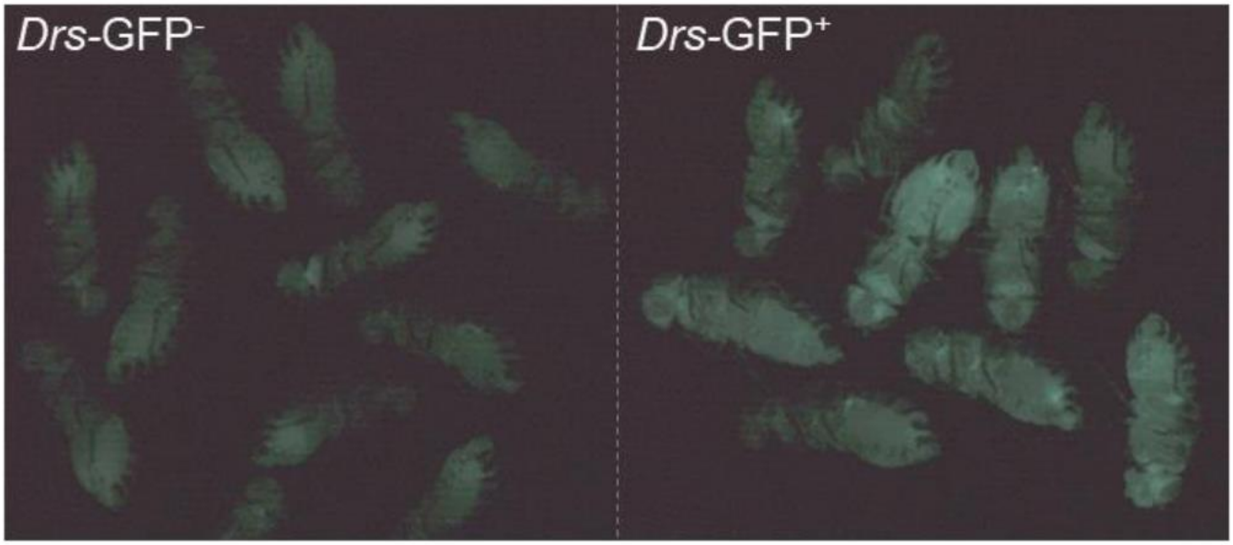

S2. GFP$^+$ and GFP$^-$ flies from *Drs*-GFP line. Flies separated based on the GFP activation status (GFP$^-$ on the left and GFP$^+$ on the right), a possible alternative method for Smurf selection.

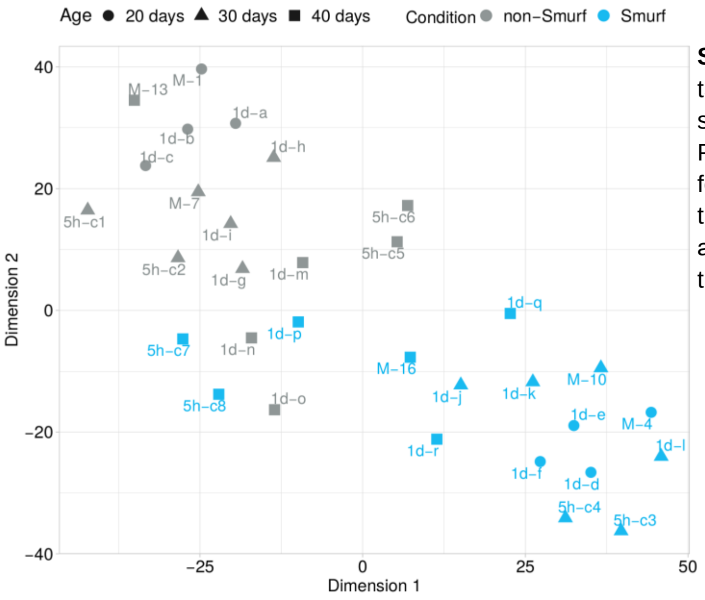

S3. tSNE (perplexity = 10) on RNA-seq samples. tSNE is computed on all genes. Colour indicates Smurf status, symbols the age (as in legend). Similarly to the PCA results in Fig. 1a, Smurf and non-Smurfs samples form two groups. In the non-Smurf groups we can notice the samples segregating by age, while the Smurf group appear more mixed. The same "mixed" behaviour for the 40 days samples as in the PCA are identified.

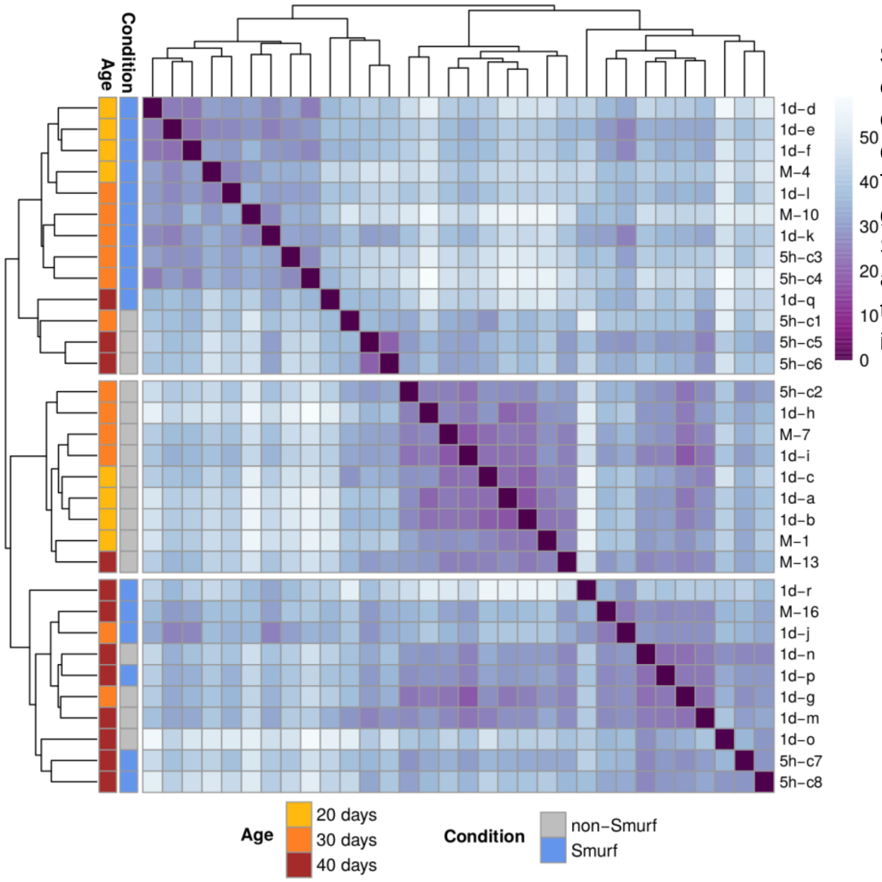

S4. Unsupervised hierarchical clustering on sample-to-sample distance. Distance matrix (euclidean distance) is computed using all genes. Three main clusters are identified, showing good separation between Smurfs and non Smurfs but for the 40 days samples, which appear to either correlate with one of the two groups or form a third cluster independently of the Smurf status.

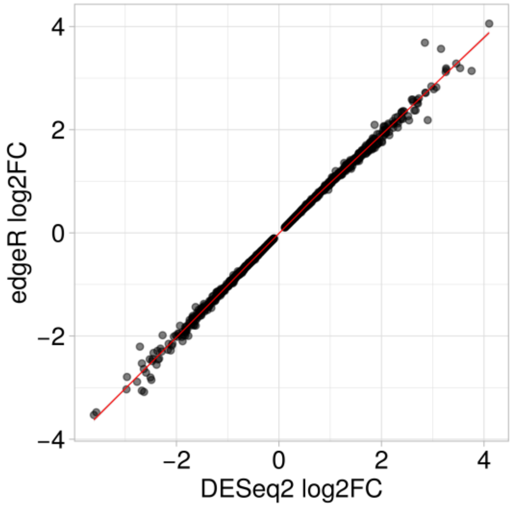

S5. edgeR pipeline validates DESeq2 analysis. Each of the commonly 2362 DEGs identified by the two pipelines is plotted as a function of the estimated fold changes. Estimated pearson correlation between the two is 0.99.

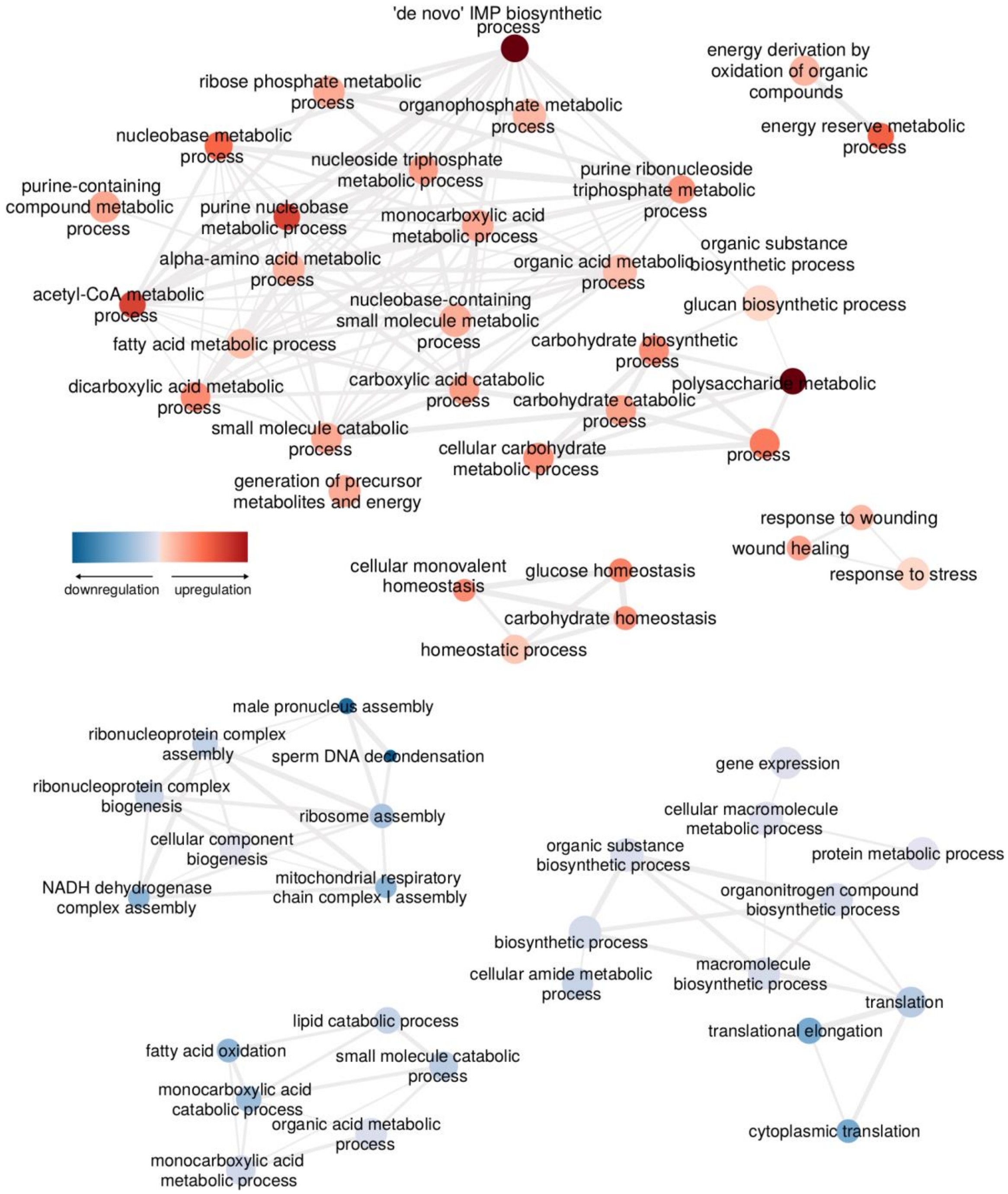

S6. Enrichment analysis on differentially expressed proteins, Smurf vs non-Smurfs. Interconnected GO BP significant categories are here represented as a network. The color indicates the level of deregulation (Panther Fold Change estimation) - http://www.pantherdb.org/ - . The node size provides an approximate indication of the GO BP category size. Amongst the upregulated categories we mostly observe response to stress and proteins involved in metabolism (with a strong signal coming from the IMP biosynthesic process category - associated to purine metabolism, not observed in the transcriptome). The downregulated categories mostly map to ribosomal proteins, mitochondrial respiratory chain (complex I), metabolism (with the lipid catabolic process confirming what is observed in the Smurf transcriptome). The gene expression categories include numerous ribosomal proteins and should therefore not be interpreted as a signal regarding transcriptional regulation.

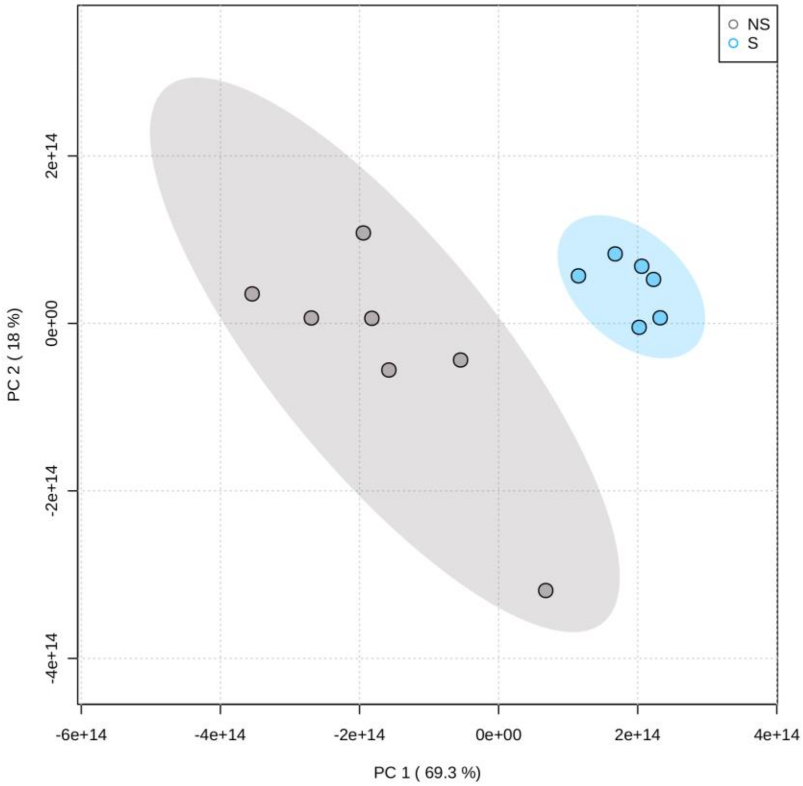

S7. PCA performed on metabolomic data. Similarly to what occurs for th transcriptomic, the PCA on the quantification of 202 metabolites clearly separates Smurf and non-Smurf samples. PCA performed through MetaboAnalyst online platform.

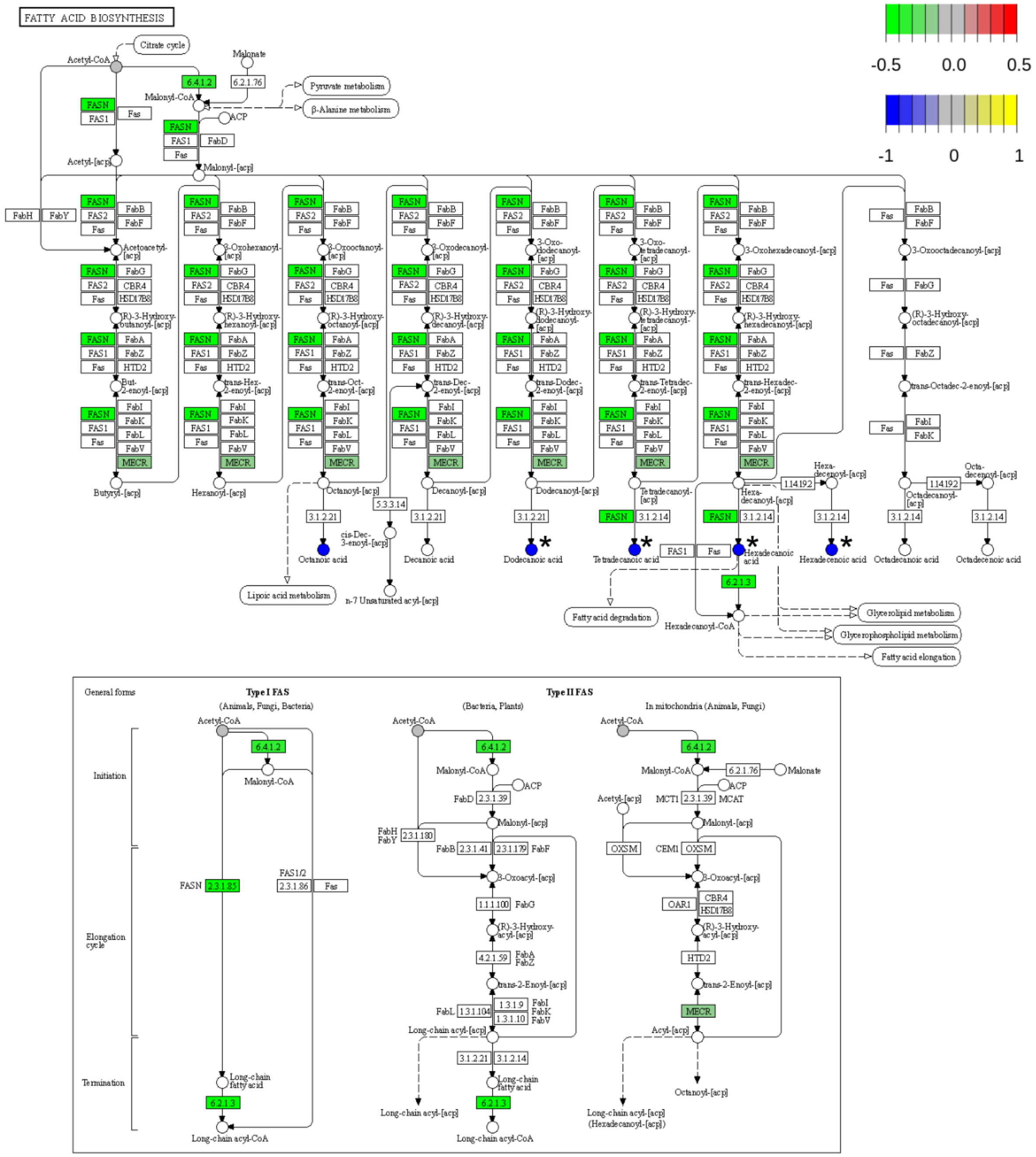

S8. Smurf DEGs and metabolites FC on KEGG fatty acid biosynthesis pathway (dm00061). The pathview R package is used to map the genes identified as DEGs in the Smurf/non-Smurf comparison and belonging to the KEGG fatty acid pathway (dm00061). The log_2FC estimated by the DESeq2 analysis is represented by the color scale. The detected metabolites are colored according Smurf/non-Smurf log_2FC, and associated to a * when significant to Wilcoxon test (p-value < 0.05). The downregulation of biosynthesis-mediating enzymes is associated by a decreased presence in Smurfs of the final fatty acid products, suggesting that the transcriptional signature is functional. Enzymatic complexes are annotated through unique identification code, while genes are automatically annotated with the humane symbol. To retrieve the Drosophila gene symbols from pathway's nodes, go to the online version and place the pointer on the gene on interest (https://www.genome.jp/pathway/dme00061).

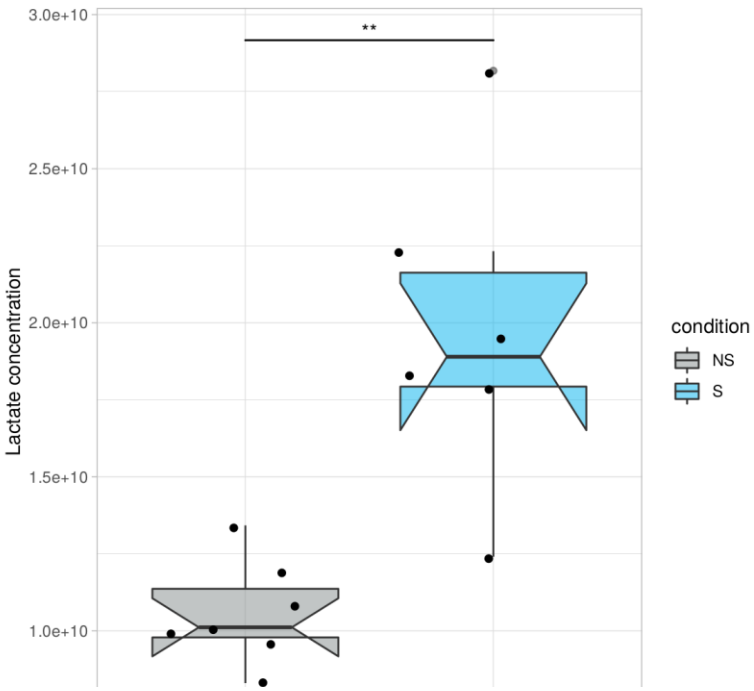

S9. Lactic acid levels are significantly higher in Smurfs. Smurf present a significant increase in lactic acid level ($log_2FC = 0.90$; **p-value < 0.001) compared to non-Smurfs. This confirms that the transcriptional upregulation of *Ldh* in Smurfs is functional. Smurfs might rely more on fermentation after glycolysis (compared to the non-Smurfs) given the general impairment experience in mitochondria.

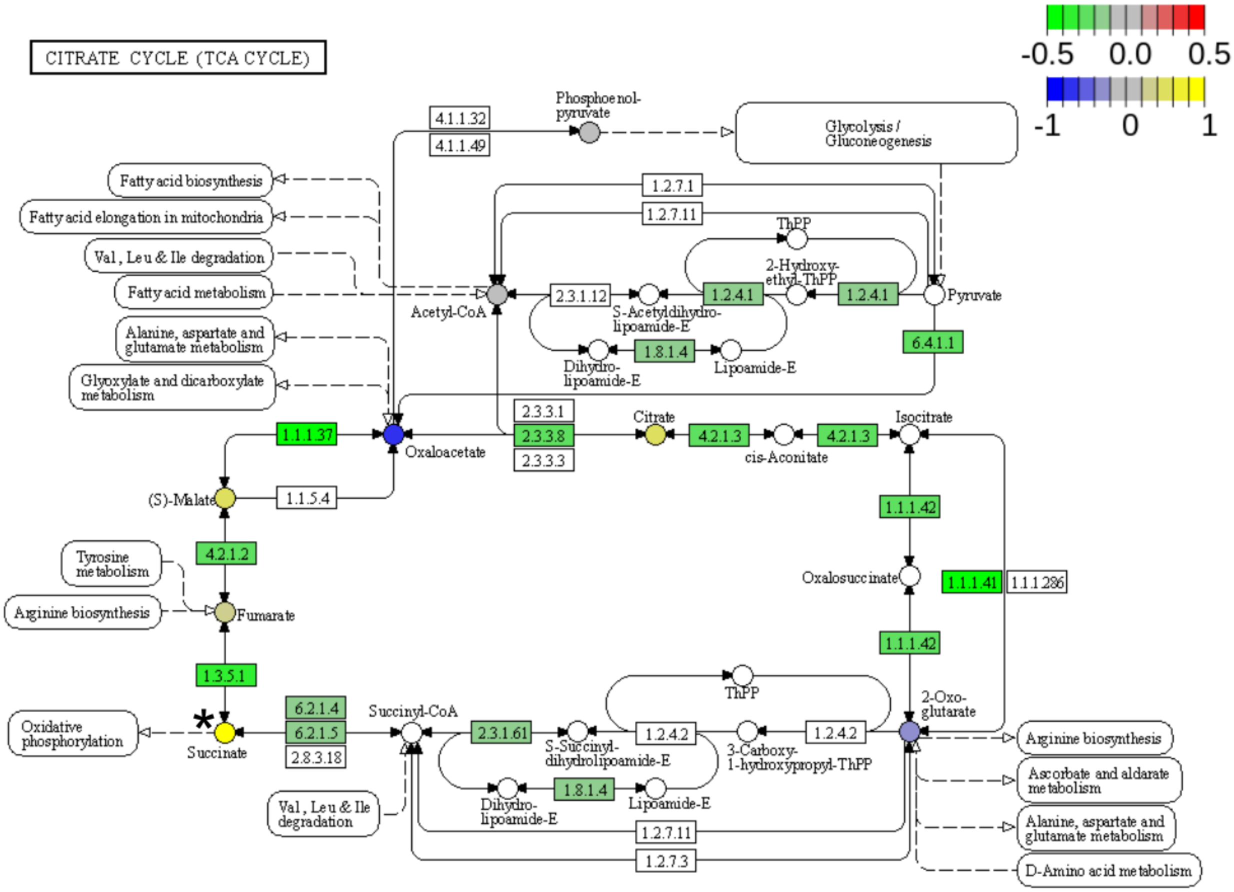

S10. Smurf DEGs and metabolites FC on KEGG TCA cycle pathway (dm00061). The pathview R package is used to map the genes identified as DEGs in the Smurf/non-Smurf comparison and belonging to the KEGG fatty acid pathway (dm00061). The log_2FC estimated by the DESeq2 analysis is represented by the color scale. The detected metabolites are colored according Smurf/non-Smurf log_2FC, and associated to a * when significant to Wilcoxon test (p-value < 0.05). As already discussed in the Smurf transcriptome characterization, the TCA cycle displays wide downregulation. At a metabolomic level, the pathway missed the threshold for significance in the quantitative enrichment analysis (FDR = 0.13), and only succinate is significant to Wilcoxon test (log_2FC = 1.28 , p-value < 0.05). However, given the general impairment of mitochondrial metabolism observed at a transcriptomic and proteomic level, we believe the trend observed in the metabolomic data could still be interesting and serve as hypothesis generator for further analysis. Enzymatic complexes are annotated through unique identification code, while genes are automatically annotated with the humane symbol. To retrieve the Drosophila gene symbols from pathway's nodes, go to the online version and place the pointer on the gene of interest (https://www.genome.jp/pathway/map00020).

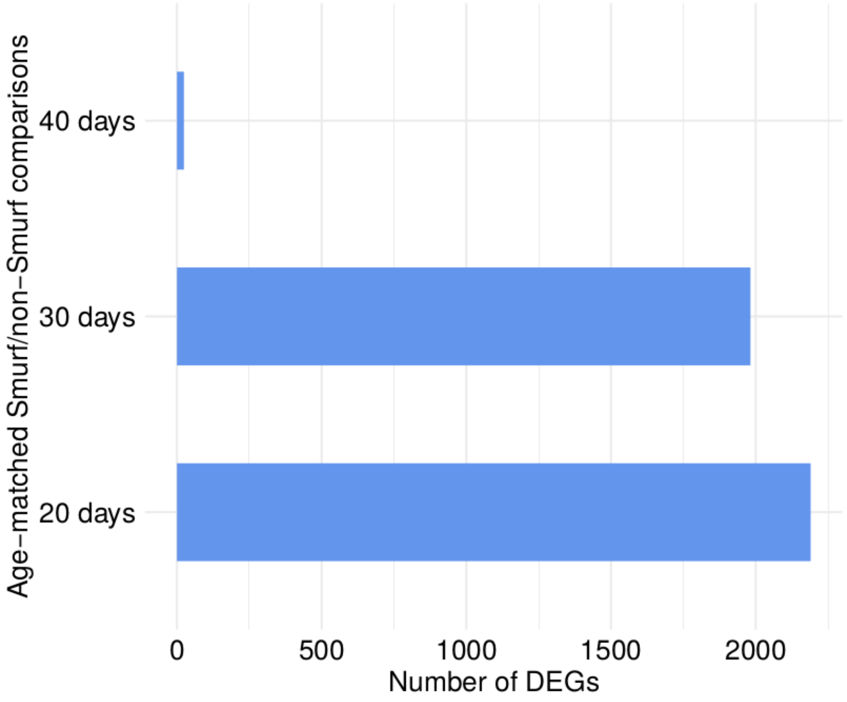

S11. The number of DEGs in age-matched Smurf/non-Smurf comparisons decreases with chronological age. When comparing age-matched Smurfs and non-Smurfs, different number of DEGs are retrieved ($DEGs_{20}$ = 2190 , $DEGs_{30}$ = 1982 , $DEGs_{40}$ = 24). The dramatic drop of DEGs at 40 days suggests that the transcriptome of old Smurfs and non-Smurfs are more similar than at younger ages. This was already suggested by the PCA (Fig. 1a) and might suggest that the old non-Smurfs samples, collected in the old population, are enriched in pre-Smurfs compared to their younger counterparts.

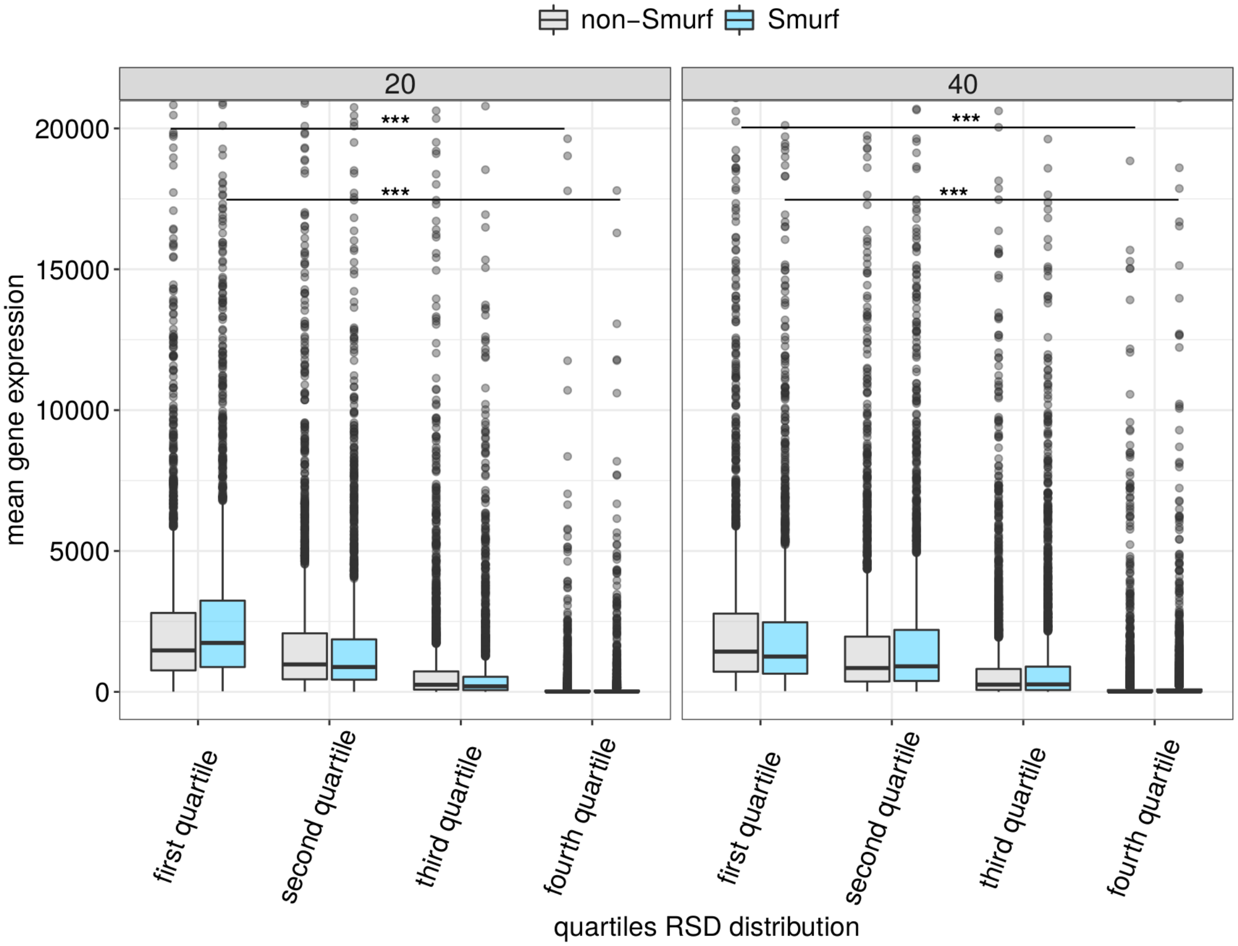

S12. Higher relative standard deviation (RSD) in gene expression in our dataset is associated to lower counts. We divided the RSD distributions in Fig. 4b into the four quartiles (x axis) and plotted the mean gene expression of the associated genes (y axis) for Smurfs and non-Smurfs at 20 and 40 days. The mean gene expression shows a decreasing trend over the four group, proved by the significant difference between the mean gene expression of the first and fourth quartile for both Smurf and non-Smurf at 20 and 40 days (wilcoxon test, p-value < 10^{e-16}).

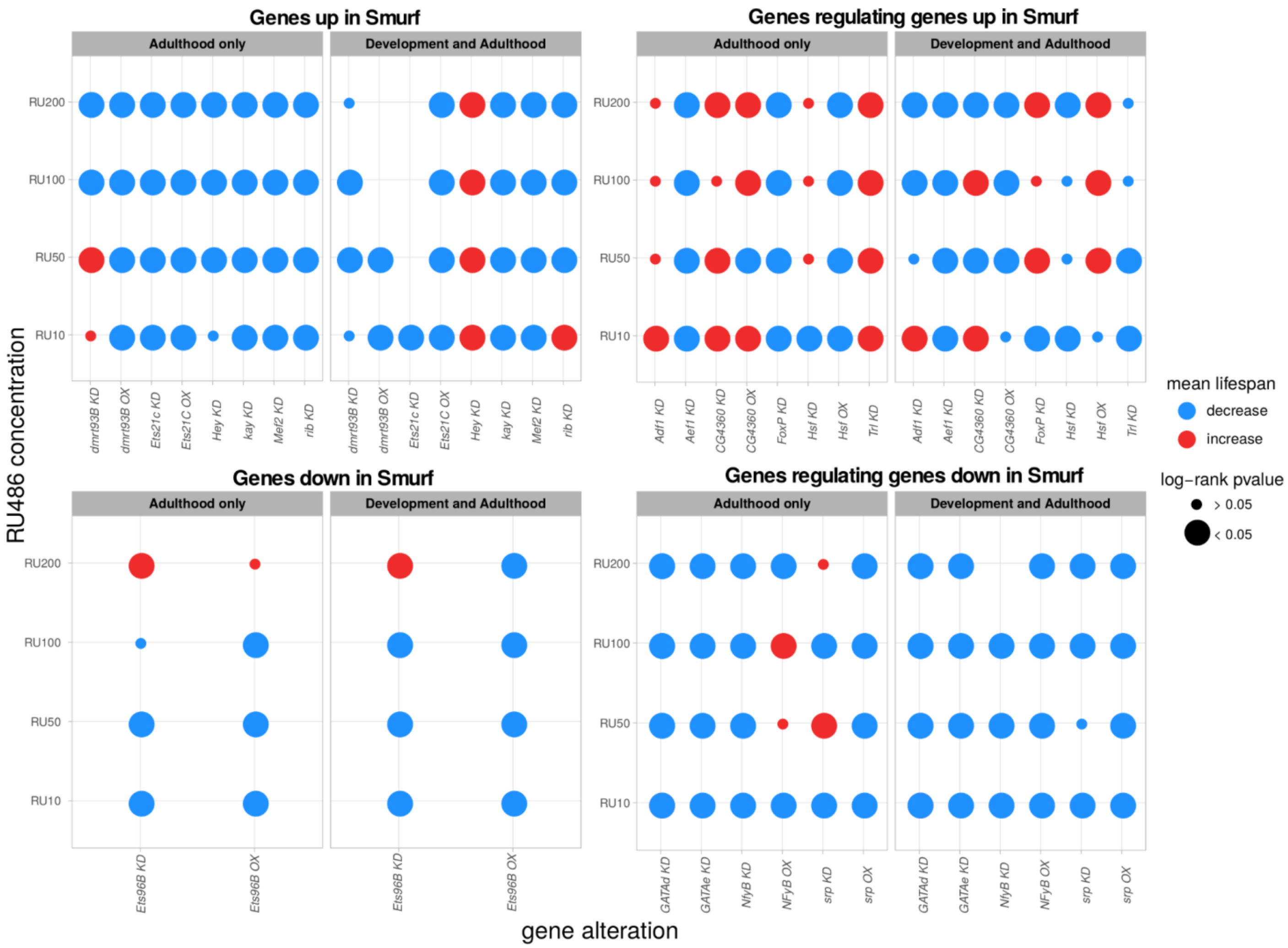

S13. Longevity screening results. Summary of the results of the longevity screen carried out on the genes listed in Table 1. For each experiment, the 4 RU486 treatments and the two experimental setting ("adulthood only" and "development & adulthood") are listed. The controls are not represented as they are the reference for the statistical test (log-rank) and computation of the mean lifespan change. The size of the the point indicates the significance of the difference in the longevity curve (treatment compared to control), while the colour indicates the direction of the change - decrease or increase of mean lifespan. In most cases we detected a significant difference with negative effect on the populations' lifespan (blue large points). Interestingly most of the positive hits (red large points) map to the group of genes found by i-cisTarget as putative regulators of TFs up in Smurfs.

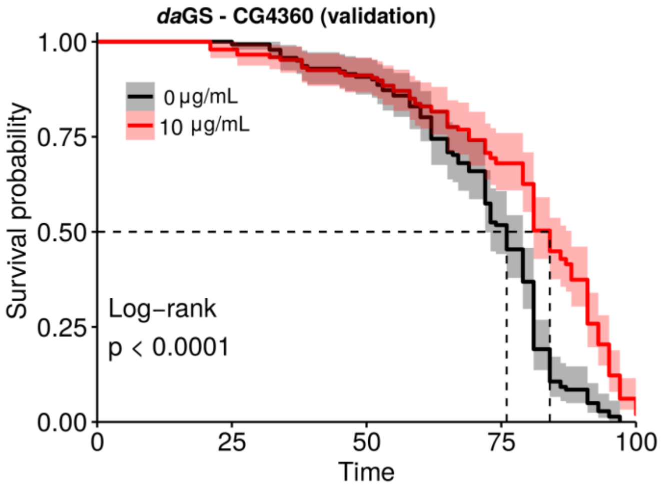

S14. CG4360 KD (adulthood & development) validation. The effect showed in Fig.7 on the CG4360 KD (adulthood & development setting), RU10 µg/mL, is confirmed by a third independent experiment. The effect is not observed on the "adulthood only" setting. The dotted line point at the median lifespan of the populations. The effect on the mean lifespan (ML) is + 9.5% (ML_{RU0} = 71.5, ML_{RU10} = 78.5).

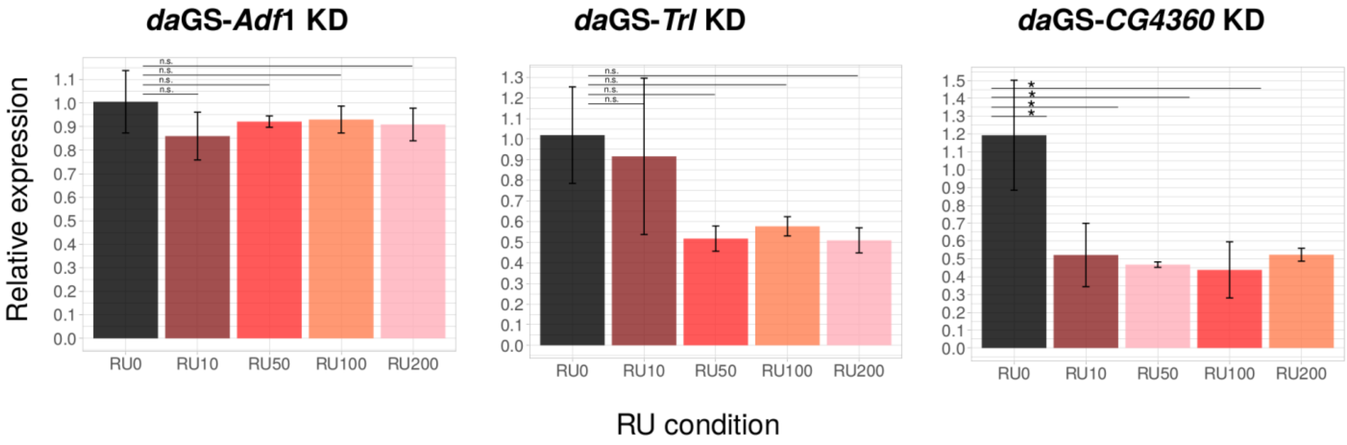

S15. Relative gene expression quantification on KD lines (*da*GS- *Adf1*, *Trl*, *CG4360*). qPCR results for quantification of Adf1, Trl and CG4360 expression in the respective KD lines (whole body RNA extraction). In no case we observe the expected gradient, suggesting that depending on the line the RU486 induction is more or less strong independently of the amount of drug. A significant downregulation (Wilcoxon test) is detected for CG4360 at all RU486 concentrations. However, even if a trend is noticeable for *Trl* and *Adf1*, the difference in relative expression is not significant to Wilcoxon test. p-value: * = 0.05, n.s. > 0.05. *Adf1*: average 2^-ΔΔCt RU0 = 1.006, RU10 = 0.860, RU50 = 0.921, RU100 = 0.930, RU200 = 0.909; SD (standard deviation) RU0 = 0.132, RU10 = 0.101, RU50 = 0.024, RU100 = 0.057, RU200 = 0.069. *Trl*: average 2^-ΔΔCt RU0 = 1.019 , RU10 = 0.916, RU50 = 0.518, RU100 = 0.577, RU200 = 0.509; SD RU0 = 0.234, RU10 = 0.380, RU50 = 0.061, RU100 = 0.046, RU200 = 0.060; *CG4360*: average 2^-ΔΔCt RU0 = 1.051 , RU10 = 0.522, RU50 = 0.468 , RU100 = 0.438, RU200 = 0.523; SD: RU0 = 0.379, RU10 = 0.177 , RU50

= 0.015 , RU100 = ,0.157, RU200 = 0.036. N = 3 sample for each RU concentration, where 1 sample is the mixture of 3 flies.

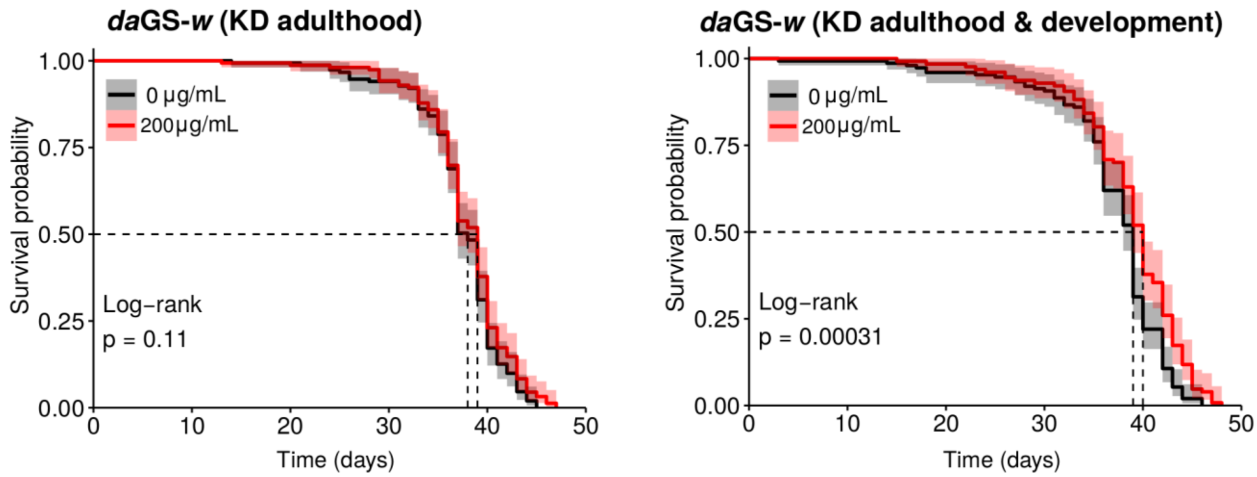

S16. RU486 treatment does not affect lifespan. In order to confirm that the RU486 treatment alone does not affect lifespan, we performed GS longevity experiment with the daGS driver inducing *w* KD (white KD does not affect longevity). We induced the GS with RU 200 µg/mL, corresponding to the highest treatment used in our longevity experiments. No significant difference in the longevity curves is detected in the "adulthood only" setting (ML_{RU0} = 37.4, ML_{RU200} = 37.9, p-value in figure). A significant difference is detected in the "adulthood & development" setting (ML_{RU0} = 37.0, ML_{RU200} = 38.7, p-value in figure). However, the modest effect (+4.5%), together with the overlap of the confidence intervals of the curves, suggest that the effect is not biologically relevant.

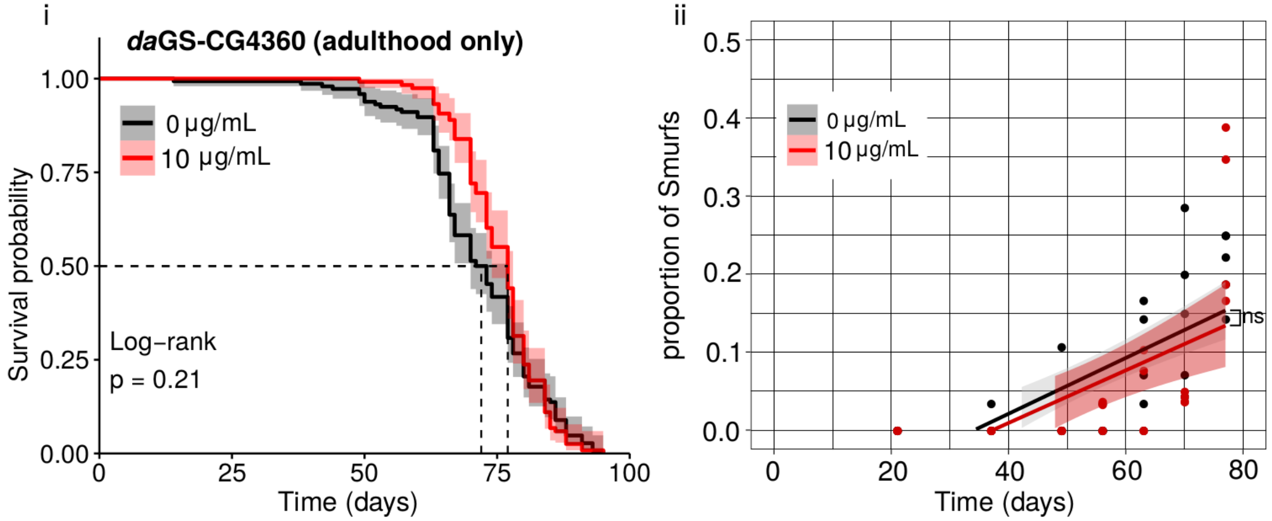

S17. Two populations with non-significantly different lifespan experience the same Smurf proportion increase over time: the example of *CG4360* KD (adulthood only). (i) Longevity experiment. *CG4360* does not extend lifespan when knocked-down during adulthood only (ML_{RU0} = 71.6, ML_{RU10} = 75.5, log-rank p-value = 0.21). **(ii) Smurf proportion evolution over time.** The Smurf proportion significantly increases over time in the populations ($slope_{RU0}$ = 0.0036, $p\text{-value}_{RU0}$ = 1.50e-06, $slope_{RU10}$ = 0.0034 , $p\text{-value}_{RU10}$ = 6.12e-04). However, no significant difference is detected between the slope of the control and the treated population (p-value = 0.84), contrary to what observed when the populations have significantly different lifespan (Fig. 6b).

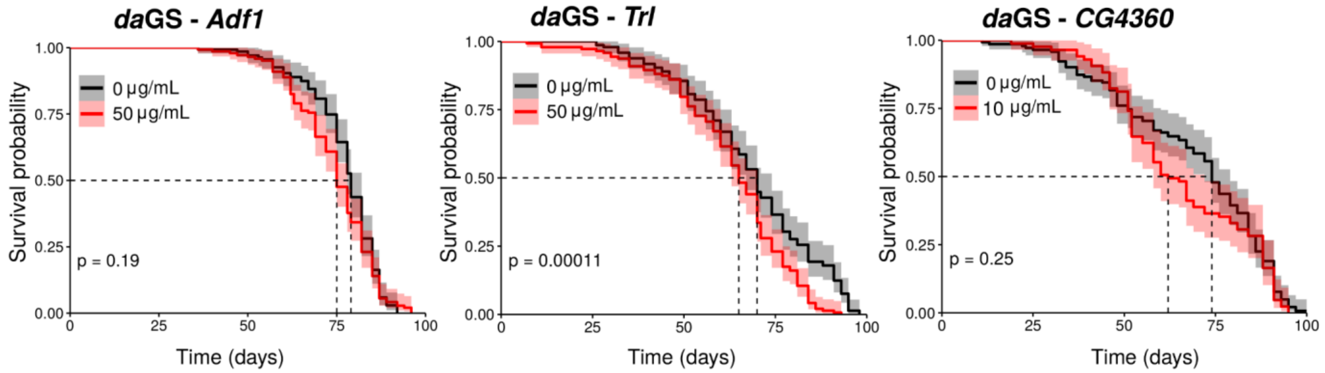

S18. Longevity experiments on males. In order to investigat if the longevity effect found on females applies to males, we performed the experiment on males from the same GS line. Results are reported for the condition extending lifespan on females (RU50 µg/mL, adulthood only, for *Adf1* and *Trl*; RU10 µg/mL, development & adulthood, for *CG4360*). No significant effect is detected for *Adf1* and *CG4360* (log-rank p-values reported in figure; *Adf1*: ML_{RU0} = 77.1 , ML_{RU50} = 74.4; *CG4360*: ML_{RU0} = 68.7 , ML_{RU10} = 65.8). A significant negative effect is detected for *Trl* KD (*Trl*: ML_{RU0} = 68.5 , ML_{RU50} = 62.9, -8.1%). However, the longevity curves are evolving similarly and the confidence intervals are diverging only after the T_{50} ; this suggests that the results need to be interpreted carefully, as the significance might not imply biological relevance.

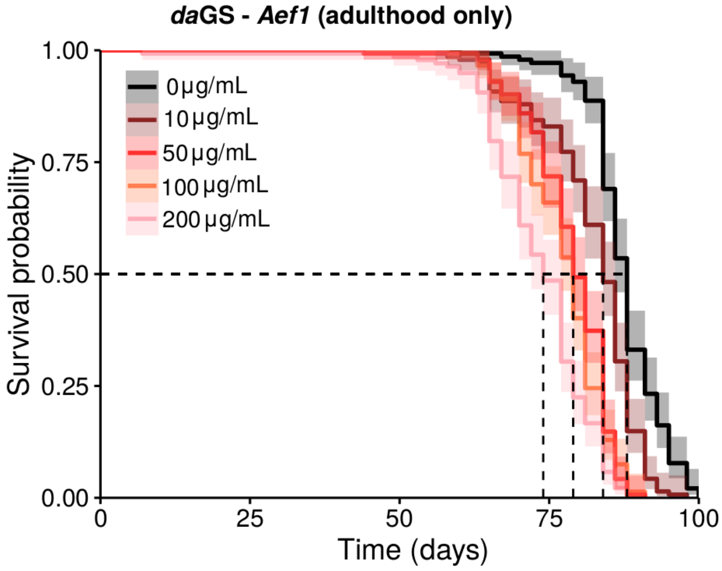

Fig S19. *Aef1* KD negatively affects life expectancy following the treatment gradient. *Aef1* KD negatively affects lifespan at all doses (ML_{RU0} = 87.6, ML_{RU10} = 82.2, ML_{RU50} = 78.6, ML_{RU100} = 77.4, ML_{RU200} = 73.3; p-value < 0.00001 for the log-rank test, details in Table S14). Dashed lines in figure indicate the median lifespan. The dose-dependent trend suggested by the ML values is confirmed when comparing the longevity curves of the treated populations, with only the RU50 and RU100 showing no significant difference (RU10-RU50: p-value = 3e-10 ; RU50-RU100: p-value = 0.2 ; RU100-RU200: p-value = 2e-04). Such trend suggest an effect on longevity of *Aef1* rather than a toxic effect of the KD.

Supplementary Tables

GO BP category	size	NES	p.adjust
GO:0050829 defense response to Gram-negative bacterium	39	2.343910	**
GO:0019731 antibacterial humoral response	29	2.335396	**
GO:0070482 response to oxygen levels	35	2.322513	**
GO:0006457 protein folding	44	2.273671	**
GO:0019730 antimicrobial humoral response	53	2.272312	**
GO:0006458 'de novo' protein folding	15	2.253483	**
GO:0051084 'de novo' posttranslational protein folding	15	2.253483	**
GO:0051085 chaperone cofactor-dependent protein refolding	15	2.253483	**
GO:0061077 chaperone-mediated protein folding	25	2.249745	**
GO:0006986 response to unfolded protein	16	2.241839	**
GO:0035966 response to topologically incorrect protein	16	2.241839	**
GO:0034620 cellular response to unfolded protein	15	2.234994	**
GO:0035967 cellular response to topologically incorrect protein	15	2.234994	**
GO:0042026 protein refolding	15	2.116671	**
GO:0006959 humoral immune response	63	2.092197	**
GO:0001666 response to hypoxia	23	2.082786	*
GO:0036293 response to decreased oxygen levels	26	2.057510	**
GO:0042742 defense response to bacterium	80	2.042555	**
GO:0051276 chromosome organization	61	2.027083	**
GO:0050830 defense response to Gram-positive bacterium	32	2.011920	*
GO:0031347 regulation of defense response	33	1.971860	*
GO:0006955 immune response	107	1.951750	**
GO:0061057 peptidoglycan recognition protein signaling pathway	10	1.935157	*
GO:0006952 defense response	138	1.905159	**
GO:0098542 defense response to other organism	103	1.869388	*
GO:0009617 response to bacterium	97	1.844119	*
GO:0009266 response to temperature stimulus	48	1.789309	*
GO:0002376 immune system process	137	1.789089	**
GO:0009628 response to abiotic stimulus	125	1.749653	*
GO:0071310 cellular response to organic substance	75	1.745146	*
GO:0009607 response to biotic stimulus	138	1.732527	**
GO:0043207 response to external biotic stimulus	138	1.732527	**
GO:0051707 response to other organism	138	1.732527	**
GO:0006950 response to stress	356	1.731664	**

GO:0019538	protein metabolic process	534	-1.395434	*
GO:0055086	nucleobase-containing small molecule metabolic process	117	-1.614257	*
GO:0055114	oxidation-reduction process	225	-1.618842	*
GO:0008610	lipid biosynthetic process	68	-1.728761	*
GO:0015980	energy derivation by oxidation of organic compounds	52	-1.797256	*
GO:0006091	generation of precursor metabolites and energy	73	-1.811853	*
GO:0016054	organic acid catabolic process	54	-1.817399	*
GO:0046395	carboxylic acid catabolic process	54	-1.817399	*
GO:0044281	small molecule metabolic process	314	-1.850554	*
GO:0007304	chorion-containing eggshell formation	30	-1.878965	*
GO:0042254	ribosome biogenesis	46	-1.902000	*
GO:0042180	cellular ketone metabolic process	20	-1.904478	*
GO:0042775	mitochondrial ATP synthesis coupled electron transport	25	-1.911838	*
GO:0030703	eggshell formation	31	-1.924518	*
GO:0022904	respiratory electron transport chain	26	-1.933564	*
GO:0042773	ATP synthesis coupled electron transport	26	-1.933564	*
GO:0044282	small molecule catabolic process	72	-1.937346	*
GO:0022900	electron transport chain	27	-1.956907	*
GO:0034754	cellular hormone metabolic process	13	-1.986510	*
GO:0045455	ecdysteroid metabolic process	13	-1.986510	*
GO:0045333	cellular respiration	39	-1.998705	*
GO:0006508	proteolysis	267	-2.029165	*
GO:0042445	hormone metabolic process	21	-2.039538	*
GO:0016125	sterol metabolic process	11	-2.125654	**
GO:0008202	steroid metabolic process	19	-2.179092	**

Table S1. GSEA results, Smurf/non-Smurf analysis. List of the 59 significant deregulated GO BP categories (adjusted p-value < 0.05) from the GSEA analysis on the list of Smurf DEGs. Results are illustrated in Fig. 2 of the main text. GO BP category: ID and description of the biological process category; size: number of genes annotated in the category; NES: normalized enriched score; p.adjust: FDR correction on the p-value, * < 0.05, ** < 0.01.

Metabolite Set	Total	Hits	FDR
Valine, leucine and isoleucine degradation	40	3	3.4984E-4
Biosynthesis of unsaturated fatty acids	36	5	5.067E-4
alpha-Linolenic acid metabolism	13	2	5.067E-4
Linoleic acid metabolism	5	1	5.067E-4
Fatty acid biosynthesis	47	4	0.0020546
Fatty acid degradation	39	3	0.0020546
Fatty acid elongation	38	2	0.0020546
Pyruvate metabolism	22	6	0.0071512
Valine, leucine and isoleucine biosynthesis	8	2	0.0078342
Phenylalanine metabolism	10	3	0.043592
Phenylalanine, tyrosine and tryptophan biosynthesis	4	2	0.043592
Thiamine metabolism	7	1	0.051592
Cysteine and methionine metabolism	33	5	0.059126

Table S2. Quantitative enrichment analysis on metabolites profile (S/NS), significant hits. Quantitative enrichment analysis on metabolites quantification (from MetaboAnalyst) results in 13 significant KEGG pathways. The TCA cycle missed the 5% significant threshold (FDR = 0.13), but most of the associated metabolites are present in the pyruvate metabolism pathway. In confirmation of what is seen with the transcriptomic, we find fatty acid metabolism associated pathways. A signal from amino acids metabolism is also detected. Metabolite set: KEGG pathway; Total: number of metabolites in the pathway; FDR: adjusted p-value.

GO BP category	size	NES	p.adjust
GO:0010038 response to metal ion	15	1.983838	*
GO:0010035 response to inorganic substance	22	1.782223	*
GO:0006030 chitin metabolic process	25	1.778918	*
GO:1901071 glucosamine-containing compound metabolic process	27	1.775833	*
GO:0006022 aminoglycan metabolic process	29	1.750849	*
GO:0006040 amino sugar metabolic process	28	1.737450	*
GO:0007606 sensory perception of chemical stimulus	24	1.622532	*
GO:0055114 oxidation-reduction process	134	1.526567	*
GO:0005975 carbohydrate metabolic process	68	1.515730	*
GO:0017144 drug metabolic process	64	1.513947	*
GO:1901988 negative regulation of cell cycle phase transition	17	-1.979813	*
GO:1901991 negative regulation of mitotic cell cycle phase transition	17	-1.979813	*
GO:0034470 ncRNA processing	26	-1.998833	*
GO:0051129 negative regulation of cellular component organization	37	-2.000688	*
GO:0045930 negative regulation of mitotic cell cycle	19	-2.006150	*
GO:0043254 regulation of protein complex assembly	28	-2.009431	*
GO:0022613 ribonucleoprotein complex biogenesis	34	-2.026966	*
GO:0010769 regulation of cell morphogenesis involved in differentiation	23	-2.043760	*
GO:0006342 chromatin silencing	25	-2.044283	*
GO:0045814 negative regulation of gene expression, epigenetic	25	-2.044283	*
GO:0030951 establishment or maintenance of microtubule cytoskeleton polarity	13	-2.050560	*
GO:0050770 regulation of axonogenesis	14	-2.057130	*
GO:0060810 intracellular mRNA localization involved in pattern specification process	14	-2.082873	*
GO:0060811 intracellular mRNA localization involved in anterior/posterior axis specification	14	-2.082873	*
GO:0043085 positive regulation of catalytic activity	24	-2.084854	*
GO:0007338 single fertilization	11	-2.090751	*
GO:0009566 fertilization	11	-2.090751	*
GO:1990778 protein localization to cell periphery	14	-2.098238	*
GO:0031400 negative regulation of protein modification process	15	-2.104281	*
GO:0030952 establishment or maintenance of cytoskeleton polarity	15	-2.111130	*
GO:0007265 Ras protein signal transduction	34	-2.117713	*
GO:0051052 regulation of DNA metabolic process	18	-2.127928	*
GO:0046578 regulation of Ras protein signal transduction	17	-2.145951	*
GO:0044786 cell cycle DNA replication	12	-2.148274	*
GO:0006399 tRNA metabolic process	19	-2.161945	*
GO:0008298 intracellular mRNA localization	15	-2.164617	*
GO:0033047 regulation of mitotic sister chromatid segregation	11	-2.168912	*
GO:0006402 mRNA catabolic process	12	-2.170716	*
GO:0051056 regulation of small GTPase mediated signal transduction	18	-2.179228	*
GO:0007093 mitotic cell cycle checkpoint	11	-2.194288	*
GO:0010638 positive regulation of organelle organization	35	-2.202552	*
GO:0043087 regulation of GTPase activity	12	-2.225064	*
GO:0000075 cell cycle checkpoint	16	-2.228099	*
GO:0022618 ribonucleoprotein complex assembly	20	-2.239742	*
GO:0031124 mRNA 3'-end processing	11	-2.253050	*
GO:0000281 mitotic cytokinesis	22	-2.253149	*
GO:1902275 regulation of chromatin organization	33	-2.259971	*
GO:0061640 cytoskeleton-dependent cytokinesis	35	-2.267458	*
GO:0000910 cytokinesis	36	-2.271666	*
GO:2001252 positive regulation of chromosome organization	20	-2.272650	*
GO:0006511 ubiquitin-dependent protein catabolic process	38	-2.274556	*
GO:0006302 double-strand break repair	10	-2.286608	*
GO:0010948 negative regulation of cell cycle process	22	-2.295652	*
GO:0034968 histone lysine methylation	12	-2.296995	*
GO:0010639 negative regulation of organelle organization	24	-2.302245	*

Table S3. GSEA analysis on old Smurfs/young Smurfs.

GO:0032984 protein-containing complex disassembly	15	-2.313278	*
GO:0000086 G2/M transition of mitotic cell cycle	11	-2.327275	*
GO:0044839 cell cycle G2/M phase transition	11	-2.327275	*
GO:0051303 establishment of chromosome localization	11	-2.327275	*
GO:0065004 protein-DNA complex assembly	22	-2.333056	*
GO:0007062 sister chromatid cohesion	11	-2.343420	*
GO:0000380 alternative mRNA splicing, via spliceosome	25	-2.345997	*
GO:0000381 regulation of alternative mRNA splicing, via spliceosome	25	-2.345997	*
GO:0034728 nucleosome organization	25	-2.347394	*
GO:0043161 proteasome-mediated ubiquitin-dependent protein catabolic process	29	-2.349185	*
GO:0031109 microtubule polymerization or depolymerization	11	-2.355110	*
GO:0071826 ribonucleoprotein complex subunit organization	23	-2.366604	*
GO:0016571 histone methylation	13	-2.374419	*
GO:0031056 regulation of histone modification	13	-2.374419	*
GO:0007307 eggshell chorion gene amplification	10	-2.376503	*
GO:0006333 chromatin assembly or disassembly	22	-2.378977	*
GO:0045786 negative regulation of cell cycle	29	-2.382539	*
GO:0072657 protein localization to membrane	19	-2.385931	*
GO:0007052 mitotic spindle organization	31	-2.394738	*
GO:0034660 ncRNA metabolic process	37	-2.397254	*
GO:0010498 proteasomal protein catabolic process	32	-2.399087	*
GO:0033045 regulation of sister chromatid segregation	15	-2.401296	*
GO:1901987 regulation of cell cycle phase transition	30	-2.405488	*
GO:1901990 regulation of mitotic cell cycle phase transition	30	-2.405488	*
GO:0050000 chromosome localization	12	-2.405691	*
GO:0006470 protein dephosphorylation	18	-2.445764	*
GO:0007098 centrosome cycle	15	-2.448630	*
GO:0018022 peptidyl-lysine methylation	14	-2.461362	*
GO:0044772 mitotic cell cycle phase transition	35	-2.468455	*
GO:0044770 cell cycle phase transition	36	-2.487258	*
GO:0051983 regulation of chromosome segregation	16	-2.490788	*
GO:0002181 cytoplasmic translation	23	-2.494502	*
GO:0030261 chromosome condensation	17	-2.506977	*
GO:0043484 regulation of RNA splicing	28	-2.514039	*
GO:0048024 regulation of mRNA splicing, via spliceosome	28	-2.514039	*
GO:1902850 microtubule cytoskeleton organization involved in mitosis	35	-2.514714	*
GO:0006401 RNA catabolic process	17	-2.518757	*
GO:0044093 positive regulation of molecular function	32	-2.524733	*
GO:0006475 internal protein amino acid acetylation	25	-2.530423	*
GO:0016573 histone acetylation	25	-2.530423	*
GO:0018393 internal peptidyl-lysine acetylation	25	-2.530423	*
GO:0018394 peptidyl-lysine acetylation	25	-2.530423	*
GO:0071897 DNA biosynthetic process	19	-2.564043	*
GO:0043044 ATP-dependent chromatin remodeling	19	-2.572069	*
GO:2001251 negative regulation of chromosome organization	15	-2.578708	*
GO:0006479 protein methylation	16	-2.592930	*
GO:0008213 protein alkylation	16	-2.592930	*
GO:0061982 meiosis I cell cycle process	25	-2.638776	*
GO:0006473 protein acetylation	27	-2.647222	*
GO:0006277 DNA amplification	12	-2.652135	*
GO:0050684 regulation of mRNA processing	31	-2.666468	*
GO:0006270 DNA replication initiation	12	-2.680627	*
GO:0007127 meiosis I	17	-2.706746	*
GO:0007088 regulation of mitotic nuclear division	26	-2.717261	*
GO:0031123 RNA 3'-end processing	18	-2.732982	*
GO:1903311 regulation of mRNA metabolic process	33	-2.740358	*

Table S3. GSEA analysis on old Smurfs/young Smurfs.

GO:0006281 DNA repair	31	-2.753201	*
GO:0051783 regulation of nuclear division	27	-2.798919	*
GO:0006323 DNA packaging	34	-2.824701	*
GO:0043543 protein acylation	31	-2.886683	*
GO:0007143 female meiotic nuclear division	19	-2.953082	*
GO:0006352 DNA-templated transcription, initiation	27	-2.965346	*
GO:0006367 transcription initiation from RNA polymerase II promoter	27	-2.965346	*
GO:0006310 DNA recombination	17	-2.968695	*
GO:0032259 methylation	22	-3.031190	*
GO:0043414 macromolecule methylation	22	-3.031190	*
GO:0071103 DNA conformation change	38	-3.039650	*
GO:0045132 meiotic chromosome segregation	20	-3.053654	*
GO:0006261 DNA-dependent DNA replication	34	-3.079054	*
GO:0000070 mitotic sister chromatid segregation	34	-3.244982	*

Table S3. GSEA analysis on old Smurfs/young Smurfs. List of the 125 deregulated GO BP categories (adjusted p-value < 0.05) from the GSEA analysis on the list of old Smurf DEGs. Results are partially illustrated in Fig. 4 of the main text. GO BP category: ID and description of the biological process category; size: number of genes annotated in the category; NES: normalized enriched score; p.adjust: FDR correction on the p-value, 0.01 < * < 0.05.

GO BP category	size	NES	p.adjust
GO:0009617 response to bacterium	44	2.077570	**
GO:0009607 response to biotic stimulus	52	2.010078	**
GO:0043207 response to external biotic stimulus	52	2.010078	**
GO:0051707 response to other organism	52	2.010078	**
GO:0019731 antibacterial humoral response	16	1.954582	**
GO:0042742 defense response to bacterium	35	1.953953	**
GO:0009605 response to external stimulus	67	1.942054	**
GO:0050830 defense response to Gram-positive bacterium	20	1.926474	**
GO:0050829 defense response to Gram-negative bacterium	15	1.925644	**
GO:0098542 defense response to other organism	41	1.893139	**
GO:0006952 defense response	53	1.889830	**
GO:0002376 immune system process	43	1.822360	**
GO:0051704 multi-organism process	75	1.800343	**
GO:0006950 response to stress	90	1.783484	**
GO:0019730 antimicrobial humoral response	23	1.760254	*
GO:0006955 immune response	35	1.755081	*
GO:0006959 humoral immune response	26	1.739773	*
GO:0007292 female gamete generation	11	-2.120223	**
GO:0048477 oogenesis	11	-2.120223	**
GO:0022412 cellular process involved in reproduction in multicellular organism	15	-2.142952	*
GO:0007276 gamete generation	16	-2.202907	*
GO:0007281 germ cell development	13	-2.273476	*

Table S4. GSEA analysis on old/young non- Smurfs. List of the 22 significant deregulated GO BP categories (adjusted p-value < 0.05) from the GSEA analysis on the list of old non-Smurf DEGs. Results are illustrated in Fig. 3a of the main text. GO BP category: ID and description of the biological process category; size: number of genes annotated in the category; NES: normalized enriched score; p.adjust: FDR correction on the p-value, * < 0.05, ** < 0.01.

Human symbol	Flybase	Ageing marker	log2FC (DESeq2)	FDR (DESeq2)
A2M	FBgn0041180	others	0.2825784	**
A2M	FBgn0041181	others	0.2390048	**
ADCY5	FBgn0263131	altered intercellular communication	0.4276719	***
ADCY6	FBgn0263131	altered intercellular communication	0.4276719	***
ADCY8	FBgn0024150	altered intercellular communication	0.2249718	*
ADH1B	FBgn0011768	genomic instability	-0.2028574	**
ADH5	FBgn0011768	others	-0.2028574	**
ADIPOR1	FBgn0038984	deregulated nutrient sensing	-0.1933041	**
ADIPOR2	FBgn0038984	deregulated nutrient sensing	-0.1933041	**
AGPAT2	FBgn0026718	deregulated nutrient sensing	0.1087853	*
AKT1	FBgn0010379	cellular senescence	0.1800661	***
AKT2	FBgn0010379	deregulated nutrient sensing	0.1800661	***
AKT3	FBgn0010379	mitochondrial dysfunction	0.1800661	***
ALDH2	FBgn0012036	others	-0.1596763	*
ALDH2	FBgn0051075	others	-0.1747514	**
ALDH9A1	FBgn0051075	others	-0.1747514	**
APP	FBgn0000108	others	0.2690811	*
ARHGAP1	FBgn0036257	others	0.1328514	***
ATG101	FBgn0030960	others	0.1915968	**
BIRC2	FBgn0015247	NF-κB related gene	0.0928874	**
BIRC2	FBgn0260635	NF-κB related gene	0.3378861	***
BIRC3	FBgn0015247	NF-κB related gene	0.0928874	**
BIRC3	FBgn0260635	NF-κB related gene	0.3378861	***
BMI1	FBgn0265623	stem cell exhaustion	0.1405622	**
BSCL2	FBgn0040336	deregulated nutrient sensing	-0.1233342	*
CBX7	FBgn0003042	genomic instability	0.1693354	*
CDC42	FBgn0010341	others	0.1101794	**
CDC42	FBgn0014011	others	0.1457145	*
CDK7	FBgn0263237	others	-0.1098824	*
CEBPA	FBgn0005638	others	0.6106055	**
CEBPA	FBgn0036126	others	0.3134342	***
CEBPB	FBgn0005638	others	0.6106055	**
CEBPB	FBgn0036126	others	0.3134342	***
CREB1	FBgn0265784	genomic instability	0.0909339	**
CREB3	FBgn0004396	loss of proteostasis	0.3022914	*
CREB3L1	FBgn0004396	loss of proteostasis	0.3022914	*
CREB3L2	FBgn0004396	loss of proteostasis	0.3022914	*
CREB3L3	FBgn0004396	loss of proteostasis	0.3022914	*
CREB3L4	FBgn0004396	loss of proteostasis	0.3022914	*
CTNNB1	FBgn0000117	altered intercellular communication	-0.0822029	*
CYCS	FBgn0284248	cellular senescence	-0.1161409	*
DCTN1	FBgn0036882	others	0.4812843	***
DGAT1	FBgn0004797	others	-0.3361186	**
DGAT1	FBgn0037612	others	-0.2345767	**
EGFR	FBgn0003731	cellular senescence	-0.4170074	***
EGR1	FBgn0003499	cellular senescence	0.8073477	***
EIF4EBP1	FBgn0261560	others	0.7687897	***
EPS8	FBgn0038466	others	0.3036899	***
ERBB2	FBgn0003731	others	-0.4170074	***
FADS1	FBgn0032603	others	-0.3277650	***
FBP1	FBgn0032820	others	-0.3958523	**
FGF21	FBgn0014135	deregulated nutrient sensing	0.4182790	*
FGF23	FBgn0014135	others	0.4182790	*
FGF7	FBgn0014135	senescence-associated secretory phenotype	0.4182790	*
FLT1	FBgn0032006	epigenetic alterations	0.2281674	**
FOS	FBgn0001297	cellular senescence	0.5257795	***
GAPDH	FBgn0001091	others	-0.2321337	*
GAPDH	FBgn0001092	others	-0.1092674	*

Table S5. Human genes from Ageing Atlas mapping to Smurf DEGs.

GCK	FBgn0001187	others	-0.6731802	***
GCK	FBgn0042711	others	2.2356232	*
GCLC	FBgn0040319	loss of proteostasis	0.2089071	***
GHRHR	FBgn0260753	deregulated nutrient sensing	-0.2815731	*
GPX4	FBgn0035438	others	0.1982219	*
GSTP1	FBgn0010226	epigenetic alterations	-0.0897638	*
HK3	FBgn0001187	others	-0.6731802	***
HK3	FBgn0042711	others	2.2356232	*
HRAS	FBgn0003206	cellular senescence	0.1123010	*
HSPA1A	FBgn0001217	loss of proteostasis	0.6261976	**
HSPA1A	FBgn0001218	loss of proteostasis	0.2654464	**
HSPA1A	FBgn0001230	loss of proteostasis	2.0776757	***
HSPA1A	FBgn0013275	loss of proteostasis	1.6387273	***
HSPA1A	FBgn0013276	loss of proteostasis	1.5759412	***
HSPA1A	FBgn0013277	loss of proteostasis	2.6948100	***
HSPA1A	FBgn0013278	loss of proteostasis	3.4593216	***
HSPA1A	FBgn0013279	loss of proteostasis	2.9734542	***
HSPA1A	FBgn0051354	loss of proteostasis	3.2535126	***
HSPA1B	FBgn0001217	loss of proteostasis	0.6261976	**
HSPA1B	FBgn0001218	loss of proteostasis	0.2654464	**
HSPA1B	FBgn0001230	loss of proteostasis	2.0776757	***
HSPA1B	FBgn0013275	loss of proteostasis	1.6387273	***
HSPA1B	FBgn0013276	loss of proteostasis	1.5759412	***
HSPA1B	FBgn0013277	loss of proteostasis	2.6948100	***
HSPA1B	FBgn0013278	loss of proteostasis	3.4593216	***
HSPA1B	FBgn0013279	loss of proteostasis	2.9734542	***
HSPA1B	FBgn0051354	loss of proteostasis	3.2535126	***
HSPA8	FBgn0001217	loss of proteostasis	0.6261976	**
HSPA8	FBgn0001218	loss of proteostasis	0.2654464	**
HSPA8	FBgn0001230	loss of proteostasis	2.0776757	***
HSPA8	FBgn0013275	loss of proteostasis	1.6387273	***
HSPA8	FBgn0013276	loss of proteostasis	1.5759412	***
HSPA8	FBgn0013277	loss of proteostasis	2.6948100	***
HSPA8	FBgn0013278	loss of proteostasis	3.4593216	***
HSPA8	FBgn0013279	loss of proteostasis	2.9734542	***
HSPA8	FBgn0051354	loss of proteostasis	3.2535126	***
HSPA9	FBgn0001217	epigenetic alterations	0.6261976	**
HSPA9	FBgn0001218	epigenetic alterations	0.2654464	**
HSPA9	FBgn0001230	epigenetic alterations	2.0776757	***
HSPA9	FBgn0013275	epigenetic alterations	1.6387273	***
HSPA9	FBgn0013277	epigenetic alterations	2.6948100	***
HSPA9	FBgn0051354	epigenetic alterations	3.2535126	***
IGF1R	FBgn0283499	others	0.4196350	***
IKBKG	FBgn0041205	NF-κB related gene	0.1788092	**
INSR	FBgn0283499	cellular senescence	0.4196350	***
IRS1	FBgn0024248	cellular senescence	0.2307325	***
IRS2	FBgn0024248	cellular senescence	0.2307325	***
IRS4	FBgn0024248	altered intercellular communication	0.2307325	***
ITGA2	FBgn0034005	senescence-associated secretory phenotype	0.8217659	**
JUN	FBgn0001291	genomic instability	0.2579762	***
JUND	FBgn0001291	cellular senescence	0.2579762	***
KCNAB3	FBgn0263220	others	0.3437096	***
KL	FBgn0036659	others	1.2840843	***
KRAS	FBgn0003206	others	0.1123010	*
LRP2	FBgn0000119	deregulated nutrient sensing	0.2632794	**
LRP2	FBgn0004649	deregulated nutrient sensing	-0.2395728	**
MAPK14	FBgn0015765	stem cell exhaustion	0.1115323	**
MAPK14	FBgn0024846	stem cell exhaustion	-0.0729681	*
MMP1	FBgn0033438	senescence-associated secretory phenotype	0.3137892	*
MMP1	FBgn0035049	senescence-associated secretory phenotype	0.4040943	***
MMP10	FBgn0035049	senescence-associated secretory phenotype	0.4040943	***

Table S5. Human genes from Ageing Atlas mapping to Smurf DEGs.

MMP12	FBgn0035049	senescence-associated secretory phenotype	0.4040943	***
MMP13	FBgn0035049	senescence-associated secretory phenotype	0.4040943	***
MMP14	FBgn0033438	senescence-associated secretory phenotype	0.3137892	*
MMP14	FBgn0035049	senescence-associated secretory phenotype	0.4040943	***
MMP3	FBgn0035049	senescence-associated secretory phenotype	0.4040943	***
MMP7	FBgn0033438	senescence-associated secretory phenotype	0.3137892	*
MMP7	FBgn0035049	senescence-associated secretory phenotype	0.4040943	***
MSRA	FBgn0000565	others	-0.4061796	*
MYC	FBgn0262656	cellular senescence	0.2856232	***
NFKB1	FBgn0014018	altered intercellular communication	0.6072440	***
NFKB1	FBgn0260632	altered intercellular communication	0.2692880	***
NFKB2	FBgn0014018	others	0.6072440	***
NFKB2	FBgn0260632	others	0.2692880	***
NFKBIA	FBgn0000250	deregulated nutrient sensing	0.2035375	***
NRAS	FBgn0003206	others	0.1123010	*
NRG1	FBgn0003984	cellular senescence	0.5158643	***
PDGFB	FBgn0030964	cellular senescence	0.1417094	*
PDGFRA	FBgn0032006	altered intercellular communication	0.2281674	**
PDGFRB	FBgn0032006	others	0.2281674	**
PDPK1	FBgn0020386	others	0.1321109	***
PEX5	FBgn0023516	others	-0.0973273	*
PPP1CA	FBgn0000711	loss of proteostasis	0.1363220	***
PRDX1	FBgn0033518	others	0.4193860	*
PRDX1	FBgn0033520	others	0.3960598	*
PRDX1	FBgn0033521	others	0.3631889	*
PRDX1	FBgn0038519	others	-0.1277483	**
PRDX1	FBgn0040308	others	-0.1101478	*
PRKACA	FBgn0000489	others	0.3546581	***
PRKACB	FBgn0000489	altered intercellular communication	0.3546581	***
PRKACG	FBgn0000489	others	0.3546581	***
PTGES	FBgn0053178	senescence-associated secretory phenotype	-0.2844010	*
PTPN11	FBgn0000382	others	0.0793331	**
PYCR1	FBgn0015781	others	-0.1159238	**
RAE1	FBgn0034646	others	-0.1450926	**
RB1CC1	FBgn0037363	others	0.1100045	**
RELA	FBgn0260632	altered intercellular communication	0.2692880	***
RELB	FBgn0260632	NF-κB related gene	0.2692880	***
RGN	FBgn0030362	others	-1.1438902	***
RGN	FBgn0038257	others	-0.5406798	***
RORA	FBgn0000448	others	0.4032042	*
SDHC	FBgn0037873	mitochondrial dysfunction	-0.1748854	*
SERPINB2	FBgn0028983	senescence-associated secretory phenotype	0.6323413	***
SERPINB2	FBgn0028988	senescence-associated secretory phenotype	0.5292624	***
SERPINB2	FBgn0033113	senescence-associated secretory phenotype	0.4415197	***
SERPINE1	FBgn0002930	cellular senescence	0.4694551	**
SERPINE1	FBgn0024293	cellular senescence	-0.3417438	*
SERPINE1	FBgn0028983	cellular senescence	0.6323413	***
SERPINE1	FBgn0028988	cellular senescence	0.5292624	***
SERPINE1	FBgn0033113	cellular senescence	0.4415197	***
SERPINE1	FBgn0265137	cellular senescence	0.2572622	**
SHC1	FBgn0015296	others	0.1164009	*
SIRT1	FBgn0024291	epigenetic alterations	0.1087477	*
SIRT3	FBgn0024291	mitochondrial dysfunction	0.1087477	*
SOD1	FBgn0003462	cellular senescence	-0.2014963	*
SOD1	FBgn0033631	cellular senescence	-0.1385804	***
SOD1	FBgn0039386	cellular senescence	-0.6108333	*
SOD2	FBgn0010213	others	-0.1498883	***

Table S5. Human genes from Ageing Atlas mapping to Smurf DEGs.

SQSTM1	FBgn0003231	deregulated nutrient sensing	0.2532735	***
TIMP1	FBgn0025879	loss of proteostasis	0.1959727	*
TIMP2	FBgn0025879	senescence-associated secretory phenotype	0.1959727	*
TLR4	FBgn0026760	NF-κB related gene	0.4731579	***
TLR4	FBgn0032095	NF-κB related gene	-3.5641912	**
TNFSF13B	FBgn0033483	NF-κB related gene	-0.5546250	***
TP53	FBgn0039044	others	0.1052510	*
TP63	FBgn0039044	genomic instability	0.1052510	*
TP73	FBgn0039044	genomic instability	0.1052510	*
TRAF2	FBgn0030748	NF-κB related gene	0.2209799	*
TRPV1	FBgn0086693	others	0.4707670	*
TXN	FBgn0040070	others	0.1124031	***
UBB	FBgn0086558	loss of proteostasis	0.0851291	*
ULK1	FBgn0260945	others	0.1710703	***
VEGFA	FBgn0030964	others	0.1417094	*
VEGFC	FBgn0030964	senescence-associated secretory phenotype	0.1417094	*
WNT2	FBgn0004360	senescence-associated secretory phenotype	0.4079440	**
XIAP	FBgn0015247	NF-κB related gene	0.0928874	**
XIAP	FBgn0260635	NF-κB related gene	0.3378861	***
XRCC5	FBgn0041627	telomere attrition	0.1538227	**
ZMPSTE24	FBgn0034175	genomic instability	0.3685906	*

Table S5. Human genes from Ageing Atlas mapping to Smurf DEGs. A total of 134 (unique) human genes are retrieved by overlapping the 500 human genes annotated in the Ageing Atlas to the Smurf DEGs. Note that in the table some human genes are “duplicated” as they map to more than one fly gene, and the opposite. In total, 121 unique fly genes are found. Human symbol: human gene name; Flybase: Drosophila gene, flybase ID; Ageing marker: ageing marker annotated to the human gene (12 in total defined); log2FC (DESeq2): log2FC estimated by DESeq2 in the Smurf/non-Smurf analysis; FDR (DESeq2): adjusted p-value, FDR method, *** FDR < 0.001, ** FDR < 0.01, * FDR < 0.05.

Human symbol	Flybase	Ageing marker	log2FC (DESeq2)	FDR (DESeq2)
PSAT1	FBgn0014427	others	0.3856655	***
A2M	FBgn0041180	others	0.4872031	*
A2M	FBgn0041181	others	0.6484273	**
A2M	FBgn0041182	others	0.9048751	***
ADCY8	FBgn0024150	altered intercellular communication	0.5602218	*
BIRC2	FBgn0260635	NF-κB related gene	0.2357145	*
BIRC3	FBgn0260635	NF-κB related gene	0.2357145	*
EEF1A1	FBgn0000557	genomic instability	0.6132644	***
GCLC	FBgn0040319	loss of proteostasis	0.2589307	***
HSPA1A	FBgn0001217	loss of proteostasis	0.9303240	*
HSPA1A	FBgn0001230	loss of proteostasis	0.5126339	*
HSPA1A	FBgn0013278	loss of proteostasis	1.0812546	*
HSPA1B	FBgn0001217	loss of proteostasis	0.9303240	*
HSPA1B	FBgn0001230	loss of proteostasis	0.5126339	*
HSPA1B	FBgn0013278	loss of proteostasis	1.0812546	*
HSPA8	FBgn0001217	loss of proteostasis	0.9303240	*
HSPA8	FBgn0001230	loss of proteostasis	0.5126339	*
HSPA8	FBgn0013278	loss of proteostasis	1.0812546	*
HSPA9	FBgn0001217	epigenetic alterations	0.9303240	*
HSPA9	FBgn0001230	epigenetic alterations	0.5126339	*
KL	FBgn0036659	others	0.7809426	**
LRP2	FBgn0000119	deregulated nutrient sensing	0.3253559	*
MAPK14	FBgn0024846	stem cell exhaustion	-0.1847465	*
NFKB1	FBgn0014018	altered intercellular communication	0.4236755	*
NFKB2	FBgn0014018	others	0.4236755	*
PRDX1	FBgn0033518	others	0.5401910	*
PRDX1	FBgn0033521	others	0.5863338	***
PTGS2	FBgn0038469	altered intercellular communication	-3.8056281	*
SERPINB2	FBgn0028988	senescence-associated secretory phenotype	0.6029126	**
SERPINE1	FBgn0002930	cellular senescence	0.8500831	**
SERPINE1	FBgn0028988	cellular senescence	0.6029126	**
SERPINE1	FBgn0033574	cellular senescence	0.8299441	***
SQSTM1	FBgn0003231	deregulated nutrient sensing	0.1530703	*
TIMP1	FBgn0025879	loss of proteostasis	0.3850303	*
TIMP2	FBgn0025879	senescence-associated secretory phenotype	0.3850303	*
WNT2	FBgn0004360	senescence-associated secretory phenotype	0.7601087	*
XIAP	FBgn0260635	NF-κB related gene	0.2357145	*

Table S6. Human genes from Ageing Atlas mapping to non-Smurf DEGs. A total of 25 (unique) human genes are retrieved by overlapping the 500 human genes annotated in the Ageing Atlas to the old non-Smurf DEGs. Note that in the table some human genes are “duplicated” as they map to more than one fly gene, and the opposite. In total, 24 unique fly genes are found. Human symbol: human gene name; Flybase: Drosophila gene, flybase ID; Ageing marker: ageing marker annotated to the human gene (12 in total defined); log2FC (DESeq2): log2FC estimated by DESeq2 in the Smurf/non-Smurf analysis; FDR (DESeq2): adjusted p-value, FDR method, *** FDR < 0.001, ** FDR < 0.01, * FDR < 0.05.

Symbol	log2FC	Effect	% effect	Method	Reference
AhcyL1	0.0501094	increase	13	RNA interference	Parkhitko et al. (2016)
Akt1	0.1800661	decrease	11	RNA interference	Biteau et al. (2010)
alpha-Man-I	0.2894635	increase	39	RNA interference, Mutations	Liu et al. (2009)
Atg1	0.1710703	increase	25	Overexpression	Ulgherait et al. (2014)
Atg8a	0.2575349	increase	56	Overexpression	Simonsen et al. (2008)
Atg8a	0.2575349	increase	50	Overexpression	Park et al. (2009)
Atg8a	0.2575349	decrease	not reported	Mutation	Simonsen et al. (2007)
ATPCL	-0.2800028	increase	32	Mutation	Peleg et al. (2016)
Cbs	-0.1084810	increase	43	Overexpression	Kabil et al. (2011)
Cct1	0.4639772	increase	8	Overexpression	Landis et al. (2003)
cert	0.2351725	decrease	not reported	Mutation	Rao et al. (2007)
CG14207	0.3129048	increase	5	Overexpression	Vos et al. (2016)
CG2789	0.5879365	increase	27	RNA interference	Lin et al. (2014)
CG5389	1.6013534	increase	11	RNA interference	Copeland et al. (2009)
CG9172	-0.1540786	increase	46	RNA interference	Copeland et al. (2009)
CG9940	-0.0911821	increase	not reported	Overexpression	Wen et al. (2016)
chico	0.2307325	increase	48	Knockout	Clancy et al. (2001)
Coq2	0.1882344	increase	31	Mutation	Liu et al. (2011)
dm	0.2856232	decrease	47	Overexpression	Greer et al. (2013)
Eip71CD	-0.4061796	increase	70	Overexpression	Ruan et al. (2002)
Eip71CD	-0.4061796	increase	20	Overexpression	Chung et al. (2010)
elav	0.3066138	decrease	66	Mutation	Toba et al. (2010)
esg	0.3090631	increase	21	Mutation	Magwire et al. (2010)
fabp	-0.2070041	increase	81	Overexpression	Lee et al. (2012)
Gadd45	1.2993997	increase	77	Overexpression	Plyusnina et al. (2011)
Gclc	0.2089071	increase	50	Overexpression	Orr et al. (2005)
GlyP	-0.1294687	increase	17	Post developmental RNA interference	Bai et al. (2013)
GlyS	-0.1051441	increase	10	Post developmental RNA interference	Sinadinos et al. (2014)
Gnmt	0.9499783	increase	not reported	Overexpression	Obata and Miura (2015)
Gpdh	-0.4076777	increase	20	Mutation	Talbert et al. (2015)
Gr63a	3.0206439	increase	30	Deletion	Poon et al. (2010)
GstS1	-0.0897638	increase	33	Overexpression	Simonsen et al. (2008)
Hex-C	-0.6731802	increase	not reported	Mutation	Talbert et al. (2015)
hk	0.1031540	decrease	not reported	Mutation	Simonsen et al. (2007)
Hsc70-3	0.2654464	increase	27	Overexpression	Simonsen et al. (2008)
Hsp27	-0.2242296	increase	30	Overexpression	Wang et al. (2004)
Hsp67Bc	1.4857872	increase	6	Overexpression	Vos et al. (2016)
Hsp68	2.0776757	increase	not reported	Overexpression	Wang et al. (2003)
Hsp68	2.0776757	increase	20	Overexpression	Biteau et al. (2010)
Hsp70Ba	2.6948100	decrease	30	Overexpression	Yang and Tower (2009)
Hsp70Ba	3.4593216	decrease	30	Overexpression	Yang and Tower (2009)
Hsp70Ba	2.9734542	decrease	30	Overexpression	Yang and Tower (2009)
Hsp70Ba	2.6948100	increase	25	Epigenetic modification	Zhao et al. (2005)
Hsp70Ba	3.4593216	increase	25	Epigenetic modification	Zhao et al. (2005)
Hsp70Ba	2.9734542	increase	25	Epigenetic modification	Zhao et al. (2005)
Ilk	0.0852463	increase	63	Mutation	Nishimura et al. (2014)
ImpL2	0.9210441	increase	23	Overexpression	Alic et al. (2011)
InR	0.4196350	increase	85	Mutation	Tatar et al. (2001)
Ire1	0.2114089	decrease	not reported	RNA interference	Luis et al. (2016)
Keap1	0.2687497	increase	10	Mutations	Sykiotis and Bohmann (2008)
l(3)neo18	-0.1273860	increase	24	RNA interference	Copeland et al. (2009)
Lnk	0.1863814	increase	18	Mutations	Slack et al. (2010)
Lnk	0.1863814	increase	33	Mutation	Song et al. (2010)
loco	0.5667916	increase	20	Knockout	Lin et al. (2011)
loco	0.5667916	decrease	20	Overexpression	Lin et al. (2011)
Men	-0.1589187	increase	45	Overexpression	Kim et al. (2015)
Mpk2	0.1115323	decrease	40	Mutation	Vrailas-Mortimer et al. (2011)

Table S7. Drosophila longevity genes (GenAge) mapping to Smurf DEGs.

Mrp4	0.4862356	decrease	47	Mutation	Huang et al. (2014)
Mrp4	0.4862356	increase	16	Overexpression	Huang et al. (2014)
MTF-1	0.1319676	increase	40	Overexpression	Bahadorani et al. (2010)
mth	-0.1491213	increase	35	Mutation	Lin et al. (1998)
mth	-0.1491213	increase	21	Overexpression	Gimenez et al. (2013)
mth	-0.1491213	increase	29	RNA interference	Gimenez et al. (2013)
mys	0.3599439	increase	20	Mutation	Goddeeris et al. (2003)
mys	0.3599439	increase	44	Mutation	Nishimura et al. (2014)
Naam	0.3628227	increase	30	Overexpression	Balan et al. (2008)
ND75	-0.2946195	increase	15	RNA interference	Owusu-Ansah et al. (2013)
Nmdmc	1.1286479	increase	120	Overexpression	Yu et al. (2015)
NPF	0.3933477	decrease	25	Overexpression	Gendron et al. (2014)
p38b	-0.0729681	decrease	60	Mutation	Vrailas-Mortimer et al. (2011)
p53	0.1052510	increase	58	Dominant negative mutation	Bauer et al. (2005)
p53	0.1052510	increase	19	Mutation	Bauer et al. (2007)
Prp19	-0.0842327	increase	25	Overexpression	Garschall et al. (2017)
puc	0.3431643	increase	not reported	Mutation	Wang et al. (2003)
Rbp9	0.1960852	decrease	33	Mutation	Toba et al. (2010)
ry	-0.4315601	decrease	not reported	Mutation	Simonsen et al. (2007)
SdhC	-0.1748854	decrease	22	Dominant negative mutation	Tsuda et al. (2007)
Sir2	0.1087477	increase	57	Overexpression	Rogina and Helfand (2004)
Sir2	0.1087477	decrease	30	RNA interference	Kusama et al. (2006)
Sir2	0.1087477	increase	13	Overexpression	Hoffmann et al. (2013)
Sod	-0.2014963	increase	33	Overexpression	Orr and Sohal (1994)
Sod	-0.2014963	decrease	not reported	Mutation	Phillips et al. (1989)
Sod	-0.2014963	increase	48	Overexpression	Sun and Tower (1999)
Sod2	-0.1498883	decrease	not reported	RNA interference	Kirby et al. (2002)
Sod2	-0.1498883	increase	20	Overexpression	Curtis et al. (2007)
teq	-0.5378343	increase	31	Mutation	Huang et al. (2015)
Thor	0.7687897	increase	20	Overexpression	Demontis and Perrimon (2010)
Thor	0.7687897	increase	22	Overexpression	Zid et al. (2009)
Trx-2	0.1124031	decrease	not reported	Mutation	Svensson and Larsson (2007)
Tsp42Ef	0.2942678	increase	18	Post developmental RNA interference	Bai et al. (2013)
Zw	-0.2204243	increase	38	Overexpression	Legan et al. (2008)

Table S7. Drosophila longevity genes (GenAge) mapping to Smurf DEGs. Drosophila longevity genes (annotated in GenAge) mapping to Smurf DEGs. A total of 58 unique genes are identified. Note that the table contains duplicated gene symbols as multiple experiments can be reported for one gene. Symbol: Drosophila gene symbol; log_2FC: log_2FC Smurf/non-Smurfs estimated by DESeq2; effect: effect of the alteration lifespan; % effect: change in mean lifespan, in %; method: type of experiment performed; reference: reference of the study.

Symbol	log2FC	Effect	% effect	Method	Reference
Atg8a	0.3749200	increase	56	Overexpression	Simonsen et al. (2008)
Atg8a	0.3749200	increase	50	Overexpression	Park et al. (2009)
Atg8a	0.3749200	decrease	not reported	Mutation	Simonsen et al. (2007)
cert	0.2154223	decrease	not reported	Mutation	Rao et al. (2007)
CG14207	0.3336870	increase	5	Overexpression	Vos et al. (2016)
Gclc	0.2589307	increase	50	Overexpression	Orr et al. (2005)
Gnmt	3.3567578	increase	not reported	Overexpression	Obata and Miura (2015)
hk	0.1924967	decrease	not reported	Mutation	Simonsen et al. (2007)
Hsp68	0.5126339	increase	not reported	Overexpression	Wang et al. (2003)
Hsp68	0.5126339	increase	20	Overexpression	Biteau et al. (2010)
Hsp70Ba	1.0812546	decrease	30	Overexpression	Yang and Tower (2009)
Hsp70Ba	1.0812546	increase	25	Epigenetic modification	Zhao et al. (2005)
Men	0.2999653	increase	45	Overexpression	Kim et al. (2015)
Nmdmc	0.9771817	increase	120	Overexpression	Yu et al. (2015)
p38b	-0.1847465	decrease	60	Mutation	Vrailas-Mortimer et al. (2011)

Table S8. Drosophila longevity genes (GenAge) mapping to non-Smurf DEGs. Drosophila longevity genes (annotated in GenAge) mapping to old non-Smurf DEGs. A total of 11 unique genes are identified. Note that the table contains duplicated gene symbols as multiple experiments can be reported for one gene. Symbol: Drosophila gene symbol; log_2FC: log_2FC 20 days non-Smurf/40 days non-Smurfs estimated by DESeq2; effect: effect of the alteration lifespan; % effect: change in mean lifespan, in %; method: type of experiment performed; reference: reference of the study.

Table S9. Linear regression on non-Smurfs gene expression (time dependence).

Flybase	slope	p-value	R squared	symbol	DEGs overlap
FBgn0041579	1.636	8.690e-03	0.578	-	Smurf & old non-Smurf
FBgn0033830	1.582	4.600e-04	0.733	CG10814	Smurf & old non-Smurf
FBgn0038074	1.471	3.300e-04	0.853	Gnmt	Smurf & old non-Smurf
FBgn0038299	0.999	1.960e-03	0.687	Spn88Eb	Smurf & old non-Smurf
FBgn0030310	0.972	2.450e-03	0.541	PGRP-SA	old non-Smurf
FBgn0040972	0.940	1.463e-02	0.541	CG16978	Smurf & old non-Smurf
FBgn0039452	0.877	4.100e-04	0.786	CG14245	Smurf & old non-Smurf
FBgn0033927	0.868	4.900e-04	0.722	-	Smurf & old non-Smurf
FBgn0033926	0.841	1.990e-03	0.674	Arc1	Smurf & old non-Smurf
FBgn0038652	0.780	1.060e-03	0.613	CG7720	Smurf & old non-Smurf
FBgn0034647	0.721	3.390e-03	0.578	pirk	Smurf & old non-Smurf
FBgn0032387	0.670	6.600e-03	0.505	CG16965	old non-Smurf
FBgn0040759	0.668	6.480e-03	0.548	CG13177	Smurf & old non-Smurf
FBgn0033875	0.667	6.060e-03	0.538	CG6357	old non-Smurf
FBgn0031562	0.655	1.215e-02	0.541	CG3604	Smurf & old non-Smurf
FBgn0036951	0.627	1.619e-02	0.505	CG7017	old non-Smurf
FBgn0039593	0.626	1.821e-02	0.551	Sid	Smurf & old non-Smurf
FBgn0039656	0.615	2.400e-03	0.576	-	old non-Smurf
FBgn0030309	0.594	1.680e-03	0.556	CG1572	old non-Smurf
FBgn0033593	0.585	3.748e-02	0.558	Listericin	Smurf & old non-Smurf
FBgn0033397	0.581	2.000e-04	0.810	Cyp4p3	Smurf & old non-Smurf
FBgn0041182	0.576	1.029e-02	0.601	Tep2	old non-Smurf
FBgn0039075	0.572	1.830e-03	0.547	CG4393	old non-Smurf
FBgn0033134	0.568	6.100e-04	0.614	Tsp42El	old non-Smurf
FBgn0035176	0.552	1.000e-02	0.746	CG13905	Smurf & old non-Smurf
FBgn0051769	0.552	2.229e-02	0.519	CG31769	old non-Smurf
FBgn0038088	0.520	3.800e-04	0.741	CG10126	Smurf & old non-Smurf
FBgn0267130	0.513	3.800e-03	0.575	-	Smurf & old non-Smurf
FBgn0010424	0.510	1.550e-03	0.704	TpnC73F	Smurf & old non-Smurf
FBgn0050091	0.502	8.240e-03	0.782	CG30091	old non-Smurf
FBgn0038660	0.484	1.770e-03	0.575	CG14291	old non-Smurf
FBgn0035607	0.478	4.730e-03	0.578	CG4835	old non-Smurf
FBgn0054043	0.474	1.905e-02	0.549	CG34043	Smurf & old non-Smurf
FBgn0250871	0.459	7.650e-03	0.555	pot	Smurf & old non-Smurf
FBgn0041710	0.458	2.236e-02	0.571	yellow-f	Smurf & old non-Smurf
FBgn0020414	0.451	2.120e-03	0.680	Idgf3	Smurf & old non-Smurf
FBgn0262146	0.448	1.190e-03	0.576	MtnE	Smurf & old non-Smurf
FBgn0053258	0.430	2.084e-02	0.620	CG33258	Smurf & old non-Smurf
FBgn0035300	0.427	3.390e-03	0.730	CG1139	old non-Smurf
FBgn0033130	0.424	9.400e-04	0.666	Tsp42Ei	Smurf & old non-Smurf
FBgn0038465	0.417	2.791e-02	0.624	Irc	Smurf & old non-Smurf
FBgn0262717	0.417	1.219e-02	0.645	Skeletor	Smurf & old non-Smurf
FBgn0013305	0.413	2.220e-03	0.555	Nmda1	Smurf & old non-Smurf
FBgn0041337	0.393	2.934e-02	0.543	Cyp309a2	old non-Smurf
FBgn0032699	0.392	1.156e-02	0.606	CG10383	Smurf & old non-Smurf
FBgn0085244	0.392	1.274e-02	0.543	CG34215	Smurf & old non-Smurf
FBgn0041181	0.381	1.264e-02	0.561	Tep3	Smurf & old non-Smurf
FBgn0262003	0.380	2.086e-02	0.571	CG42821	Smurf & old non-Smurf
FBgn0033574	0.373	2.900e-03	0.635	Spn47C	old non-Smurf
FBgn0037447	0.373	2.210e-03	0.805	Neurochondrin	Smurf & old non-Smurf
FBgn0033518	0.371	3.080e-03	0.545	Prx2540-2	Smurf & old non-Smurf
FBgn0038160	0.360	1.984e-02	0.519	CG9759	Smurf & old non-Smurf
FBgn0050082	0.358	1.850e-03	0.539	CG30082	none
FBgn0033521	0.356	3.300e-04	0.712	CG12896	Smurf & old non-Smurf
FBgn0043806	0.352	2.900e-03	0.681	CG32032	old non-Smurf
FBgn0085354	0.352	6.150e-03	0.563	CG34325	old non-Smurf
FBgn0025687	0.350	3.082e-02	0.550	LKRSDH	Smurf & old non-Smurf
FBgn0036461	0.347	7.170e-03	0.704	Zip71B	Smurf & old non-Smurf
FBgn0037796	0.343	2.157e-02	0.568	CG12814	Smurf & old non-Smurf

Table S9. Linear regression on non-Smurfs gene expression (time dependence).

FBgn0039008	0.337	1.716e-02	0.615	CG6972	Smurf & old non-Smurf
FBgn0027563	0.326	1.651e-02	0.588	CG9631	Smurf & old non-Smurf
FBgn0029639	0.323	9.720e-03	0.535	CG14419	old non-Smurf
FBgn0033592	0.320	4.870e-03	0.527	CG13215	Smurf & old non-Smurf
FBgn0013771	0.316	3.480e-03	0.625	Cyp6a9	old non-Smurf
FBgn0031432	0.316	2.033e-02	0.770	Cyp309a1	Smurf & old non-Smurf
FBgn0039094	0.309	3.157e-02	0.611	CG10184	old non-Smurf
FBgn0032908	0.300	1.230e-03	0.698	CG9270	old non-Smurf
FBgn0033289	0.297	2.110e-03	0.698	CG2121	Smurf & old non-Smurf
FBgn0039809	0.297	1.146e-02	0.546	CG15547	old non-Smurf
FBgn0262057	0.297	4.093e-02	0.504	Spn77Ba	Smurf & old non-Smurf
FBgn0033110	0.296	1.230e-03	0.711	CG9447	Smurf & old non-Smurf
FBgn0250835	0.293	7.850e-03	0.590	CG15394	Smurf & old non-Smurf
FBgn0035975	0.287	3.610e-03	0.555	PGRP-LA	Smurf & old non-Smurf
FBgn0033928	0.285	3.890e-03	0.586	Arc2	Smurf & old non-Smurf
FBgn0033520	0.283	7.800e-04	0.609	Prx2540-1	Smurf
FBgn0015037	0.282	4.294e-02	0.667	Cyp4p1	Smurf & old non-Smurf
FBgn0015589	0.278	1.560e-03	0.556	Apc	Smurf
FBgn0032900	0.278	1.440e-02	0.634	CG14401	Smurf & old non-Smurf
FBgn0038651	0.271	2.540e-03	0.537	Epg5	none
FBgn0051793	0.269	3.180e-03	0.700	CG31793	old non-Smurf
FBgn0031547	0.266	2.780e-03	0.652	Sr-CIV	Smurf & old non-Smurf
FBgn0050197	0.265	2.564e-02	0.590	CG30197	old non-Smurf
FBgn0261283	0.264	1.228e-02	0.612	SREBP	Smurf & old non-Smurf
FBgn0259697	0.263	7.950e-03	0.717	nvd	old non-Smurf
FBgn0023129	0.261	6.410e-03	0.767	aay	Smurf & old non-Smurf
FBgn0036449	0.261	9.690e-03	0.539	bmm	old non-Smurf
FBgn0039310	0.261	3.849e-02	0.524	CG11878	old non-Smurf
FBgn0284244	0.254	2.510e-03	0.536	l(2)k05911	none
FBgn0040236	0.253	3.730e-03	0.515	c11.1	Smurf
FBgn0266369	0.253	3.860e-03	0.566	Mtp	Smurf & old non-Smurf
FBgn0032079	0.250	9.560e-03	0.535	CG31886	old non-Smurf
FBgn0265376	0.249	1.710e-03	0.665	-	old non-Smurf
FBgn0033778	0.246	3.170e-03	0.708	CG3790	none
FBgn0053469	0.245	3.530e-03	0.617	CG33469	Smurf & old non-Smurf
FBgn0026255	0.242	1.303e-02	0.682	clumsy	old non-Smurf
FBgn0031910	0.240	3.728e-02	0.615	CG15818	old non-Smurf
FBgn0025881	0.239	1.580e-03	0.555	-	none
FBgn0032601	0.237	4.765e-02	0.617	yellow-b	Smurf & old non-Smurf
FBgn0036640	0.237	3.280e-03	0.725	nxf2	Smurf
FBgn0038000	0.237	7.440e-03	0.700	CG10014	old non-Smurf
FBgn0040212	0.236	1.270e-03	0.630	Dhap-at	none
FBgn0026415	0.231	3.652e-02	0.511	Idgf4	old non-Smurf
FBgn0262107	0.230	2.340e-03	0.621	-	Smurf & old non-Smurf
FBgn0030749	0.227	3.450e-03	0.672	AnxB11	Smurf & old non-Smurf
FBgn0035157	0.227	9.160e-03	0.535	CG13894	Smurf & old non-Smurf
FBgn0011596	0.225	6.740e-03	0.546	fzo	none
FBgn0037560	0.225	2.669e-02	0.528	-	Smurf & old non-Smurf
FBgn0000715	0.219	9.410e-03	0.759	-	Smurf & old non-Smurf
FBgn0036053	0.219	3.330e-03	0.507	iPLA2-VIA	Smurf
FBgn0036448	0.218	9.460e-03	0.540	mop	none
FBgn0050106	0.214	1.163e-02	0.535	CCHa1-R	old non-Smurf
FBgn0031957	0.213	2.750e-03	0.581	TwdlE	Smurf
FBgn0037515	0.212	5.700e-03	0.678	Sp7	old non-Smurf
FBgn0264989	0.212	2.553e-02	0.503	CG44141	old non-Smurf
FBgn0013685	0.211	1.710e-03	0.587	ND6	none
FBgn0260794	0.211	1.485e-02	0.569	ctrip	none
FBgn0053289	0.210	1.912e-02	0.605	ppk5	old non-Smurf
FBgn0266974	0.210	6.200e-04	0.620	-	none

Table S9. Linear regression on non-Smurfs gene expression (time dependence)

FBgn0267073	0.208	1.098e-02	0.692	-	Smurf & old non-Smurf
FBgn0011278	0.205	9.900e-04	0.583	lbe	none
FBgn0032451	0.204	4.300e-04	0.628	spict	Smurf
FBgn0034400	0.204	3.510e-03	0.523	CG15099	Smurf
FBgn0035798	0.204	4.970e-02	0.605	frac	old non-Smurf
FBgn0036364	0.204	2.682e-02	0.734	CG14109	Smurf & old non-Smurf
FBgn0038809	0.204	3.330e-03	0.527	CG16953	none
FBgn0050022	0.204	3.732e-02	0.511	CG30022	Smurf & old non-Smurf
FBgn0032689	0.202	2.990e-03	0.563	CG10413	none
FBgn0034664	0.202	3.865e-02	0.536	CG4377	old non-Smurf
FBgn0037279	0.202	1.000e-04	0.702	CG1129	none
FBgn0050438	0.202	4.681e-02	0.542	CG30438	old non-Smurf
FBgn0264876	0.202	4.350e-03	0.536	-	none
FBgn0029995	0.201	5.100e-04	0.627	CG2256	Smurf
FBgn0030159	0.201	3.999e-02	0.513	CG9689	Smurf & old non-Smurf
FBgn0035498	0.197	2.430e-03	0.521	Fit1	Smurf
FBgn0052313	0.192	4.660e-03	0.591	CG32313	Smurf
FBgn0038804	0.189	5.100e-04	0.695	CG10877	old non-Smurf
FBgn0261570	0.186	1.254e-02	0.532	CG42684	none
FBgn0035589	0.184	1.200e-04	0.755	CHMP2B	Smurf & old non-Smurf
FBgn0032139	0.183	1.541e-02	0.679	CG13116	Smurf & old non-Smurf
FBgn0058354	0.183	2.160e-03	0.537	-	none
FBgn0034221	0.180	6.890e-03	0.646	CG10764	old non-Smurf
FBgn0034513	0.180	3.397e-02	0.564	CG13423	old non-Smurf
FBgn0004897	0.179	1.780e-03	0.669	fd96Ca	none
FBgn0027569	0.179	2.600e-04	0.725	cert	Smurf & old non-Smurf
FBgn0004648	0.178	1.490e-03	0.573	svr	none
FBgn0033889	0.178	2.380e-03	0.731	CG6701	none
FBgn0035097	0.173	2.340e-03	0.607	CG13405	none
FBgn0033063	0.171	1.700e-04	0.752	CG14589	none
FBgn0037683	0.170	1.949e-02	0.732	CG18473	Smurf & old non-Smurf
FBgn0032337	0.168	1.172e-02	0.596	AstCC	none
FBgn0030994	0.167	1.980e-03	0.567	HP1D3csd	none
FBgn0260632	0.167	5.680e-03	0.506	dl	Smurf
FBgn0039266	0.166	1.803e-02	0.626	CG11791	Smurf & old non-Smurf
FBgn0013767	0.164	3.690e-03	0.798	Crz	old non-Smurf
FBgn0033476	0.164	9.650e-03	0.604	oys	Smurf & old non-Smurf
FBgn0036567	0.164	6.900e-04	0.698	CG13074	none
FBgn0266810	0.164	3.172e-02	0.611	-	Smurf & old non-Smurf
FBgn0034184	0.161	1.390e-03	0.561	CG9646	Smurf
FBgn0060292	0.161	1.802e-02	0.504	-	none
FBgn0035875	0.156	1.532e-02	0.528	Cpr66Cb	none
FBgn0266668	0.156	2.900e-03	0.657	Exo84	Smurf & old non-Smurf
FBgn0030452	0.153	2.823e-02	0.540	MFS10	old non-Smurf
FBgn0052071	0.151	1.229e-02	0.541	CG32071	none
FBgn0263118	0.150	1.656e-02	0.805	tx	Smurf & old non-Smurf
FBgn0036956	0.149	3.560e-02	0.501	CG13813	Smurf
FBgn0038455	0.149	1.700e-03	0.644	CG14907	Smurf & old non-Smurf
FBgn0051105	0.149	1.276e-02	0.574	ppk22	Smurf
FBgn0037541	0.148	1.868e-02	0.573	CG2747	none
FBgn0003231	0.147	1.200e-03	0.629	ref(2)P	Smurf & old non-Smurf
FBgn0038320	0.147	1.770e-03	0.659	Sra-1	none
FBgn0266709	0.146	1.723e-02	0.556	Zmynd10	none
FBgn0267708	0.143	1.164e-02	0.508	-	none
FBgn0027843	0.141	1.833e-02	0.652	CAH2	old non-Smurf
FBgn0261983	0.141	1.700e-04	0.689	l(2)gd1	Smurf
FBgn0031897	0.139	1.311e-02	0.593	CG13784	Smurf

Table S9. Linear regression on non-Smurf gene expression (time dependence).

FBgn0051044	0.138	9.780e-03	0.619	-	Smurf
FBgn0267936	0.138	4.150e-03	0.533	-	none
FBgn0259683	0.136	4.424e-02	0.512	Ir40a	none
FBgn0033814	0.133	1.775e-02	0.599	CG4670	Smurf & old non-Smurf
FBgn0027505	0.132	4.390e-03	0.564	Rab3-GAP	none
FBgn0284252	0.132	2.900e-04	0.681	Letm1	Smurf & old non-Smurf
FBgn0010909	0.131	9.110e-03	0.549	msn	Smurf
FBgn0031037	0.130	2.362e-02	0.754	CG14207	Smurf & old non-Smurf
FBgn0260655	0.128	4.402e-02	0.532	l(3)76BDm	none
FBgn0013987	0.127	1.850e-03	0.546	MAPk-Ak2	Smurf
FBgn0034182	0.127	7.410e-03	0.580	SmydA-7	Smurf
FBgn0040319	0.125	1.522e-02	0.702	Gclc	Smurf & old non-Smurf
FBgn0033755	0.124	6.790e-03	0.589	ClC-b	none
FBgn0266186	0.120	1.070e-03	0.574	Vamp7	none
FBgn0031356	0.114	1.470e-03	0.555	CG17660	Smurf
FBgn0259227	0.113	1.455e-02	0.535	CG42327	none
FBgn0035137	0.112	1.538e-02	0.554	CG1233	Smurf & old non-Smurf
FBgn0027554	0.111	4.101e-02	0.544	CG8042	Smurf & old non-Smurf
FBgn0051618	0.111	6.780e-03	0.554	His2A:CG31618	none
FBgn0033735	0.107	3.043e-02	0.520	CG8525	none
FBgn0050372	0.107	7.910e-03	0.536	Asap	Smurf
FBgn0001202	0.104	6.700e-03	0.684	hook	Smurf & old non-Smurf
FBgn0028515	0.104	2.760e-03	0.519	EndoGI	Smurf
FBgn0035165	0.101	1.300e-03	0.699	CG13887	Smurf & old non-Smurf
FBgn0038110	0.094	1.084e-02	0.625	CG8031	Smurf
FBgn0038535	0.089	2.125e-02	0.593	alt	none
FBgn0265001	0.089	3.002e-02	0.503	ppk18	none
FBgn0036666	0.086	1.508e-02	0.621	TSG101	Smurf & old non-Smurf
FBgn0027598	0.084	7.800e-03	0.606	cindr	Smurf
FBgn0025865	0.081	2.559e-02	0.506	Cortactin	none
FBgn0001941	0.072	5.780e-03	0.522	ifc	Smurf
FBgn0029657	0.069	2.251e-02	0.741	CG12535	none
FBgn0029502	-0.075	4.315e-02	0.634	COQ7	none
FBgn0034931	-0.082	4.050e-02	0.534	CG2812	none
FBgn0035586	-0.082	1.494e-02	0.609	Fitm	none
FBgn0031148	-0.093	3.054e-02	0.513	Cbs	Smurf
FBgn0023512	-0.095	2.630e-02	0.550	eIF2Bepsilon	old non-Smurf
FBgn0265540	-0.097	2.383e-02	0.579	-	none
FBgn0050359	-0.103	4.574e-02	0.650	Mal-A5	Smurf & old non-Smurf
FBgn0020258	-0.108	3.522e-02	0.568	ppk	none
FBgn0010213	-0.111	8.210e-03	0.526	Sod2	Smurf
FBgn0024846	-0.114	2.382e-02	0.526	p38b	Smurf & old non-Smurf
FBgn0035155	-0.117	4.582e-02	0.631	RabX6	old non-Smurf
FBgn0260234	-0.119	1.391e-02	0.505	Xport-B	none
FBgn0015527	-0.124	2.122e-02	0.632	peng	Smurf & old non-Smurf
FBgn0027348	-0.126	2.280e-03	0.574	bgm	Smurf
FBgn0033229	-0.126	4.700e-04	0.622	CG12822	none
FBgn0265177	-0.126	2.964e-02	0.514	CG44242	old non-Smurf
FBgn0020248	-0.130	4.160e-03	0.539	stet	none
FBgn0051244	-0.134	2.746e-02	0.536	CG31244	none
FBgn0032444	-0.135	3.560e-02	0.531	CCT4	Smurf & old non-Smurf
FBgn0086665	-0.139	4.589e-02	0.505	-	none
FBgn0030786	-0.140	3.522e-02	0.502	mRpL22	Smurf & old non-Smurf
FBgn0033907	-0.142	2.423e-02	0.507	mRpS16	none
FBgn0027525	-0.144	1.073e-02	0.558	LTV1	none
FBgn0034808	-0.148	5.140e-03	0.621	CG9896	Smurf & old non-Smurf
FBgn0265578	-0.149	2.103e-02	0.506	CG44405	none
FBgn0038017	-0.156	4.171e-02	0.516	CG4115	none
FBgn0267809	-0.157	6.460e-03	0.553	-	none

FBgn0034923	-0.159	5.660e-03	0.584	Upf3	Smurf & old non-Smurf
FBgn0086039	-0.163	4.430e-03	0.562	-	none
FBgn0267691	-0.163	5.130e-03	0.552	-	none
FBgn0086447	-0.166	1.010e-02	0.537	l(2)37Cg	none
FBgn0085192	-0.167	3.141e-02	0.548	CG34163	old non-Smurf
FBgn0031692	-0.192	2.430e-03	0.705	TpnC25D	none
FBgn0052639	-0.193	3.930e-03	0.507	CG32639	none
FBgn0031032	-0.195	9.880e-03	0.521	CG14204	none
FBgn0034323	-0.196	1.310e-03	0.625	CG18537	none
FBgn0030011	-0.201	2.400e-04	0.670	Gbeta5	none
FBgn0029885	-0.202	2.290e-03	0.524	CG3224	none
FBgn0033515	-0.205	1.400e-03	0.564	Ir47a	none
FBgn0266404	-0.206	4.070e-03	0.537	-	none
FBgn0030759	-0.207	2.500e-03	0.552	CG13014	none
FBgn0267191	-0.207	2.223e-02	0.509	-	old non-Smurf
FBgn0030004	-0.208	2.240e-03	0.525	CG10958	none
FBgn0031814	-0.213	5.210e-03	0.507	retm	none
FBgn0032645	-0.213	6.000e-04	0.670	CG15142	none
FBgn0037186	-0.218	3.544e-02	0.542	CG11241	none
FBgn0260484	-0.219	3.780e-03	0.566	HIP-R	Smurf & old non-Smurf
FBgn0038315	-0.231	1.450e-03	0.601	CG14866	none
FBgn0262954	-0.232	6.220e-03	0.531	Rpb12	old non-Smurf
FBgn0036575	-0.234	2.546e-02	0.629	CG5157	Smurf & old non-Smurf
FBgn0030331	-0.235	1.666e-02	0.522	CG15221	Smurf & old non-Smurf
FBgn0031695	-0.240	6.670e-03	0.500	Cyp4ac3	Smurf
FBgn0037788	-0.244	1.470e-03	0.574	CG3940	Smurf
FBgn0040775	-0.247	5.320e-03	0.626	CG12158	Smurf & old non-Smurf
FBgn0039840	-0.249	2.573e-02	0.635	pHCl-2	Smurf
FBgn0004372	-0.257	9.380e-03	0.535	aly	none
FBgn0036362	-0.276	3.090e-03	0.533	CG10725	Smurf
FBgn0040705	-0.286	8.950e-03	0.511	ND-B8	old non-Smurf
FBgn0031865	-0.287	1.750e-02	0.510	Nha1	Smurf & old non-Smurf
FBgn0051104	-0.287	3.289e-02	0.534	CG31104	Smurf
FBgn0032253	-0.296	2.980e-03	0.613	LManI	Smurf & old non-Smurf
FBgn0039325	-0.304	1.796e-02	0.510	CG10560	Smurf & old non-Smurf
FBgn0267164	-0.309	3.900e-04	0.633	-	none
FBgn0022702	-0.310	5.110e-03	0.526	Cht2	old non-Smurf
FBgn0013348	-0.316	7.100e-04	0.599	TpnC41C	none
FBgn0042201	-0.332	8.000e-05	0.830	Nplp3	Smurf & old non-Smurf
FBgn0040725	-0.336	1.080e-03	0.624	CG13946	none
FBgn0085358	-0.337	1.170e-02	0.546	Diedel3	Smurf & old non-Smurf
FBgn0032008	-0.355	3.900e-03	0.600	CG14277	Smurf & old non-Smurf
FBgn0039760	-0.406	7.730e-03	0.546	CG9682	old non-Smurf
FBgn0035887	-0.409	5.690e-03	0.640	Jon66Cii	Smurf & old non-Smurf
FBgn0010241	-0.410	2.917e-02	0.654	Mdr50	none
FBgn0031929	-0.410	2.060e-03	0.532	CG18585	Smurf
FBgn0033188	-0.410	3.851e-02	0.516	Drat	Smurf
FBgn0016675	-0.415	6.500e-04	0.655	Lectin-galC1	Smurf & old non-Smurf
FBgn0034515	-0.420	1.296e-02	0.529	CG13428	Smurf & old non-Smurf
FBgn0038181	-0.441	1.020e-03	0.601	CG9297	old non-Smurf
FBgn0036361	-0.443	2.790e-03	0.525	CG10154	old non-Smurf
FBgn0037996	-0.445	8.000e-04	0.592	CG4830	Smurf
FBgn0085241	-0.474	2.840e-03	0.529	CG34212	old non-Smurf
FBgn0031700	-0.491	7.300e-04	0.606	CG14022	none
FBgn0035734	-0.506	8.100e-04	0.592	CG14823	Smurf

Table S9. Linear regression on non-Smurfs gene expression (time dependence).

FBgn0085241	-0.474	2.840e-03	0.529	CG34212	old non-Smurf
FBgn0031700	-0.491	7.300e-04	0.606	CG14022	none
FBgn0035734	-0.506	8.100e-04	0.592	CG14823	Smurf
FBgn0050042	-0.544	7.180e-03	0.548	Cpr49Ab	Smurf & old non-Smurf
FBgn0038236	-0.609	7.660e-03	0.616	Cyp313a1	Smurf & old non-Smurf
FBgn0040074	-0.634	4.500e-04	0.682	retinin	Smurf & old non-Smurf
FBgn0031176	-0.646	2.143e-02	0.574	whe	Smurf & old non-Smurf
FBgn0000075	-0.661	4.300e-03	0.525	amd	none
FBgn0011555	-0.759	2.100e-04	0.692	thetaTry	Smurf & old non-Smurf
FBgn0037782	-0.832	2.700e-04	0.696	Npc2d	Smurf & old non-Smurf
FBgn0035886	-0.870	1.740e-03	0.613	Jon66Ci	Smurf & old non-Smurf
FBgn0020908	-1.259	0.000e+00	0.952	Scp1	old non-Smurf
FBgn0039777	-1.332	9.640e-03	0.532	Jon99Fii	Smurf & old non-Smurf
FBgn0039778	-1.369	1.698e-02	0.521	Jon99Fi	Smurf & old non-Smurf
FBgn0031277	-2.062	2.200e-04	0.699	CG13947	old non-Smurf

Table S9. Linear regression on non-Smurfs gene expression (time dependence). The 301 genes with significant slope over time in non-Smurfs, with $r^2 > 0.5$. Genes are ordered by descending slope value. Flybase: flybase ID; slope: β_1 of the linear regression; p-value: F-statistic p-value; R squared : r^2 of the estimated linear regression; symbol: Gene symbol; DEGs overlap: specifies if the genes has been detected as significantly deregulated in Smurfs, old non-Smurfs, both or none.

KEGG path	Avg age correlation	Avg Smurf correlation	adjust p-val (Fasano-Franceschini)
dme04624 Toll and Imd signaling pathway	0.080	0.248	5.2e-06
dme00565 Ether lipid metabolism	0.096	0.232	6.3e-03
dme04130 SNARE interactions in vesicular transport	-0.097	0.229	5.8e-04
dme04144 Endocytosis	-0.084	0.207	1.5e-08
dme04213 Longevity regulating pathway - multiple species	0.066	0.166	9.6e-03
dme04391 Hippo signaling pathway - fly	-0.023	0.165	3.5e-02
dme04350 TGF-beta signaling pathway	0.022	0.149	1.9e-02
dme04070 Phosphatidylinositol signaling system	-0.025	0.138	3.9e-02
dme00564 Glycerophospholipid metabolism	0.111	0.134	3.9e-03
dme04745 Phototransduction - fly	0.081	0.129	2.8e-02
dme04068 FoxO signaling pathway	-0.037	0.128	4.6e-03
dme04013 MAPK signaling pathway - fly	-0.028	0.114	1.8e-03
dme00480 Glutathione metabolism	0.054	0.088	1.1e-02
dme01100 Metabolic pathways	0.083	-0.090	3.7e-33
dme00240 Pyrimidine metabolism	0.032	-0.091	9.1e-03
dme00310 Lysine degradation	-0.065	-0.103	1.7e-03
dme00010 Glycolysis / Gluconeogenesis	0.051	-0.143	3.5e-03
dme00620 Pyruvate metabolism	0.053	-0.147	3.8e-03
dme00330 Arginine and proline metabolism	0.073	-0.169	8.3e-03
dme03010 Ribosome	-0.012	-0.191	9.8e-17
dme03008 Ribosome biogenesis in eukaryotes	-0.159	-0.203	4.0e-10
dme01230 Biosynthesis of amino acids	0.124	-0.212	1.2e-08
dme04146 Peroxisome	0.065	-0.214	1.9e-07
dme00450 Selenocompound metabolism	-0.109	-0.216	6.8e-03
dme00190 Oxidative phosphorylation	0.088	-0.217	4.5e-15
dme00270 Cysteine and methionine metabolism	0.150	-0.218	2.5e-05
dme03020 RNA polymerase	-0.186	-0.219	2.3e-04
dme01200 Carbon metabolism	0.064	-0.222	1.7e-11
dme00650 Butanoate metabolism	0.013	-0.225	5.0e-04
dme00981 Insect hormone biosynthesis	0.194	-0.235	1.1e-04
dme04512 ECM-receptor interaction	0.156	-0.235	3.8e-03
dme01040 Biosynthesis of unsaturated fatty acids	0.023	-0.240	1.7e-03
dme00030 Pentose phosphate pathway	0.107	-0.245	4.2e-03
dme00250 Alanine, aspartate and glutamate metabolism	0.209	-0.249	3.1e-05
dme00380 Tryptophan metabolism	0.083	-0.249	8.0e-05
dme00062 Fatty acid elongation	-0.031	-0.255	3.8e-03
dme00220 Arginine biosynthesis	0.214	-0.263	4.0e-03
dme00630 Glyoxylate and dicarboxylate metabolism	0.145	-0.267	2.1e-05
dme00531 Glycosaminoglycan degradation	0.136	-0.270	1.5e-02
dme00020 Citrate cycle (TCA cycle)	0.062	-0.274	1.5e-06
dme00280 Valine, leucine and isoleucine degradation	-0.028	-0.281	9.7e-07
dme01212 Fatty acid metabolism	0.035	-0.281	1.5e-08
dme01210 2-Oxocarboxylic acid metabolism	0.158	-0.299	2.1e-04
dme00640 Propanoate metabolism	0.005	-0.303	1.1e-05
dme00410 beta-Alanine metabolism	0.024	-0.325	2.2e-05
dme00061 Fatty acid biosynthesis	0.216	-0.326	1.6e-03
dme00511 Other glycan degradation	0.116	-0.369	1.3e-03
dme00071 Fatty acid degradation	-0.063	-0.388	4.3e-09

Table S10. KEGG pathways affected by Smurfness. The 48 pathways identified as affected more by Smurfness than chronological age according to our expression dataset. KEGG path: KEGG ID and pathway name; Avg age correlation: average gene expression correlation with chronological age on the genes belonging to the pathway; Avg Smurf correlation: average gene expression correlation with Smurf on the genes belonging to the pathway; adjust pval (Fasano-Franceschini): adjusted p-value (FDR) from the Fasano-Franceschini test.

KEGG path	Avg age correlation	Avg Smurf correlation	adjust p-val (Fasano-Franceschini)
dme00052 Galactose metabolism	0.273	-0.087	1.7e-04
dme00670 One carbon pool by folate	0.261	-0.207	1.7e-03
dme00260 Glycine, serine and threonine metabolism	0.252	-0.196	1.5e-06
dme00830 Retinol metabolism	0.240	-0.099	3.3e-05
dme00053 Ascorbate and aldarate metabolism	0.232	-0.125	1.6e-05
dme00040 Pentose and glucuronate interconversions	0.227	-0.182	5.1e-07
dme00500 Starch and sucrose metabolism	0.219	-0.129	5.5e-05
dme00350 Tyrosine metabolism	0.201	-0.175	1.2e-03
dme02010 ABC transporters	0.177	-0.004	5.3e-03
dme04080 Neuroactive ligand-receptor interaction	0.166	0.027	1.4e-05
dme00051 Fructose and mannose metabolism	0.165	-0.121	1.1e-02
dme00983 Drug metabolism - other enzymes	0.165	0.005	2.8e-08
dme00770 Pantothenate and CoA biosynthesis	0.157	-0.151	1.3e-02
dme00980 Metabolism of xenobiotics by cytochrome P450	0.157	0.050	1.4e-07
dme00561 Glycerolipid metabolism	0.151	-0.071	8.3e-04
dme00982 Drug metabolism - cytochrome P450	0.151	0.069	1.5e-07
dme04142 Lysosome	0.148	-0.128	3.6e-08
dme00230 Purine metabolism	0.144	-0.013	6.9e-03
dme00730 Thiamine metabolism	0.135	0.015	1.3e-02
dme00520 Amino sugar and nucleotide sugar metabolism	0.129	-0.051	7.6e-03
dme00790 Folate biosynthesis	0.123	0.027	3.5e-02
dme00860 Porphyrin and chlorophyll metabolism	0.114	-0.062	2.6e-03
dme04120 Ubiquitin mediated proteolysis	-0.154	0.012	1.5e-06
dme03015 mRNA surveillance pathway	-0.177	-0.066	1.7e-06
dme03018 RNA degradation	-0.191	-0.136	9.1e-09
dme00970 Aminoacyl-tRNA biosynthesis	-0.238	-0.087	1.3e-04
dme03410 Base excision repair	-0.239	0.024	3.0e-03
dme00563 Glycosylphosphatidylinositol (GPI)-anchor biosynthesis	-0.272	-0.118	3.0e-04
dme04330 Notch signaling pathway	-0.275	0.104	1.3e-04
dme03050 Proteasome	-0.276	-0.166	3.5e-09
dme03013 RNA transport	-0.288	-0.117	1.3e-15
dme03040 Spliceosome	-0.288	-0.124	1.5e-17
dme03460 Fanconi anemia pathway	-0.312	0.020	1.3e-06
dme03022 Basal transcription factors	-0.318	-0.096	3.1e-08
dme03420 Nucleotide excision repair	-0.338	-0.073	1.7e-09
dme03440 Homologous recombination	-0.341	-0.005	1.6e-07
dme03430 Mismatch repair	-0.387	-0.081	1.5e-08
dme03030 DNA replication	-0.393	-0.070	2.2e-09

Table S11. KEGG pathways affected by chronological age. The 38 pathways identified as affected more by chronological age than Smurfness according to our expression dataset. KEGG path: KEGG ID and pathway name; Avg age correlation: average gene expression correlation with chronological age of the genes belonging to the pathway; Avg Smurf correlation: average gene expression correlation with Smurf on the genes belonging to the pathway; adjust pval (Fasano-Franceschini): adjusted p-value (FDR) from the Fasano-Franceschini test.

Flybase	Symbol	Avg expression	log2FC	adj p-value
FBgn0038851	dmrt93B	3.07	3.069	1.4e-04
FBgn0003254	rib	3.32	1.965	4.5e-03
FBgn0027788	Hey	1.71	1.841	4.3e-03
FBgn0005660	Ets21C	335.46	1.790	1.4e-09
FBgn0022740	HLH54F	2.87	1.311	2.8e-02
FBgn0035157	CG13894	21.01	1.269	3.6e-07
FBgn0003448	sna	3.83	1.015	2.6e-02
FBgn0034012	Hr51	3.13	0.961	3.1e-02
FBgn0039039	lmd	6.09	0.921	1.0e-02
FBgn0003900	twi	7.96	0.919	3.5e-03
FBgn0003499	sr	107.75	0.807	2.4e-06
FBgn0050401	dany	8.90	0.710	1.7e-02
FBgn0041156	exex	318.94	0.697	4.3e-04
FBgn0005659	Ets98B	398.45	0.686	3.2e-07
FBgn0005638	slbo	44.67	0.611	5.8e-03
FBgn0014018	Rel	5397.15	0.607	9.6e-08
FBgn0035144	Kah	234.96	0.604	1.6e-08
FBgn0014859	Hr38	107.15	0.592	4.5e-03
FBgn0037275	CG14655	86.51	0.568	2.0e-06
FBgn0262477	FoxP	47.50	0.546	3.6e-03
FBgn0001168	h	1066.15	0.536	8.4e-09
FBgn0001297	kay	2357.06	0.526	1.8e-06
FBgn0002576	lz	26.50	0.526	5.1e-02
FBgn0001150	gt	10.25	0.516	3.9e-02
FBgn0004865	Eip78C	64.01	0.513	5.9e-03
FBgn0039808	CG12071	78.47	0.481	3.5e-03
FBgn0028789	Doc1	20.04	0.468	3.2e-03
FBgn0004567	slp2	19.10	0.457	1.4e-02
FBgn0263118	tx	74.98	0.419	2.2e-04
FBgn0023489	Pph13	35.10	0.414	3.5e-02
FBgn0035903	CG6765	43.37	0.410	1.4e-02
FBgn0000448	Hr3	23.74	0.403	3.2e-02
FBgn0024244	drm	325.04	0.364	1.3e-03
FBgn0028979	tio	49.28	0.353	6.3e-04
FBgn0052121	CG32121	69.87	0.339	6.4e-03
FBgn0036126	Irbp18	326.69	0.313	2.1e-07
FBgn0000567	Eip74EF	253.98	0.311	4.8e-03
FBgn0001981	esg	91.47	0.309	3.8e-03
FBgn0035691	CG7386	141.07	0.308	3.1e-02
FBgn0261283	SREBP	6601.83	0.306	3.8e-07
FBgn0004396	CrebA	684.02	0.302	4.3e-02
FBgn0016076	vri	1243.11	0.299	7.9e-04
FBgn0000287	salr	61.93	0.291	4.9e-02
FBgn0262656	Myc	3188.34	0.286	1.8e-04
FBgn0264490	Eip93F	1407.89	0.286	3.6e-02
FBgn0028996	onecut	249.03	0.272	4.8e-02
FBgn0039209	REPTOR	2579.54	0.272	1.3e-07
FBgn0260632	dl	2782.18	0.269	4.2e-07
FBgn0001291	Jra	1533.30	0.258	1.5e-06
FBgn0085432	pan	1160.96	0.253	1.3e-02
FBgn0025525	bab2	389.55	0.249	1.4e-02

Table S12. Transcription factors (TFs) deregulated in Smurfs (DESeq2).

FBgn0042696	NfI	444.20	0.240	1.2e-02
FBgn0004858	elB	207.19	0.233	1.9e-02
FBgn0259938	cwo	1999.53	0.222	9.4e-06
FBgn0038418	pad	491.51	0.216	3.7e-02
FBgn0085424	nub	570.26	0.214	4.6e-02
FBgn0259211	grh	275.93	0.214	3.9e-02
FBgn0021872	Xbp1	7454.95	0.211	7.2e-03
FBgn0032816	Nf-YB	237.31	0.208	6.5e-04
FBgn0043364	cbt	1497.76	0.197	1.2e-03
FBgn0003459	stwl	943.29	0.186	1.8e-02
FBgn0029504	CHES-1-like	1697.41	0.179	1.5e-03
FBgn0033252	CG12769	182.79	0.179	2.3e-02
FBgn0032202	REPTOR-BP	286.80	0.173	2.5e-02
FBgn0030505	NFAT	2029.69	0.157	5.3e-05
FBgn0037877	CG6689	546.74	0.145	1.5e-03
FBgn0037617	nom	361.48	0.132	3.4e-02
FBgn0040305	MTF-1	1288.49	0.132	2.6e-02
FBgn0052296	Mrtf	2068.16	0.131	1.7e-02
FBgn0032512	Bdp1	811.63	0.119	1.3e-02
FBgn0000097	aop	2273.01	0.116	4.5e-02
FBgn0039044	p53	454.53	0.105	3.7e-02
FBgn0035137	CG1233	1289.28	0.100	1.0e-02
FBgn0259176	bun	7445.06	0.098	2.0e-02
FBgn0265784	CrebB	1613.54	0.091	8.6e-03
FBgn0004914	Hnf4	2518.83	0.081	3.0e-02
FBgn0011656	Mef2	1498.21	0.078	1.8e-02
FBgn0014931	CG2678	482.96	-0.112	1.7e-02
FBgn0003963	ush	494.04	-0.204	3.2e-03
FBgn0085405	CG34376	831.14	-0.216	3.3e-02
FBgn0027364	Six4	210.21	-0.248	3.3e-02
FBgn0020912	Ptx1	204.99	-0.289	2.0e-02
FBgn0002609	E(spl)m3-HLH	92.06	-0.297	3.3e-02
FBgn0261930	vnd	167.34	-0.301	5.1e-02
FBgn0005561	sv	103.23	-0.306	2.8e-02
FBgn0036294	CG10654	104.78	-0.341	2.2e-02
FBgn0014343	mirr	89.58	-0.368	2.2e-02
FBgn0004394	pdm2	82.94	-0.378	6.6e-03
FBgn0267978	ap	647.97	-0.411	1.3e-02
FBgn0283451	br	181.95	-0.447	2.7e-03
FBgn0004666	sim	250.09	-0.487	3.9e-06
FBgn0013751	Awh	16.07	-0.498	1.8e-02
FBgn0003117	pnr	27.71	-0.514	3.0e-02
FBgn0015919	caup	64.40	-0.595	4.3e-04
FBgn0030899	Hesr	25.01	-0.597	1.1e-02
FBgn0015904	ara	34.83	-0.622	9.3e-05
FBgn0261963	mid	61.93	-0.695	3.7e-04
FBgn0000964	tj	527.94	-0.701	5.6e-04
FBgn0030005	CG2120	19.15	-0.726	3.6e-04
FBgn0001319	kn	23.68	-0.798	3.4e-02
FBgn0050431	CG30431	179.77	-1.251	3.4e-04
FBgn0039225	Ets96B	5.99	-1.321	1.3e-04

Table S12. Transcription factors (TFs) deregulated in Smurfs (DESeq2). Table summarizing DESeq2 results for the transcription factors deregulated in Smurfs (ordered by log_2FC). Flybase: Flybase gene ID; Symbol: gene symbol; Avg expression: average gene expression across samples provided by DESeq2; log_2FC: log_2FC estimated by DESeq2; adj p-value: FDR corrected p-value provided by DESeq2.

From list of TFs up in Smurfs

Putative regulator	Score	Putative targets
Aef1	6.5	grh, bab2, Eip74EF, kay, Eip93F, sr, Eip78C, CrebB, bun, CG12769, NFAT, Hr38, CG42741, twi CG42741, CG6332, Mrtf Non2, CG2691 NFAT, Hnf4, CG9896, lz, cwo, Mef2, sna, Acp62F Mrtf, drm, elB, nub, h, CHES-1-like, Ets98B, CG43218 Eip78C, CG32121, slp2, CrebA, Mrtf, AGO2, CG5418, Ptth Pph13, cbt
CG4360	6.1	bab2, grh, Eip74EF, kay, Eip93F, sr, Eip78C, CrebB, bun, Hr38, twi CG42741, CG6332, NFAT, Hnf4, CG12769, Mrtf Non2, CG42741, nub, CG9896, CG2691 NFAT, lz, Mef2, sna, cwo, CHES-1-like, Acp62F Mrtf, elB, CG32121, CG12071, CG43218 Eip78C, slp2, drm, AGO2, CrebA, Ets98B, Ptth Pph13, Mrtf, rib
Trl	5.9	bun, tio, elB, Eip74EF, CHES-1-like, nub, bab2, kay, NFAT, Eip78C, Eip93F, cwo, Hr38, esg, sr, rib, sna, exex CG43880, Hnf4, CG12769, cbt, CrebA, drm, Ets98B, CG43248 CrebA, aop, slbo, twi CG42741, CrebB, Mrtf Non2, h, Ets21C, grh, CG12071, vri, Mrtf, pan, Kah, c11.1 lz, slp2
FoxP	5.54	bab2, grh, kay, Eip93F, CrebB, CG9896, Eip74EF, CG6332, CG2691 NFAT, sr, twi CG42741, bun, Eip78C, Mrtf Non2, Hnf4, cwo, CG12769, Hr38, NFAT, lz, Mrtf, CG13894, Ets98B, sna, vri, CG42741, Mef2, MED14, CHES-1-like, nub, elB, CG5418, CycT, CG32121, rib, CG43218 Eip78C, Acp62F Mrtf, CrebA, mthl11
Adf1	5.14	Eip78C, Eip93F, Eip74EF, grh, kay, CG12769, sr, Hey, bab2, slbo, nub, CG5418, bun, Ets98B, CG32121, tio, CG12071, CG13894, lmd, CG42741, CG43248 CrebA, twi CG42741, CG9897, h, slp2, NFAT, Hnf4, drm, CG43218 Eip78C, cwo, salr, aop, vri, CrebA, Hr38
Mef2	4.4	h, sr, bun, Ets98B, Hr38, Mrtf, CG2691 NFAT, cwo, CrebA, sna, Eip78C, bab2, MTF-1, Sardh HLH54F, CG12071, lmd, dl, nub, lz, Kah MED14, kay, esg

From list of TFs down in Smurfs

Putative regulator	Score	Putative targets
Nf-YB	11.9	mirr, caup, ap, sim, Awh, ara, sv, kn, vnd, Ptx1, pdm2, pnr, mid, ush, CG34376, Six4, E(spl)m3-HLH E(spl)m2-BFM, Ets96B CG5805, cbt, tj, Actbeta sv, Ets96B, ara sowah
grn, srp, GATAd, GATAe, pnr	9.6	ush, pnr, CG34376, br, caup, CG34288 CG34376, CG11509 br, pdm2, kn, Ptx1, ara, Awh, CG30431, dor, mirr, ap, cbt, tj, CG2120, sim, vnd

From list of gene up (log2FC > 2) in Smurfs

Putative regulator	Score	Putative targets
Rel	10.1	DptB, AttA, CG9733, edin, AttD CG14323, CG15282, CG13639, Ets21C, CecC CecB, Ddc, CG12858 Dro, CecA1, AttB, CG14743 PGRP-SC1b, upd2, CG43367 Cpr64Ab, CG12009, CG5892, dmrt93B, CG5565, rib, CG43236, e
Hsf	5.9	Hsp70Bbb, Hsp70Aa Hsp70Ab, SMC1 Hsp68, Hsp70Ba

From list of gene down (log2FC < -2) in Smurfs

Putative regulator	Score	Putative targets
Blimp-1	7.2	CG16956 CG43850, CG7675, CG43333, Vm26Aa, trp Jon99Ciii, CG13786
ken	5.4	CG43333, CG16956 CG43850, Vm26Aa, St4, CG7675, CG13786, yellow-e Ir87a, Ir56b
maf-S	4.6	ndl, CG14834, CG13998 Vm26Ab, CG2918 Vml, Yp3, Cp7Fc, CG31928, CG13114, spo, CG16956 CG43850, Vm26Aa, Vm26Ac, yellow-g yellow-g2, Ir7c dec-1, yellow-k mex1

Table S13. i-cisTarget results. The table reports the best hits provided by i-cisTarget when the queries are 1) TFs upregulated in Smurfs, 2) TFs downregulated in Smurfs, 3) genes upregulated in Smurfs ($log_2FC > 2$), genes upregulated in Smurfs ($log_2FC < -2$). In all cases the gene symbol, score and putative detected targets are reported.

Table S14. Longevity screening results. Results are organized by groups according to the way the genes were detected (DESeq2 for the first two groups - up and down in Smurfs- , and i-cisTarget for the last two groups - putative regulators of Smurf TFs). Information about the gene and its alteration (KD or OX) are provided, together with the line

Up in Smurfs

				RU0	RU10			RU50			RU100			RU200		
gene	line	stock	temporal control	ML	ML	% effect	pval	ML	% effect	pval	ML	% effect	pval	ML	% effect	pval
dmrt93B KD	27657	Bloomington	adulthood	76.90	79.92	3.93	9.3e-01	78.12	1.58	4.6e-01	75.85	-1.38	8.1e-02	76.78	-0.16	2.3e-01
dmrt93B KD	27657	Bloomington	development & adulthood	78.99	78.48	-0.65	8.7e-01	76.12	-3.64	1.5e-01	77.61	-1.74	3.8e-01	77.55	-1.83	6.4e-01
dmrt93B OX	F000445	FlyORF	adulthood	81.05	77.50	-4.38	7.1e-05	70.67	-12.80	1.6e-17	64.79	-20.06	2.6e-30	63.20	-22.02	4.8e-34
dmrt93B OX	F000445	FlyORF	adulthood & development	90.90	83.57	-8.06	7.1e-10	67.34	-25.91	2.9e-47	NA	NA	NA	NA	NA	NA
Ets21c KD	39069	Bloomington	adulthood	86.22	61.97	-28.13	1.9e-57	46.36	-46.23	2.1e-66	54.85	-36.39	1.9e-53	47.76	-44.61	3.2e-69
Ets21c KD	39069	Bloomington	development & adulthood	84.23	38.85	-53.87	5.2e-69	NA	NA	NA	NA	NA	NA	NA	NA	NA
Ets21C OX	F000624	FlyORF	adulthood	87.44	46.16	-47.21	2.6e-71	43.93	-49.76	6.7e-72	41.29	-52.78	1.7e-72	41.05	-53.05	1.1e-71
Ets21C OX	F000624	FlyORF	adulthood & development	89.39	46.55	-47.93	1.7e-69	40.19	-55.04	5.5e-71	41.54	-53.52	2.4e-70	41.97	-53.05	8.0e-71
Hey KD	41650	Bloomington	adulthood	86.17	84.78	-1.62	6.4e-01	82.70	-4.04	9.4e-04	85.68	-0.57	3.4e-01	83.08	-3.59	8.9e-02
Hey KD	41650	Bloomington	development & adulthood	77.76	80.72	3.81	4.9e-01	83.39	7.24	6.8e-03	80.99	4.15	1.5e-02	79.11	1.74	2.8e-01
kay KD	27722	Bloomington	adulthood	83.94	64.21	-23.51	1.9e-31	36.96	-55.97	9.4e-58	39.84	-52.53	4.7e-39	34.52	-58.87	9.4e-54
kay KD	27722	Bloomington	development & adulthood	74.74	60.74	-18.73	5.5e-12	46.46	-37.84	4.8e-23	55.78	-25.37	5.9e-04	25.53	-65.84	2.3e-18
Mef2 KD	28699	Bloomington	adulthood	83.57	77.05	-7.80	5.3e-02	63.66	-23.82	1.3e-08	59.73	-28.53	1.1e-09	47.74	-42.87	6.7e-16
Mef2 KD	28699	Bloomington	development & adulthood	85.70	83.98	-2.01	1.3e-01	70.99	-17.17	1.7e-03	67.45	-21.30	9.0e-09	56.81	-33.71	4.3e-18
rib KD	50682	Bloomington	adulthood	84.91	84.24	-0.79	7.1e-03	74.19	-12.63	2.1e-17	72.46	-14.66	3.6e-32	71.51	-15.78	5.1e-38
rib KD	50682	Bloomington	development & adulthood	77.70	82.11	5.68	8.0e-02	72.39	-6.83	3.2e-07	73.55	-5.34	4.3e-10	71.06	-8.54	7.5e-17

Down in Smurfs

				RU0	RU10			RU50			RU100			RU200		
gene	line	stock	temporal control	ML	ML	% effect	pval	ML	% effect	pval	ML	% effect	pval	ML	% effect	pval
Ets96B KD	31935	Bloomington	adulthood	78.71	74.87	-4.87	1.4e-03	76.10	-3.31	1.5e-01	77.63	-1.37	8.5e-01	83.90	6.61	6.4e-15
Ets96B KD	31935	Bloomington	adulthood & development	79.70	78.59	-1.39	8.6e-02	73.22	-8.14	8.9e-04	71.02	-10.89	5.2e-03	84.03	5.44	1.3e-05
Ets96B OX	F000142	FlyORF	adulthood	88.50	86.82	-1.90	3.9e-05	86.20	-2.60	2.0e-03	87.33	-1.33	3.7e-03	89.36	0.98	5.2e-01
Ets96B OX	F000142	FlyORF	adulthood & development	86.35	79.11	-8.39	3.9e-08	74.65	-13.55	2.6e-12	80.56	-6.71	7.5e-06	84.64	-1.98	7.7e-03

Regulating genes up in Smurfs

				RU0	RU10			RU50			RU100			RU200		
gene	line	stock	temporal control	ML	ML	% effect	pval	ML	% effect	pval	ML	% effect	pval	ML	% effect	pval
Adf1 KD	4278	VDRC	adulthood	70.50	75.80	7.57	4.3e-02	72.80	3.25	7.3e-01	71.80	1.81	9.7e-01	72.90	3.39	6.0e-02
Adf1 KD	4278	VDRC	development&adulthood	74.90	80.60	7.52	5.1e-05	74.30	-0.85	4.4e-01	67.30	-10.17	3.5e-03	65.40	-12.75	1.6e-02
Aef1 KD	80390	Bloomington	adulthood	89.64	84.20	-6.07	7.6e-09	80.61	-10.07	9.8e-31	79.41	-11.41	7.0e-33	75.33	-15.97	2.8e-44
Aef1 KD	80390	Bloomington	development & adulthood	82.93	80.87	-2.49	5.1e-03	79.33	-4.35	4.3e-07	75.70	-8.72	1.9e-20	76.87	-7.31	4.2e-17
CG4360 KD	51813	Bloomington	adulthood	75.69	81.20	7.28	4.0e-03	76.16	0.63	3.7e-01	81.16	7.22	6.6e-01	76.27	0.77	3.6e-03
CG4360 KD	51813	Bloomington	adulthood & development	84.82	86.61	2.11	3.2e-01	84.67	-0.18	1.5e-02	87.47	3.12	1.7e-02	84.71	-0.13	2.4e-04
CG4360 OX	F00063	FlyORF	adulthood	81.87	87.03	6.29	3.4e-08	81.78	-0.11	3.0e-03	84.87	3.66	9.4e-09	84.62	3.35	4.9e-07
CG4360 OX	F00063	FlyORF	adulthood & development	83.30	82.24	-1.27	6.8e-01	80.86	-2.92	1.3e-01	58.51	-29.76	8.3e-07	76.19	-8.53	2.8e-01
FoxP KD	26774	Bloomington	adulthood	84.00	77.03	-8.29	5.9e-06	77.97	-7.18	6.1e-07	79.45	-5.42	4.3e-03	80.02	-4.74	9.3e-06
FoxP KD	26774	Bloomington	adulthood & development	78.68	75.94	-3.48	1.0e-02	82.81	5.26	2.7e-01	80.53	2.35	7.3e-01	83.02	5.52	4.4e-02
Hsf KD	41581	Bloomington	adulthood	68.20	62.00	-9.03	6.1e-04	72.70	6.53	2.3e-01	72.50	6.35	6.0e-01	71.10	4.26	9.2e-01
Hsf KD	41581	Bloomington	development&adulthood	65.30	59.60	-8.72	1.1e-02	62.70	-4.06	8.5e-01	57.80	-11.51	1.2e-01	56.00	-14.24	3.4e-05
Hsf OX	F000699	FlyORF	adulthood	86.31	81.64	-5.41	2.6e-06	83.53	-3.21	1.8e-01	83.47	-3.29	3.6e-01	83.66	-3.07	1.1e-03
Hsf OX	F000699	FlyORF	adulthood & development	76.68	76.25	-0.57	8.2e-01	84.61	10.33	1.6e-09	82.57	7.68	7.0e-06	78.95	2.96	1.6e-01
Trl KD	41852	Bloomington	adulthood	78.07	79.75	2.16	2.1e-01	81.90	4.91	7.8e-03	85.51	9.53	8.4e-09	82.38	5.53	8.4e-07
Trl KD	41852	Bloomington	adulthood & development	78.11	73.33	-6.12	8.6e-03	74.95	-4.05	1.3e-01	77.38	-0.93	5.0e-01	77.66	-0.57	8.0e-01

Regulating TFs down in Smurfs

				RU0	RU10			RU50			RU100			RU200		
gene	line	stock	temporal control	ML	ML	% effect	pval	ML	% effect	pval	ML	% effect	pval	ML	% effect	pval
GATAd KD	34625	Bloomington	adulthood	75.84	79.72	5.11	2.4e-01	69.53	-8.33	3.3e-12	71.54	-5.67	1.2e-10	70.95	-6.45	1.7e-06
GATAd KD	F000714	FlyORF	adulthood	85.84	75.95	-11.52	3.8e-17	75.45	-12.10	1.6e-22	68.40	-20.32	3.1e-36	56.62	-34.04	1.2e-59
GATAd KD	34625	Bloomington	adulthood & development	73.26	73.35	0.12	3.3e-02	59.41	-18.90	2.4e-24	65.21	-10.98	8.8e-15	61.22	-16.44	1.8e-22
GATAd KD	F000714	FlyORF	adulthood & development	85.43	59.73	-30.08	1.7e-20	47.07	-44.90	7.8e-64	51.62	-39.57	5.2e-64	54.58	-36.11	1.1e-64
GATAe KD	33748	Bloomington	adulthood	82.43	50.08	-39.25	2.3e-54	46.04	-44.15	6.2e-56	48.30	-41.40	1.0e-49	48.97	-40.60	4.2e-60
GATAe KD	33748	Bloomington	adulthood & development	76.34	46.33	-39.31	7.6e-41	34.48	-54.83	9.1e-53	35.75	-53.17	6.5e-58	33.83	-55.68	7.0e-54
NFyB OX	F001895	FlyORF	adulthood	84.91	84.36	-0.64	2.1e-03	87.36	2.89	5.3e-01	86.12	1.43	3.9e-01	76.09	-10.39	8.4e-18
NFyB OX	F001895	FlyORF	adulthood & development	84.58	77.91	-7.88	4.1e-02	66.81	-21.01	2.0e-30	80.21	-5.16	1.3e-01	76.77	-9.23	4.1e-04
srp KD	28606	Bloomington	adulthood	83.03	82.19	-1.02	2.3e-01	83.89	1.03	3.5e-02	78.39	-5.59	6.9e-03	83.95	1.11	7.7e-01
srp KD	28606	Bloomington	adulthood & development	77.71	74.79	-3.76	1.1e-01	74.98	-3.52	6.8e-01	72.81	-6.31	1.7e-03	77.25	-0.60	2.4e-01
srp OX	F000720	FlyORF	adulthood	77.37	68.84	-11.03	2.0e-21	54.09	-30.09	5.7e-50	44.62	-42.33	9.9e-53	33.56	-56.62	2.0e-58
srp OX	F000720	FlyORF	adulthood & development	71.55	60.85	-14.96	8.7e-10	4.08	-94.29	6.9e-32	9.33	-86.96	9.0e-35	6.18	-91.36	1.8e-32

used and the stock center where the line was bought. Each experiment is either performed during adulthood only or adulthood & developmental (temporal control). Mean lifespan (ML), % effect (% ML change compared to controls) and log-rank p-value are provided for each RU486 condition (RU0 µg/mL - control, RU10 µg/mL, RU50 µg/mL, RU100 µg/mL and RU200 µg/mL). For visual representation of the results, see Fig. S13.
